# Comparative efficacy of drugs with different mechanistic pathways/targets in the treatment of pediatric NAFLD: evidence from a Bayesian network meta-analysis

**DOI:** 10.3389/fphar.2026.1777515

**Published:** 2026-05-25

**Authors:** Ming-Lu Wang, Gao-Qiang Luo, Xin-Ran Guo, Yu-Qiang Liu

**Affiliations:** 1 Department of Pharmacy, Shanxi Provincial People’s Hospital Affiliated to Shanxi Medical University (Shanxi Provincial People’s Hospital), Taiyuan, Shanxi, China; 2 Department of Surgery, Changzhi Luzhou District People’s Hospital, Changzhi, Shanxi, China; 3 Institute of Pharmaceutical and Food Engineering, Shanxi University of Chinese Medicine, Taiyuan, Shanxi, China; 4 Department of Pharmacy, Changzhi People’s Hospital Affiliated to Changzhi Medical College, Changzhi, Shanxi, China

**Keywords:** adolescent, children, drug therapy, hepatic steatosis, network meta-analysis, non-alcoholic fatty liver disease

## Abstract

**Objective:**

Non-alcoholic fatty liver disease (NAFLD) is now the leading cause of chronic liver disease in children and adolescents, and there is no defined treatment for this condition. A network meta-analysis (NMA) was conducted to examine the comparative efficacy of specific mechanism pathways of drugs to achieve improvements in hepatic steatosis in pediatric NAFLD patients.

**Methods:**

Randomized controlled trials regarding pharmacological interventions for pediatric NAFLD patients were obtained. Treatments were classified into energy, inflammation, fibrosis, and gut microbiota modulators. The primary outcome was an improvement in hepatic steatosis, and the secondary outcomes included changes in liver enzymes, lipid profile, and metabolic parameters. Direct meta-analysis and NMA with Bayesian random effects were performed for the extracted data, respectively.

**Results:**

Nineteen RCTs that met the eligibility criteria were identified for this analysis, involving a total of 1,582 pediatric NAFLD patients confirmed by ultrasound or biopsy. Treatment modulating energy (Relative risk (RR): 3.32; 95% Credible intervals (CrI): 1.52–8.26; *P* < 0.05), inflammation (RR: 2.33; 95% CrI: 1.09–5.27; *P* < 0.05) and gut microbiota (RR: 4.60; 95% CrI: 1.42–15.69; *P* < 0.05) were all more effective than placebo in improving hepatic steatosis. Further analysis of secondary outcomes indicated that treatments modulating energy were the optimal agents for reducing serum alanine aminotransferase (ALT) levels (surface under the cumulative ranking curve (SUCRA) = 86.20%), decreasing triglyceride (TG) contents (SUCRA = 91.77%), increasing high-density lipoprotein cholesterol (HDL-C) levels (SUCRA = 94.29%), and improving homeostasis model assessment of insulin resistance (HOMA-IR) (SUCRA = 88.01%). Treatments modulating gut microbiota were the most effective agents for reducing BMI (SUCRA = 96.25%) in pediatric NAFLD patients.

**Conclusion:**

This NMA revealed the relative advantages of drugs that modulate energy in the treatment of pediatric NAFLD, potentially serving as a valuable guide for optimizing drug development and selecting agents for combination therapies.

**Systematic Review Registration:**

https://www.crd.york.ac.uk/prospero/, identifier [CRD42023417431].

## Graphical Abstract

**Figure:**
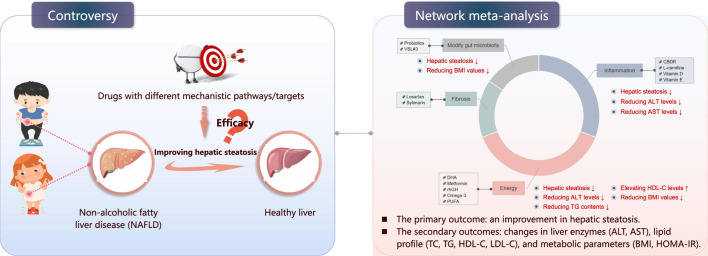

## 1 Introduction

Pediatric non-alcoholic fatty liver disease (NAFLD) is a clinicopathological syndrome occurring in individuals aged <18 years, defined by hepatic fat accumulation exceeding 5% of hepatocytes in the absence of alcohol consumption or other definitive etiologies (Eslam et al., 2025). Its pathogenesis is closely associated with insulin resistance and genetic susceptibility, leading to metabolic stress-induced liver injury (Zhang et al., 2024). The disease spectrum ranges from simple non-alcoholic fatty liver (NAFL) to non-alcoholic steatohepatitis (NASH), along with related liver fibrosis and cirrhosis (Chen et al., 2024). Epidemiological studies estimate its overall prevalence in the general pediatric population at 7.40% (95% credible intervals (CrI): 4.17%–12.81%), rising to 39.17% (95% CrI: 30.65%–48.42%) among obese/overweight individuals (Li et al., 2022). It is agreed that pediatric NAFLD is a serious public health concern due to the longer life expectancy of children and the potential for complications to persist into their adulthood (Vimalesvaran et al., 2024).

The pathogenesis of NAFLD is still poorly understood, making it a critical area of research with the potential to discover innovative treatment options for the disease. Disturbances in lipid metabolism have been widely recognized as the initiating factor in the pathogenesis of NAFLD as a consequence of lipid acquisition (i.e., increased fatty acid uptake and *de novo* lipogenesis) exceeding lipid disposal (i.e., decreased fatty acid β-oxidation and relatively inadequate lipoprotein output) (Santos-Laso et al., 2021; Vetrano et al., 2023). Lipotoxicity associated with lipid overload can subsequently contribute to oxidative stress, mitochondrial dysfunction, and activation of inflammatory pathways, leading to NASH and fibrosis (Bessone et al., 2019; Luo et al., 2022). Despite hepatic steatosis being a prominent characteristic of NAFLD, efforts to reverse this crucial aspect of the disease have been frustrating.

Lifestyle changes (including the adoption of healthier dietary patterns and exercise) are recommended as first-line therapy for pediatric NAFLD (Vos et al., 2017; Bonsembiante et al., 2022). There are no well-established drug therapies for pediatric NAFLD to date, as no drugs have been proven to be beneficial for most patients (Vos et al., 2017; Fan et al., 2019). The rising prevalence and global burden of pediatric NAFLD, underscore an urgent need for effective and safe pharmacotherapies, particularly for individuals unable to sustain lifestyle interventions (Hartmann et al., 2023). Numerous randomized controlled trials (RCTs) have evaluated various drugs and supplements for pediatric NAFLD, yielding heterogeneous results (Akcam et al., 2011; Lavine et al., 2011; Nobili et al., 2011; Ghergherehchi et al., 2013; Alisi et al., 2014; Shiasi Arani et al., 2014; Boyraz et al., 2015; Janczyk et al., 2015; Pacifico et al., 2015; Schwimmer et al., 2016; Famouri et al., 2017a; Famouri et al., 2017b; Namakin et al., 2021; Saneian et al., 2021; El Amrousy et al., 2022; Homaei et al., 2022; Rodrigo et al., 2022; Vos et al., 2022; Xue et al., 2022). Given that, it is imperative to optimize the improvement in outcomes in hepatic steatosis by targeting key mechanistic pathways in pediatric NAFLD to serve as the backbone of combination therapies. Despite attempts to develop drugs targeting these pathways to intervene in the progression of pediatric NAFLD in response to its complex pathophysiological mechanisms, direct comparisons of efficacy between different mechanistic pathways/targets are still lacking. To bridge this gap, a network meta-analysis (NMA) was performed to investigate the comparative efficacy of specific mechanism pathways/targets of drugs in improving hepatic steatosis in pediatric NAFLD patients.

## 2 Methods

This study was reported following the PRISMA extension statement for reporting systematic reviews that incorporate NMAs of healthcare interventions (Hutton et al., 2015; Page et al., 2021) and was conducted in strict accordance with a pre-established study protocol (PROSPERO registration number: CRD42023417431).

### 2.1 Eligibility criteria

Studies eligible for this NMA met the following criteria: (i) population: children or adolescents with ultrasound- or biopsy-proven NAFLD (whether or not combined with obesity, or diabetes); (ii) intervention: monotherapy at any dose or duration in the trial arm; (iii) comparator: an alternative active agent (or placebo) at any dose or duration in the control arm; (iv) outcomes: the primary outcome was an improvement in hepatic steatosis (at least 1-grade reduction in the sonographic grade of fatty liver without worsening of fibrosis); the secondary outcomes included changes in liver enzymes [alanine aminotransferase (ALT), aspartate transaminase (AST)], lipid profile [total cholesterol (TC), triglyceride (TG), low-density lipoprotein cholesterol (LDL-C), high-density lipoprotein cholesterol (HDL-C)], and metabolic parameters [body mass index (BMI), homeostasis model assessment of insulin resistance (HOMA-IR)]; and (v) study design: RCTs.

Studies that fulfilled the following criteria were excluded from this analysis: (i) patients who had undergone liver transplantation or had other etiologies of liver disease (e.g., autoimmune liver disease, viral hepatitis, drug-induced liver injury, biliary tract disease, etc.); (ii) patients with a history of inherited metabolic disease; (iii) studies that were not comparative drug effectiveness; (iv) studies containing combinations of drugs in the same treatment arm; (v) preclinical studies involving only animal and cellular models; (vi) reviews, commentaries, conference abstracts, research proposals, editorial notes, letters to the editor, guidelines, and expert consensus; (vii) duplicate publications; and (viii) literature for which the full text was inaccessible.

### 2.2 Search strategy

Literature was retrieved online through electronic databases including Ovid MEDLINE, EMBASE, Cochrane Central Register of Controlled Trials (CENTRAL) in the Cochrane Library, Pubmed, Web of Science, and Scopus. The main search terms were as indicated below: “fatty liver”, “Non-alcoholic Fatty Liver Disease”, “Nonalcoholic Steatohepatitis”, “NAFLD”, “non-AFLD”, “child”, “infant”, and “adolescent”. To ensure the highest quality of evidence, results were filtered to include only “clinical trial”, “controlled clinical trial”, and “randomized controlled trial” publication types. The search timespan was from database inception to 3 January 2023 and updated the search results till 27 October 2025. Additionally, hand-searching by two investigators (WML, LGQ) on references for this NMA final inclusion trial or relevant systematic reviews was also performed in conjunction with database searches. The detailed search strategies adopted for this analysis can be referred to Supplementary File 1.

### 2.3 Literature screening and data extraction

Two investigators (WML, LGQ) independently screened the titles and abstracts of identified records against the eligibility criteria. Potentially relevant articles then underwent full-text review and cross-verification. Any discrepancies regarding inclusion were resolved through discussion or adjudication by a third experienced investigator (LYQ). Two investigators (WML, LGQ) independently extracted and cross-checked data using a pre-designed data extraction form. The extracted information included: (i) basic study characteristics; (ii) diagnostic criteria and baseline characteristics of the study population; (iii) specific interventions, treatment/evaluation periods, and follow-up durations in the trial and control groups; (iv) drop-out numbers and reasons; (v) information for risk-of-bias assessment; and (vi) outcomes of interest and reported data. For continuous outcomes (liver enzymes, lipid profile, and metabolic parameters), all analyses were based on the change from baseline to follow-up. For studies that directly reported the mean deviation (MD) from baseline and its corresponding standard deviation (SD), these values were extracted without further imputation. A total of eight RCTs (Akcam et al., 2011; Lavine et al., 2011; Nobili et al., 2011; Shiasi Arani et al., 2014; Schwimmer et al., 2016; Namakin et al., 2021; Saneian et al., 2021; Vos et al., 2022) provided directly reported change value from baseline to follow-up. For the remaining studies that did not report change value but provided complete baseline and follow-up mean values and SDs, the SD of the change score (SD_change_) was calculated using the standard formula recommended by the Cochrane Handbook for Systematic Reviews of Interventions (Higgins et al., 2024) (Equation 1). A baseline to follow-up correlation coefficient of 0.5 was pre-specified for the primary analysis, which is the most widely validated and empirically accepted assumption for meta-analyses of chronic disease interventions including pediatric NAFLD clinical trials. For studies that only reported median and interquartile range/range for continuous outcomes, the method proposed by Wan et al. was applied to estimate the corresponding mean and SD (Wan et al., 2014).

$${SD}_{change}=\sqrt{{SD}_{baseline}^{2}+{\;SD}_{final}^{2}\;‐\;2\times r\times {SD}_{baseline}\times {SD}_{final}}$$ (1)

Where *SD*_*baseline*_ and *SD*_*final*_ represent the SDs of baseline and post-treatment values, respectively, *r* denotes the correlation coefficient between baseline and post-treatment values.

To ensure comparability across included studies, the units of all continuous outcomes were harmonized. For studies that reported SD in their original units, the SD were proportionally converted using the same conversion factors applied to the corresponding outcome values, ensuring consistency in the analytical framework.

Safety assessment data were also extracted from the included RCTs, including any reported adverse events (AEs), the number of patients affected, and discontinuations due to adverse events.

### 2.4 Classifications of treatment

Given the ongoing controversy surrounding the effectiveness of certain drugs in treating pediatric NAFLD, based on the pathophysiology and mechanical drivers of NAFLD (Gerges et al., 2021) and referring to a previous report by Ng et al. (2022), the treatments included in this study were divided into four categories following their mechanistic pathways/targets: (1) energy; (2) inflammation; (3) fibrosis; and (4) gut microbiota modulators for subsequent data synthesis in Bayesian NMA. For interventions with pleiotropic effects across multiple targets/pathways, a hierarchical classification rule was pre-specified in our study protocol to minimize subjective bias and ensure full reproducibility of the analysis. The primary regulatory pathway was first determined based on the specific agent’s well-established pharmacological profile, as well as the mechanism most widely reported and validated in pediatric NAFLD RCTs, systematic reviews, and international clinical practice guidelines. Given the distinct pathophysiological characteristics of pediatric NAFLD, this pediatric-specific evidence was prioritized over data from adult cohorts or *in vitro/in vivo* studies. Where the primary mechanism remained ambiguous or equally weighted across targets/pathways, priority was given to the mechanism with a direct causal link to the primary outcome of this network NMA, namely improvement in hepatic steatosis. Any residual classification ambiguity was resolved via consensus conclusions from high-quality systematic reviews and meta-analyses focused exclusively on pediatric NAFLD. In strict accordance with this pre-specified rule, each multi-target agent was assigned to the category corresponding to its primary therapeutic mechanism in pediatric NAFLD. A complete mapping table of all included agents to their corresponding targets/mechanistic nodes were outlined in Supplementary File 2.

To assess the robustness of the pathway-level findings, we performed two pre-specified sensitivity analyses. First, we repeated the NMA after excluding multi-target agents with borderline or overlapping mechanisms. Second, we constructed an additional NMA following the reclassification of multi-target agents in strict accordance with our hierarchical classification rule. These analyses were conducted to verify that the primary conclusions of our study were not spurious findings or artifacts introduced by our predefined pathway-based grouping strategy.

### 2.5 Quality assessment

The quality of the included RCTs was assessed independently by two investigators (WML, LGQ) using the risk of bias assessment tool recommended by the Cochrane Collaboration (Higgins et al., 2011). This tool evaluates six domains: selection, performance, detection, attrition, reporting, and other potential biases. Disagreements were resolved through discussion or by consultation with a third experienced investigator (LYQ).

### 2.6 Statistical analysis

STATA software (Stata Statistical Software Release 14; Stata Corp, USA) was used to make direct pairwise meta-analyses and to draw forest plots. Heterogeneity was assessed using the I2 statistic, where an I2 value greater than 50% indicates substantial heterogeneity. The network of interventions was visualized using network plots in STATA, and the risk of bias across studies was summarized using RevMan (version 5.3).

Given the limited number of trials that allowed for direct comparisons, the present study employed indirect comparisons to investigate the variations in efficacy among treatments. To conduct these indirect comparisons, the present study was based on a Bayesian framework using R (version 4.3.0) software loaded with the gemtc package in conjunction with JAGS software to perform an NMA. Vague (non-informative) priors were specified for all model parameters: a normal prior with mean 0 and precision 0.001 (variance 1000) for treatment effects, and a uniform prior Uniform (0, 5) for the between-study heterogeneity standard deviation. Four Markov chains were run simultaneously. The Bayesian pre-iteration parameter was set to 10,000 to anneal the initial values, followed by a further 50,000 iterations to achieve convergence of the model. Convergence was considered satisfactory when the potential scale reduction factors (PSRF) reached 1. Otherwise, the number of iterations continued to increase. The node splitting method was used to examine inconsistency when there was a closed loop in the network evidence diagram, and the assumption of consistency between direct and indirect evidence was judged to be satisfied when the *P*-value >0.05, and the consistency model could be used for subsequent data analysis. Relative risk (RR) and weighted mean difference (WMD) were used to estimate pooled effect sizes for dichotomous and continuous events, respectively, and the corresponding 95% CrI was calculated. The relative ranking of interventions for the primary and secondary outcomes in this NMA was achieved by ranking the surface under the cumulative ranking curve (SUCRA), with higher SUCRA values (0%–100%) indicating that the treatment option was more likely to be the optimal intervention (Seitidis et al., 2022).

To address heterogeneity in the assessment of the primary outcome (hepatic steatosis improvement) across the included RCTs, we performed two additional sensitivity analyses. First, a stratified NMA was conducted according to the assessment modality used to define the primary outcome: (i) ultrasound-based steatosis grading, and (ii) histology (biopsy)-based assessment. This analysis aimed to determine whether the comparative efficacy estimates varied systematically by measurement method. Second, to account for potential differences in follow-up duration, we conducted a sensitivity analysis limited to trials with a follow-up duration of at least 24 weeks. In addition, to verify that our results were not driven by the pre-specified correlation coefficient assumption, we conducted a pre-specified sensitivity analysis using two plausible alternative correlation values (0.3 and 0.7), which span the full range of correlation coefficients reported in peer-reviewed pediatric NAFLD clinical trials.

## 3 Results

### 3.1 Baseline characteristics and quality assessment

**FIGURE 1:**
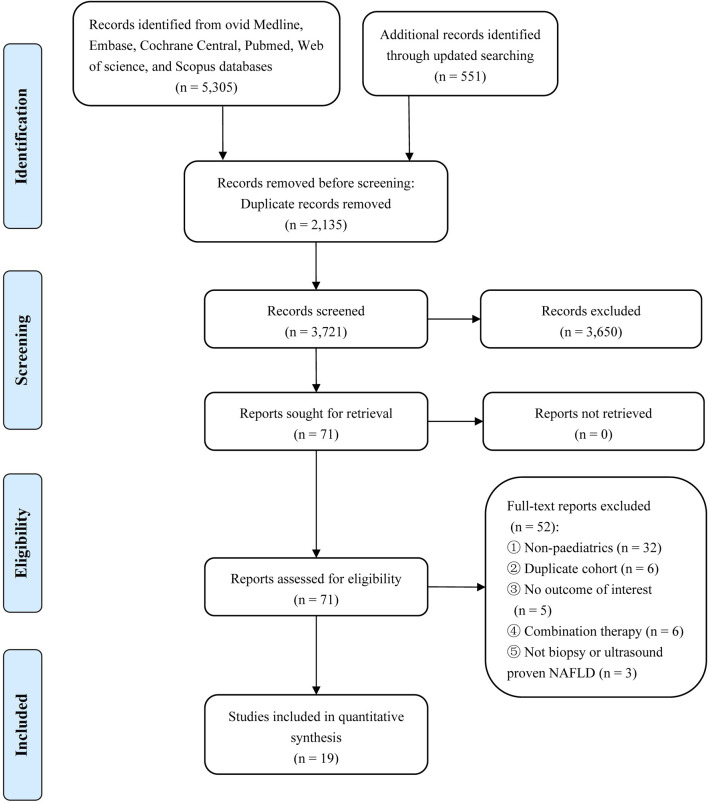
Flow chart for literature screening.

A total of 5,856 records were initially retrieved, with 3,721 remaining after duplicate removal. From these, 3,650 irrelevant papers were excluded after an initial screening, and 71 papers were then retained for re-screening by reviewing the full text. Eventually, a total of 19 RCTs (Akcam et al., 2011; Lavine et al., 2011; Nobili et al., 2011; Ghergherehchi et al., 2013; Alisi et al., 2014; Shiasi Arani et al., 2014; Boyraz et al., 2015; Janczyk et al., 2015; Pacifico et al., 2015; Schwimmer et al., 2016; Famouri et al., 2017a; Famouri et al., 2017b; Namakin et al., 2021; Saneian et al., 2021; El Amrousy et al., 2022; Homaei et al., 2022; Rodrigo et al., 2022; Vos et al., 2022; Xue et al., 2022), comparing pharmacological interventions in pediatric NAFLD, met the eligibility criteria and were included in the analysis (Figure 1).

**FIGURE 2:**
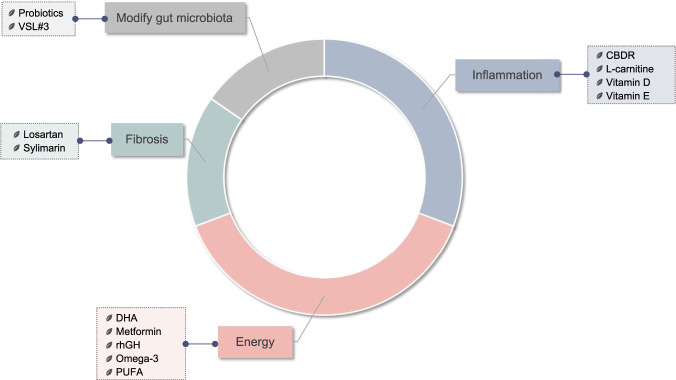
Diagrammatic representation of the classification of pharmacological interventions. (VSL#3: A mixture of eight probiotic strains (*Streptococcus* thermophilus, bifidobacteria [B. breve, B. infantis, B. longum], *Lactobacillus* acidophilus, L. plantarum, L. paracasei, and L. delbrueckii subsp. bulgaricus); DHA: Docosahexaenoic acid; rhGH: Recombinant human growth hormone; PUFA: Polyunsaturated fatty acid; CBDR: Cysteamine bitartrate delayed release).

These 19 RCTs enrolled a total of 1,582 pediatric NAFLD patients confirmed by ultrasound or biopsy. Of these studies, fourteen were two-arm trials (Ghergherehchi et al., 2013; Alisi et al., 2014; Boyraz et al., 2015; Janczyk et al., 2015; Pacifico et al., 2015; Schwimmer et al., 2016; Famouri et al., 2017a; Famouri et al., 2017b; Namakin et al., 2021; Saneian et al., 2021; El Amrousy et al., 2022; Rodrigo et al., 2022; Vos et al., 2022; Xue et al., 2022), four were three-arm trials (Akcam et al., 2011; Lavine et al., 2011; Nobili et al., 2011; Homaei et al., 2022) and one was a four-arm trial (Shiasi Arani et al., 2014). A total of thirteen pharmacological interventions were involved, with nine experimental groups assessing drugs classified in the energy subset (Akcam et al., 2011; Lavine et al., 2011; Nobili et al., 2011; Shiasi Arani et al., 2014; Boyraz et al., 2015; Janczyk et al., 2015; Pacifico et al., 2015; Homaei et al., 2022; Xue et al., 2022), nine in the inflammation subset (Akcam et al., 2011; Lavine et al., 2011; Ghergherehchi et al., 2013; Shiasi Arani et al., 2014; Schwimmer et al., 2016; Namakin et al., 2021; Saneian et al., 2021; El Amrousy et al., 2022; Homaei et al., 2022), two in the fibrosis subset (Famouri et al., 2017a; Vos et al., 2022) and three in the modulate gut microbiota subset (Alisi et al., 2014; Famouri et al., 2017b; Rodrigo et al., 2022) (Figure 2). The majority of the included RCTs used a placebo as the control except for one study (Shiasi Arani et al., 2014) had vitamin E as a control. As lifestyle change is the cornerstone of pediatric NAFLD treatment, all participants received concomitant lifestyle interventions (structured diet and exercise). The basic characteristics of the included studies are summarized in Table 1.

**TABLE 1:** Characteristics of included studies.

Author, year	Study location	Basic characteristics of patients				Intervention		Concomitant diet/Exercise	Treatment/Evaluation period	Follow-up Duration	Drop-out (N)
		Patients population	Sample size (M/F)	Age (years)	NAFLD diagnosis	Intervention group	Control group				
Xue et al (2022)	China	Boys with obesity and NAFLD aged 8–16 years	60 (60/0)	11.76 (1.67)	Ultrasound	rhGH: 0.1 IU/kg/d (n = 30)	Control: no treatment (n = 30)	Diet and exercise guidance	6-mouths	6-mouths	16
Vos et al (2022)	USA	Children with biopsy-proven NAFLD.	83 (67/16)	13 (9–17)^1^	Biopsy	Losartan: 100 mg/d (n = 43)	Placebo (n = 40)	Written nutrition and exercise recommendations	6-mouths	9-mouths	16
Rodrigo et al (2022)	Sri Lanka	Obese children with NAFLD/NASH	84 (62/22)	Overall: NA Probiotic group: 11.28 (1.87) Placebo group: 12.05 (1.45)	Ultrasound	Probiotics: BioKult 14 strain probiotic capsule (children <12 years: 1 capsule/d, children 12–15 years: 2 capsules/d) (n = 43)	Placebo: a capsule without probiotic strains (n = 41)	Structured diet plus physical activity	6-mouths	6-mouths	8
Homaei et al (2022)	Iran	Non-diabetic obese children with NAFLD aged 10–14 years	150 (79/71)	Overall: NA Metformin group: 11.8 (1.6) Vitamin E group: 12.1 (1.7) Placebo group 12.2 (1.5)	Ultrasound	① Metformin: 1,000 mg/d (n = 50) ② vitamin E: 800 IU/d (n = 50)	Placebo (n = 50)	Recommendations for changing lifestyle, exercising, and diets appropriate for weight loss	3-months	3-months	0
El Amrousy et al (2022)	Egypt	Obese children with NAFLD	100 (49/51)	Overall: NA Vitamin D group 9.6 (7.1–16.2)^1^ Placebo group 9.7 (6.5–17.5)^1^	Biopsy	Vitamin D: Vitamin D3 2000 IU/d; (n = 50)	Placebo (n = 50)	Hypocaloric diet where 30% of the total energy intake was reduced	6-mouths	6-mouths	0
Saneian et al (2021)	Iran	Children with NAFLD aged 5–15 years	55 (42/13)	Overall: NA L-carnitine group: 12.3 (2.04) Placebo group: 12.9 (2.41)	Ultrasound	L-carnitine: L-carnitine tablet 50 mg/kg/d; (n = 30)	Placebo (n = 25)	Recommendations for exercise, weight control and low fat diet	3-mouths	3-mouths	7
Namakin et al (2021)	Iran	Obese children with NAFLD aged 12–18 years	101 (54/47)	Overall: NA Vitamin D group: (12–18)^2^ Placebo: (12–18)^2^	Ultrasound	Vitamin D: Vitamin D capsules, a 50,000 U perl once a week (n = 51)	Placebo (n = 50)	NR	3-mouths	3-mouths	0
Famouri et al (2017b)	Iran	Obese children with sonographic NAFLD.	64 (32/32)	Overall: NA Probiotic group 12.7 (2.2) Placebo group 12.6 (1.7)	Ultrasound	Probiotic: Probiotic capsule, qd. (n = 32)	Placebo (n = 32)	Healthy lifestyle habits were recommended for participants of both groups	3-mouths	3-mouths	0
Famouri et al (2017a)	Iran	Children and adolescents aged 5–16 years with NAFLD	NR	Overall: NA Sylimarin group 11.8 (3.0) Placebo group 10.5 (3.2)	Ultrasound	Sylimarin: silymarin tablets 5 mg/kg/d, tid (n = 20)	Placebo (n = 20)	Changing life style including exercise was advised in both groups	3-mouths	3-mouths	0
Schwimmer et al (2016)	USA	Children aged 8–17 years with moderate to severe NAFLD (NAS of 4 or higher)	169 (119/50)	13.7 (2.7)	Biopsy	CBDR: CBDR capsules 9–12 mg/kg/d, bid (n = 88)	Placebo (n = 81)	Standardized nutrition and exercise intervention	13-mouths	19-mouths	15
Pacifico et al (2015)	Italy	Children with biopsyproven NAFLD	51 (30/21)	Overall: NA DHA group 11.0 (2.6) Placebo group 10.8 (2.8)	Biopsy	DHA: 250 mg/d (n = 29)	Placebo (n = 29)	A balanced low-calorie diet and a moderate daily exercise program	6-mouths	6-mouths	7
Janczyk et al (2015)	Polish	Overweight/obese children with NAFLD	76 (65/11)	13 (11.1–15.2)^1^	Ultrasound	Omega-3^4^: 450–1,300 mg/d, bid (n = 30)	Placebo^5^ (n = 34)	An individually prescribed diet, and increased physical activity	6-mouths	6-mouths	12
Boyraz et al (2015)	Turkey	Obese adolescents with NAFLD	138 (73/65)	13.9 (3.7)	Ultrasound	PUFA: 1,000 mg/d, qd (n = 56)	Placebo (n = 52)	Diet and lifestyle intervention	12-mouths	12-mouths	30
Shiasi et al (2014)	Iran	Obese children with NAFLD	119 (57/62)	10 (3.19)	Ultrasound	① Metformin group 1: 1 g/d (age <12 years) (n = 36) ② Metformin group 2: 1.5 g/d (age ≥12 years) (n = 28)	① vitamin E group 1: 400 IU/d (n = 28) ② vitamin E group 1: 800 IU/d (n = 27)	Diet and lifestyle recommendations	2-months (or plus 2 more mouths)^6^	4-mouths	9
Alisi et al (2014)	Italy	Obese children with biopsy-proven NAFLD	44 (24/20)	Overall: NA VSL#3 group 10 (9–12)^1^ Placebo group 11 (10–12)^1^	Biopsy	VSL#3: VSL#3 1–2 sachet/d (n = 22)	Placebo (n = 22)	A low calorie diet and a moderate programme of aerobic exercise	4-mouths	4-mouths	4
Ghergherehchi et al (2013)	Iran	Obese children with NAFLD	33 (18/15)	7.41 (3.21)	Ultrasound	Vitamin E: 400 mg/d (n = 17)	Placebo (n = 16)	Lifestyle intervention and physical activity	6-mouths	6-mouths	0
Nobili et al. (2011)	Italy	Children with biopsy-proven NAFLD	60 (25/35)	Overall: NA DHA group 1 11 (3)^3^ DHA group 2 11 (2)^3^ Placebo group 13 (4)^3^	Biopsy	① DHA group 1: 250 mg/d (n = 20) ② DHA group 1: 500 mg/d (n = 20)	Placebo (n = 20)	A balanced lowcalorie diet and physical activity	6-mouths	24-mouths	0
Lavine et al (2011)	USA	Patients aged 8–17 years with NAFLD and persistently elevated levels of ALT	173 (140/33)	13.1 (2.4)	Biospy	① vitamin E+ metformin placebo: Vitamin E 400 IU, bid (n = 58) ② Metformin + vitamin E placebo: metformin 500 mg, bid (n = 57)	Vitamin E placebo and metformin placebo (n = 58)	Standardized recommendations for life-style modification (dietary, weight loss, and exercise)	24-mouths	30-mouths	23
Akcam et al (2011)	Turkey	Obese adolescents with NAFLD	67 (32/35)	12.3 (1.7)	Ultrasound	① Metformin: 850 mg/d (n = 22) ② Vitamin E: 400 IU, bid (n = 23)	The diet and exercise group (n = 22)	An individually tailored diet, exercise, and behavioral therapy	6-mouths	6-mouths	0

Legends: Values of age are given in mean and standard deviation (SD) unless otherwise stated.

1 Values given in median and interquartile range (IQR).

2 Values given in range.

3 Values given in median and IQR, difference.

4 Omega-3, fatty acids includes docosahexaenoic acid and eicosapentaenoic acid.

5 Omega-6, sunflower oil.

6 After 2 months, liver sonography was done for the patients. If no response was there, medicinal treatment would be continued for two more months and again sonography would be done by the same radiologist.

Abbreviations: M, Male; F, Female; NAFLD, Nonalcoholic fatty liver disease; NASH, Nonalcoholic steatohepatitis; rhGH, Recombinant human growth hormone; NA, Not available; NR, Not reported; PUFA, Polyunsaturated fatty acid; CBDR, Cysteamine bitartrate delayed release; Qd–Quaque die; Bid–Two times a day; Tid–Three times a day; NAS, NAFLD, activity score; DHA, Docosahexaenoic acid; ALT, Alanine aminotransferase; VSL#3 – a mixture of eight probiotic strains (*Streptococcus* thermophilus, bifidobacteria [B. breve, B. infantis, B. longum], *Lactobacillus* acidophilus, L. plantarum, L. paracasei, and L. delbrueckii subsp. bulgaricus).

Quality assessment indicated an overall low risk of bias. All studies were described as randomized groupings, but five studies (Akcam et al., 2011; Shiasi Arani et al., 2014; Boyraz et al., 2015; Namakin et al., 2021; Xue et al., 2022) did not describe the randomization process and one study (Famouri et al., 2017a) had a high risk of selection bias because it generated random sequences by parity. Allocation concealment was not described in seven studies (Akcam et al., 2011; Ghergherehchi et al., 2013; Shiasi Arani et al., 2014; Boyraz et al., 2015; Namakin et al., 2021; Saneian et al., 2021; Xue et al., 2022), and one study (Famouri et al., 2017a) adopted a non-concealment process that may have introduced selection bias. Three studies (Shiasi Arani et al., 2014; Boyraz et al., 2015; Xue et al., 2022) had potential missed visit bias that could have affected the reporting of the actual outcomes of the study. Regarding reporting bias, one study (Namakin et al., 2021) did not report the primary outcome, and, and for another, bias could not be determined due to unavailability of the protocol (Homaei et al., 2022). No other significant biases were identified. A detailed summary of the risk of bias assessment is provided in Supplementary File 3.

### 3.2 Primary outcome: an improvement in hepatic steatosis

#### 3.2.1 Direct meta-analysis

**FIGURE 3:**
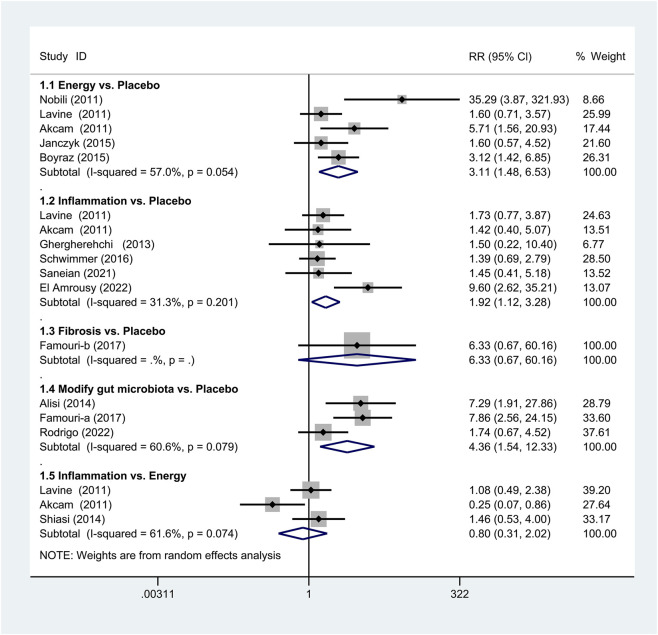
Direct meta-analysis of different pharmacological interventions for improving hepatic steatosis.

Compared to the placebo, treatments modulating energy (5 RCTs; RR: 3.11; 95% CrI: 1.48–6.53; *P* < 0.05), inflammation (6 RCTs; RR: 1.92; 95% CrI: 1.12–3.28; *P* < 0.05), and gut microbiota (3 RCTs; RR: 4.36; 95% CrI: 1.54–12.33; *P* < 0.05) was found to be more likely to improve hepatic steatosis in pediatric NAFLD patients. In head-to-head trials comparing different interventions, treatment modulating inflammation was comparable to treatment modulating energy in improving hepatic steatosis (3 RCTs; RR: 0.80; 95% CrI: 0.31–2.02; *P* > 0.05) (Figure 3).

#### 3.2.2 Network meta-analysis

**FIGURE 4:**
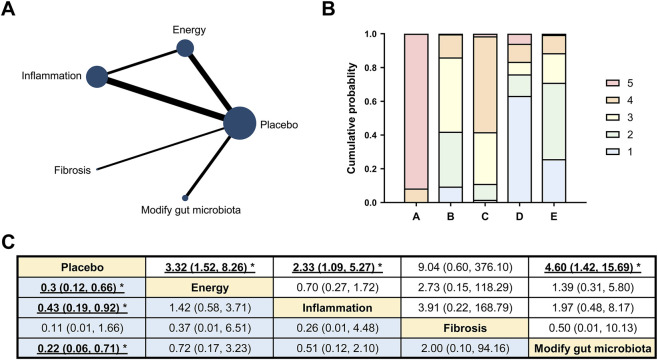
Bayesian network Meta-analysis results of different pharmacological interventions for improving hepatic steatosis. **(A)** Network evidence map (the size of the nodes and the thickness of the edges are weighted according to the number of patients and the number of studies evaluating each treatment respectively); **(B)** cumulative ranking probability plot (A ∼ E represent five different interventions, where A is placebo, B is an energy-modulating agent, C is an inflammation-modulating agent, D is a fibrosis-modulating agent, and E is a microecological agent); **(C)** league table for a two-by-two comparison of different pharmacological interventions (* represents a statistically significant difference, *P* < 0.05).

Overall, 14 studies (Akcam et al., 2011; Lavine et al., 2011; Nobili et al., 2011; Ghergherehchi et al., 2013; Alisi et al., 2014; Shiasi Arani et al., 2014; Boyraz et al., 2015; Janczyk et al., 2015; Schwimmer et al., 2016; Famouri et al., 2017a; Famouri et al., 2017b; Saneian et al., 2021; El Amrousy et al., 2022; Rodrigo et al., 2022) reported improvements in hepatic steatosis with pharmacological interventions of different mechanisms/pathways. Figure 4A shows the network evidence map for the primary outcome of this study. The NMA results indicated that treatment-modulating fibrosis was most likely to improve hepatic steatosis in pediatric NAFLD patients (SUCRA: 79.18%), followed by treatment-modulating gut microbiota (SUCRA: 71.15%), energy (SUCRA: 59.29%) and inflammation (SUCRA: 38.21%), with placebo being the least effective (SUCRA: 2.17%) (Figure 4B, Supplementary File 4; Supplementary Tables S1, S2). In pairwise comparisons within the network, treatment modulating energy (RR: 3.32; 95% CrI: 1.52–8.26; *P* < 0.05), inflammation (RR: 2.33; 95% CrI: 1.09–5.27; *P* < 0.05) and gut microbiota (RR: 4.60; 95% CrI: 1.42–15.69; *P* < 0.05) were all more effective than placebo in improving hepatic steatosis, while the efficacy of treatment modulating fibrosis (RR: 9.04; 95% CrI: 0.60–15.69; *P* > 0.05) was comparable to placebo (Figure 4C, Supplementary File 4; Supplementary Figure S1).

### 3.3 Secondary outcomes

#### 3.3.1 Reduction in liver enzymes (ALT, AST)

##### 3.3.1.1 Direct meta-analysis

Compared to placebo, treatment-modulating energy (7 RCTs; WMD: 9.495; 95% CrI: 13.823 − −5.168; *P* < 0.05), inflammation (6 RCTs; WMD: 10.553; 95% CrI: 18.116 − −2.990; *P* < 0.05), and fibrosis (2 RCTs; WMD: 3.602; 95% CrI: 7.193 − −0.012; *P* < 0.05) significantly reduced ALT levels. Treatment-modulating energy (5 RCTs; WMD: 3.150; 95% CrI: 5.627 − −0.672; *P* < 0.05) and inflammation (6 RCTs; WMD: 5.725; 95% CrI: 10.796 − −0.654; *P* < 0.05) also reduced AST levels (Forest plots for direct comparison as shown in Supplementary File 5; Supplementary Figures S1, S2).

##### 3.3.1.2 Network meta-analysis

**FIGURE 5:**
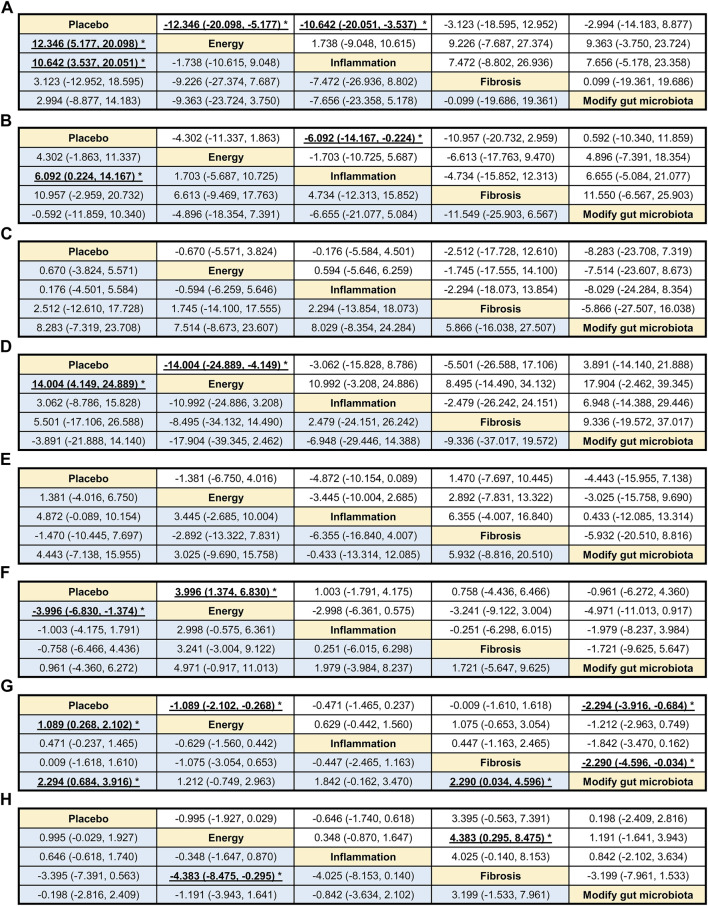
League table for a two-by-two comparison of different pharmacological interventions for improving secondary outcomes. **(A)** ALT, **(B)** AST, **(C)** TC, **(D)** TG, **(E)** LDL-C, **(F)** HDL-C, **(G)** BMI, **(H)** HOMA-IR. * represents a statistically significant difference, *P* < 0.05.

**FIGURE 6:**
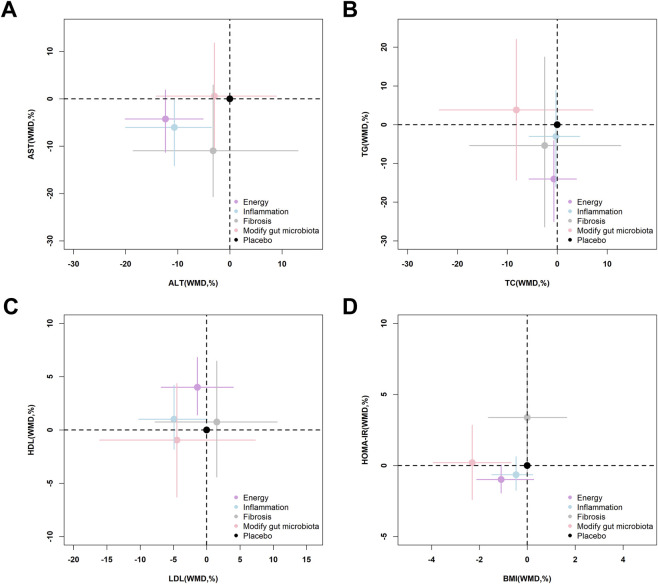
A plot of two-dimensional comparison of different mechanisms/pathways of pharmacological interventions to improve secondary outcomes. **(A)** ALT vs. AST, **(B)** TC vs. TG, **(C)** LDL-C vs. HDL-C, **(D)** BMI vs. HOMA-IR.

All active interventions were superior to placebo in reducing ALT levels. Energy-modulating (WMD: 12.346; 95% CrI: 20.098 − −5.177; *P* < 0.05) and inflammation-modulating treatments (WMD: 10.642; 95% CrI: 20.051 − −3.537; *P* < 0.05) showed significant effects (Supplementary File 5). In terms of reducing ALT levels, energy-modulating treatment had the highest probability of being the most effective (SUCRA: 86.20%), followed by inflammation (SUCRA: 76.49%), fibrosis (36.98%), gut microbiota (34.74%), and placebo (15.59%). For AST reduction, only inflammation-modulating treatment was significantly superior to placebo (WMD: 6.092; 95% CrI: 14.167 − −0.224; *P* < 0.05) (Figures 5A,B). Treatments modulating fibrosis (SUCRA: 85.72%), inflammation (SUCRA: 70.07%), energy (SUCRA: 55.75%), gut microbiota (SUCRA: 21.17%), and placebo (SUCRA: 17.29%) were found to be in decreasing order of treatment ranking. A two-dimensional ranking of combined efficacy for reducing both ALT and AST is presented in Figure 6A, with treatment to modulate energy and inflammation dominating the ranking of combined efficacy in reducing liver enzyme levels compared to placebo (null points in the graph), followed by fibrosis, with treatment to modulate gut microbiota having the poorest combined efficacy.

#### 3.3.2 Improvement in lipid profiles (TC, TG, LDL-C, HDL-C)

##### 3.3.2.1 Direct meta-analysis

Compared to placebo, both treatments modulating energy (8 RCTs; WMD: 5.675; 95% CrI: 11.268 − −0.082; *P* < 0.05) and fibrosis (2 RCTs; WMD: 8.336; 95% CrI: 15.158 − −1.513; *P* < 0.05) significantly reduced TG levels. In addition, treatment-modulating energy improved LDL-C (5 RCTs; WMD: 0.434; 95% CrI: 0.789 − −0.080; *P* < 0.05) and HDL-C levels (6 RCTs; WMD: 1.879; 95% CrI: 0.057–3.700; *P* < 0.05). Head-to-head comparisons suggested that treatment-modulating inflammation might worsen the lipid profile compared to energy, as it might increase TG levels (3 RCTs; WMD: 20.183; 95% CrI: 1.946–38.420; *P* < 0.05) and decrease blood “good cholesterol” HDL-C levels (2 RCTs; WMD: 2.255; 95% CrI: 4.271 − −0.240; *P* < 0.05). No active intervention significantly reduced TC levels (Forest plots for direct comparison as shown in Supplementary File 6; Supplementary Figures S1-S4).

##### 3.3.2.2 Network meta-analysis

Treatment modulating energy was significantly superior to placebo in reducing TG contents (WMD: 14.004; 95% CrI: 24.889 − −4.149; *P* < 0.05) and increasing HDL-C levels (WMD: 3.996; 95% CrI: 1.374–6.830; *P* < 0.05) (Figures 5C–F, Supplementary File 1). The results of the two-dimensional comparison of interventions to improve lipid profiles are shown in Figures 6B,C. No active treatment simultaneously reduced both TC and TG levels. However, energy- and inflammation-modulating interventions showed superior combined efficacy in reducing LDL-C while increasing HDL-C.

#### 3.3.3 Improvement in metabolic indicators (BMI, HOMA-IR)

##### 3.3.3.1 Direct meta-analysis

Compared to placebo, treatment modulating energy (5 RCTs; WMD: 1.071; 95% CrI: 1.876 − −0.266; *P* < 0.05) and gut microbiota (1 RCT; WMD: 2.300; 95% CrI: 2.899 − −1.701; *P* < 0.05) significantly reduced BMI values. For improving insulin resistance (HOMA-IR), treatments modulating inflammation (5 RCTs; WMD: 1.096; 95% CrI: 1.774 − −0.418; *P* < 0.05) were effective, whereas treatments modulating fibrosis (1 RCT; WMD: 3.400; 95% CrI: 0.130–6.670; *P* < 0.05) appeared to worsen it. Head-to-head comparisons indicated that treatment-modulating inflammation as associated with higher BMI compared to treatment-modulating energy (3 RCTs; WMD: 0.719; 95% CrI: 0.265–1.173; *P* < 0.05) (Supplementary File 7; Supplementary Figures S1, S2).

##### 3.3.3.2 Network meta-analysis

Compared to placebo, treatments modulating energy (WMD: 1.089; 95% CrI: 2.102 − −0.268; *P* < 0.05) and gut microbiota (WMD: 2.294; 95% CrI: 3.916 − −0.684; *P* < 0.05) significantly lowered BMI values. Further pairwise comparisons of the different interventions indicated that treatment-modulating gut microbiota was more effective than fibrosis in reducing BMI values (WMD: 2.290; 95% CrI: 4.596 − −0.034; *P* < 0.05). For HOMA-IR, no active intervention outperformed placebo. However, fibrosis-modulating treatment potentially exacerbated insulin resistance compared to energy-modulating treatment (WMD: 4.383; 95% CrI: 0.295–8.475; *P* < 0.05) (Figures 5G,H, Supplementary File 7). A two-dimensional comparison of pharmacological interventions by different mechanisms/pathways to improve metabolic indicators is shown in Figure 6D.

The results of the direct meta-analysis and network meta-analysis for the primary and secondary outcomes of this study are summarised in Supplementary File 8; Supplementary Tables S1-S4.

### 3.4 Publication bias and network coherence

The distribution of potential effect modifiers across the four mechanistic pathway nodes was assessed to evaluate the transitivity assumption (Supplementary File 9). Baseline characteristics were broadly comparable across the energy, inflammation, fibrosis, and gut microbiota modulator categories. The weighted mean age, sex distribution, weighted mean BMI (or BMI SDS), treatment/evaluation period, and follow-up duration of participants were similar across categories. Lifestyle modification (dietary and exercise counseling) was recommended as a background intervention in all included trials. The proportion of patients with NAFLD diagnosed by ultrasound or liver biopsy varied across studies, but no systematic differences in baseline steatosis grade were observed between pathway nodes, indicating comparable baseline disease severity. These findings support the plausibility of the transitivity assumption, as there were no apparent imbalances in effect modifiers that would bias the indirect comparisons.

Due to the limited number of studies available for each direct comparison, a formal assessment of publication bias was not feasible. Network plots for all outcomes contained closed loops, and the inconsistency tests indicated that there was no significant difference (inconsistency) between the direct and indirect estimates (Supplementary File 10; Supplementary Figures S1, S2). The deviance information criterion (DIC) was utilized to compare the degree of fit between the consistent and inconsistent models. The difference in DIC values between consistency and inconsistency models was less than 5, supporting the assumption of consistency in this network (Supplementary File 10; Supplementary Table S1). Furthermore, comparison between fixed- and random-effects models confirmed the random-effects model as the more appropriate choice for this NMA (Supplementary File 10; Supplementary Table S2). The absence of significant inconsistency across the network is particularly important given that interventions were grouped into broad mechanistic pathway nodes. The consistency results suggest that direct and indirect evidence were in agreement across the network, which supports the validity of the pathway-level comparisons. Importantly, the consistency findings align with the balanced distribution of key effect modifiers (e.g., baseline disease severity, diagnostic modality, age, BMI, background lifestyle interventions) across the four pathway nodes. This balance strengthens the credibility of the transitivity assumption and indicates that the observed consistency is not merely an artifact of the grouping strategy but reflects genuine coherence within the evidence base.

### 3.5 Sensitivity analysis

A sensitivity analysis excluding five studies judged to be at high risk of bias (Shiasi Arani et al., 2014; Boyraz et al., 2015; Famouri et al., 2017a; Namakin et al., 2021; Xue et al., 2022) yielded results consistent with the primary analysis, supporting the robustness of the findings (Supplementary File 11). After excluding agents with ambiguous or overlapping mechanisms (L-carnitine, silymarin), the pooled effect estimates remained largely unchanged **(** Supplementary File 12). Following the reclassification of multi-target agents (L-carnitine, silymarin) from the inflammation pathway into the energy pathway, the recalculated effect estimates for energy modulators remained statistically significant, with 95% CrIs overlapping substantially with those of the primary analysis (Supplementary File 13). These sensitivity analyses collectively demonstrated that the primary findings were robust to variations in pathway classification and agent selection.

To further assess the robustness of the primary findings against heterogeneity in outcome assessment, we conducted a stratified NMA by primary outcome assessment modality. In the ultrasound-based subgroup (12 studies), energy modulators remained significantly superior to placebo in improving hepatic steatosis (RR: 2.61; 95% CrI: 1.04–7.09; *P* < 0.05). In the histology-based subgroup (7 studies), the effect estimate for energy modulators was directionally consistent but had a wider credible interval (RR: 6.30; 95% CrI: 0.47–123.71; *P* > 0.05), reflecting lower statistical power due to the smaller number of biopsy-diagnosed trials. Overall, the findings across assessment modalities were generally consistent, and no systematic bias was observed (Supplementary File 14). Additionally, a sensitivity analysis restricted to trials with a follow-up duration of at least 24 weeks yielded results consistent with the primary analysis, with no material changes to the pooled effect estimates (Supplementary File 15). To evaluate the impact of the assumed correlation coefficient of 0.5 used to estimate change-score SDs, we conducted a sensitivity analysis with alternative correlation coefficients of 0.3 and 0.7. The pooled effect estimates for all continuous outcomes remained consistent across the three correlation values, with overlapping 95% CrIs (Supplementary File 16). Collectively, these sensitivity analyses demonstrated that the primary findings were robust to variations in pathway classification, agent selection, outcome assessment modality, follow-up duration, and the assumed correlation coefficients.

### 3.6 Safety assessment

A structured overview of AEs reported in the included trials is provided in Supplementary File 17. Among the 19 RCTs, only six reported AEs in sufficient detail (Akcam et al., 2011; Lavine et al., 2011; Janczyk et al., 2015; Schwimmer et al., 2016; Vos et al., 2022; Xue et al., 2022). For energy-modulating agents, rhGH (Xue et al., 2022) was associated with fasting blood glucose >6 mmol/L (6/22) and temporary hypothyroidism (2/22). Omega-3 (Janczyk et al., 2015) was associated with mild abdominal discomfort (1/30). Metformin was reported in two studies (Lavine et al., 2011; Akcam et al., 2011) to be associated with new-onset diabetes (1/57), elevations in liver enzymes (ALT >2 × baseline: 9/57; AST >2 × baseline: 9/57), diarrhea and mild abdominal pain (1/22), and mild abdominal discomfort (1/22). No metformin-related serious AEs (e.g., vomiting or lactic acidosis) were observed, and no patient discontinued due to AEs. For inflammation modulators, Vitamin E was associated with hypoglycemia (1/58) and liver enzyme elevations (ALT >2 × baseline: 1/58; AST >2 × baseline: 4/58) (Lavine et al., 2011). CBDR (cysteamine bitartrate delayed-release) (Schwimmer et al., 2016) was associated with a wide range of AEs across multiple organ systems, as detailed in Supplementary File 17. For fibrosis modulators, losartan (Vos et al., 2022) was associated with moderate AEs, with no significant difference in AE frequency between losartan and placebo groups (5/33 vs. 10/34, *P* = 0.14). No AEs were reported for gut microbiota modulators in the included RCTs. Serious adverse events were infrequently reported, and no study reported discontinuations directly attributable to AEs. The limited and heterogeneous reporting precluded formal meta-analysis of safety outcomes.

## 4 Discussion

With the rising prevalence of obesity among children, pediatric NAFLD has emerged as a major public health issue and a leading cause of various liver-related comorbidities (Draijer et al., 2019; Suri et al., 2021). While lifestyle modification remains the cornerstone of management, the efficacy of pharmacologic interventions is not well defined. The present NMA provides evidence for the effectiveness of treatment modulating energy, inflammation, and gut microbiota in reducing hepatic steatosis in pediatric NAFLD patients. To our knowledge, this is the first NMA to compare the effects of different mechanistic pathways of drugs in this population.

The pathogenesis of NAFLD has evolved from the classic “two-hit” hypothesis (Day and James, 1998) to the contemporary “multiple-hit” model (Fang et al., 2018), which incorporates the roles of endoplasmic reticulum stress, gut-liver axis dysfunction, adipokine imbalance, and hepatic stellate cell activation (Clemente et al., 2016). As an imbalance of lipid metabolism is the driving factor of NAFLD, the majority of pediatric NAFLD patients are in the stage of simple hepatic steatosis without any clinical manifestations, and only a small proportion progresses to NASH (Ng et al., 2022). Consequently, unlike NMAs in adults which often prioritize fibrosis regression (Nobili et al., 2012; Majzoub et al., 2021; Ng et al., 2022), the present NMA places emphasis on improvement in hepatic steatosis (at least 1-grade reduction in the sonographic grade of fatty liver without worsening of fibrosis) as the primary outcome. This endpoint aligns with the early disease stage and natural history typical of pediatric NAFLD. The natural course of NAFLD is highly dynamic, with metabolic syndrome, type 2 diabetes mellitus, hypertension, dyslipidemia, and central obesity being common risk factors (Patton et al., 2010; Della Corte et al., 2014), which also predispose children to severe steatosis and advanced fibrosis (Patton et al., 2010; Suri et al., 2021). Liver enzyme levels, particularly ALT, reflect disease activity and are markers of improvement (Seko et al., 2015). Patients with lower baseline AST levels are more likely to experience spontaneous remission of NASH (Kleiner et al., 2019). Given that NAFLD is considered the hepatic manifestation of metabolic syndrome (Grundy et al., 2005; Loomba et al., 2012), and considering the strong associations with dyslipidemia (Youssefian et al., 2019) and obesity (Lee et al., 2024), secondary outcomes included changes in liver enzymes, lipid profiles, and metabolic parameters (BMI, HOMA-IR). These measures represent key risk factors and prognostic indicators in pediatric NAFLD, allowing for a comprehensive comparison of treatment pathways.

In this study, docosahexaenoic acid (DHA), metformin, omega-3, polyunsaturated fatty acids (PUFA), and recombinant human growth hormone (rhGH) were categorized as energy subsets for data synthesis analysis. Their therapeutic mechanisms in NAFLD primarily involve regulating hepatocyte lipid metabolism and improving insulin sensitivity. Studies by Nobili et al. (2011), Nobili et al. (2013) have shown that DHA supplementation attenuated hepatic steatosis, reduced serum ALT and TG levels, and improved insulin sensitivity in pediatric NAFLD patients. PUFAs, especially eicosapentaenoic acid (EPA, C20:5n3), play a regulatory role in crucial pathways of hepatic lipid metabolism through the activation of transcription factors (PPARα, PPARγ, SREBP-1, ChREBP) (Marx et al., 2004; Jump, 2008), promotion of fatty acid β-oxidation and improvement of insulin sensitivity (Stienstra et al., 2007; Tetri et al., 2008). Metformin exerts multiple beneficial effects, including weight reduction, decreased transaminase levels, improved liver histology, and enhanced insulin sensitivity (Angelico et al., 2007; Nobili et al., 2008; Nar and Gedik, 2009). Furthermore, dysregulation of the GH-IGF-1 axis has been implicated in NAFLD progression (Sirbu et al., 2013; Cianfarani et al., 2014). A recent clinical trial based on a small sample confirmed that rhGH treatment improves BMI and lipid metabolism in obese children (Gilliland et al., 2016), without affecting glucose metabolism (Liang et al., 2018). The present NMA results indicate that treatment-modulating energy is superior to placebo in improving hepatic steatosis. Within the comparative ranking of different mechanistic pathways, energy-modulating agents were also the most effective in reducing ALT and TG, increasing HDL-C, and improving HOMA-IR. Notably, a previous NMA in adult NASH patients similarly identified energy-modulating agents as the most likely to achieve histological improvement (Ng et al., 2022). A plausible explanation for the superior and broad-spectrum efficacy of energy-modulating agents in pediatric NAFLD lies in the disease’s natural history. Unlike adults, who more frequently present with advanced fibrosis or NASH, most children with NAFLD exhibit simple hepatic steatosis without significant inflammation or fibrosis. At this early stage, the primary pathophysiological driver is excess energy delivery to hepatocytes, leading to lipid accumulation, rather than downstream inflammatory or fibrotic cascades. Therefore, interventions that directly target energy metabolism (by improving insulin sensitivity, suppressing *de novo* lipogenesis, or enhancing mitochondrial β-oxidation) may address the root cause of the disease in children. In contrast, anti-inflammatory or anti-fibrotic therapies may be more relevant in later stages of disease progression. This stage-specific mechanism likely underlies the consistent and comprehensive benefits of energy modulators across hepatic steatosis, liver enzymes, lipid profiles, and insulin resistance observed in our pediatric population. These findings highlight the relative potential of energy-targeting interventions in pediatric NAFLD, but they should be interpreted as probabilistic signals rather than definitive evidence of superiority. Given the wide credible intervals for some comparisons and the limited number of studies, the observed rankings provide a prioritization signal for future head-to-head trials rather than a basis for clinical recommendation. Further rigorous preclinical studies and large-scale multicenter RCTs are warranted to confirm these findings and evaluate long-term effectiveness and safety.

Despite the incomplete understanding of NAFLD’s pathogenesis, there is a consensus on NAFLD as a multi-pathogenic disease with an interaction of genetic, epigenetic, and environmental factors. This multifactorial pathogenesis should be taken into account when designing appropriate therapeutic interventions and predicting treatment response. From this perspective, multi-targeted therapies addressing the key clinical and molecular features of NAFLD represent a promising approach for disease management and prevention of progression. Beyond disturbances in energy metabolism, chronic, non-infectious inflammation is a central pathological driver in NAFLD. Our NMA indicates that anti-inflammatory therapies are superior to placebo in improving hepatic steatosis and significantly reducing ALT levels, though their effects on lipid profiles and metabolic parameters were comparable to placebo. Microecological agents and the gut-liver axis are emerging hotspots in NAFLD research fields. Probiotics may confer benefit by enhancing epithelial barrier function and reducing intestinal permeability and endotoxemia (Milosevic et al., 2019; Chen and Vitetta, 2020; Hu et al., 2020; Rodríguez-Pastén et al., 2023). In our analysis, treatments modulating gut microbiota showed a distinct advantage in reducing BMI (SUCRA = 96.25%). In contrast, evidence for anti-fibrotic agents remains inconsistent. While ranking probabilities suggested potential benefit, direct and indirect comparisons did not demonstrate superiority over placebo in improving steatosis or reducing AST levels. These results should be interpreted with caution given the limited and conflicting data. Promising initial findings have emerged for silymarin, silymarin treatment (at a dosage of 5 mg/kg/d) for a duration of 12 weeks led to improvements in hepatic fatty infiltration and liver function in pediatric patients with NAFLD aged 5–16 years (Famouri et al., 2017a). However, although preliminary studies reported supportive data for losartan in animal models (Rosselli et al., 2009), a multicenter RCT in histologically confirmed children with NAFLD found no improvement in ALT, AST, or GGT levels after 24 weeks of losartan treatment (Vos et al., 2022).

A key strength of this analysis is its comprehensive evaluation of the relative efficacy of drugs targeting distinct mechanistic pathways in pediatric NAFLD, offering insights into critical disease pathways. In the absence of sufficient head-to-head trials, NMA provides valuable comparative evidence where direct pooled analyses are limited. The consistency between direct and indirect comparisons in our NMA supports the credibility of the findings. The robustness of our primary findings was further supported by additional sensitivity analyses addressing potential sources of heterogeneity. Results from the series of sensitivity analyses indicated that excluding multi-target agents or reclassifying them, variations in follow-up duration, and assumptions regarding correlation coefficients did not materially alter the main conclusions, further reinforcing the reliability of our pathway-level comparisons. The credibility of the NMA findings is supported by careful attention to the core assumptions of transitivity and consistency. The distribution of potential effect modifiers was broadly balanced across the four mechanistic pathway nodes, supporting the plausibility of transitivity. Furthermore, node-splitting analyses and DIC comparisons revealed no significant inconsistency between direct and indirect evidence across the network. These methodological safeguards reinforce the validity of the comparative efficacy estimates and suggest that the grouping of interventions into pathway nodes did not introduce spurious findings.

Several limitations warrant consideration. First, the classification of interventions, based on established pathogenic mechanisms (Gerges et al., 2021), may be debated for multi-targeted agents, despite expert consensus (Muthiah and Sanyal, 2020; Ng et al., 2022). The robustness of our pathway-based classification was further supported by two pre-specified sensitivity analyses. Excluding agents with ambiguous or overlapping mechanisms yielded pooled effect estimates consistent with the primary analysis, with overlapping 95% CrIs for all primary and secondary outcomes. Similarly, reclassifying multi-target agents did not materially alter the comparative effect estimates. These sensitivity analyses suggest that the primary findings are not artifacts of the grouping strategy and that the conclusions regarding the relative advantages of energy-modulating agents remain reliable. The classification of interventions into four discrete mechanistic pathways, while necessary for the network structure, represents a simplification of the complex pharmacology of several agents. Our pre-specified hierarchical rule assigned each agent to its primary mechanism based on pediatric NAFLD-specific evidence, but we acknowledge that this simplified model cannot fully account for the pleiotropic effects of multi-target drugs. Consequently, the relative efficacy estimates for a given pathway may partly reflect contributions from secondary mechanisms. Nevertheless, the simplified pathway model cannot fully account for the pleiotropic effects of multi-target drugs, and the efficacy estimates for a given pathway may partly reflect contributions from secondary mechanisms. Therefore, these results should be interpreted as hypothesis-generating, offering valuable insights for future drug development and combination regimen selection rather than definitive clinical recommendations. Second, treatment durations varied widely (2–24 months), potentially affecting endpoint reliability. Third, inherent design limitations (such as individualized lifestyle prescriptions and unmeasured adherence) may confound the assessment of pharmacological effects. Fourth, ranking probabilities should be interpreted cautiously, as they are influenced by network structure and the smaller number of studies in the fibrosis-modulating category. Fifth, heterogeneity in the assessment of the primary outcome (ultrasound vs. liver biopsy) represents another limitation. Although our sensitivity analysis stratified by assessment modality showed generally consistent results, the biopsy-based subgroup included only seven studies, leading to wider credible intervals and lower statistical power. This difference may affect the precision of estimates, but it does not appear to systematically bias the direction of effects. Future pediatric NAFLD trials should consider standardizing outcome assessment or, when feasible, using histology as the reference standard to reduce modality-driven variability. Finally, the safety profile of interventions could not be comprehensively analyzed due to inconsistent reporting of adverse events, which is a crucial factor in clinical decision-making. This gap is particularly important given that the ultimate clinical utility of any pharmacological intervention depends not only on efficacy but also on its safety and tolerability, especially in the pediatric population. Therefore, while our findings suggest relative advantages of energy-modulating agents in improving steatosis and metabolic parameters, these conclusions must be interpreted with caution in the absence of robust comparative safety data. Future pediatric NAFLD trials should adopt standardized safety reporting frameworks to enable more comprehensive safety assessments and facilitate meaningful comparisons across interventions.

## 5 Conclusion

In this study, we performed an NMA to assess the effectiveness of drugs targeting different mechanistic pathways for treating pediatric NAFLD patients. Among the available pharmacological targets, we revealed the relative advantages of drugs that modulate energy, inflammation, and gut microbiota pathways in improving hepatic steatosis. Further analysis of secondary outcomes indicated that treatments modulating energy had the highest probability of being among the most effective for reducing ALT levels, lowering TG contents, increasing HDL-C levels, and improving insulin resistance. Treatments modulating gut microbiota had the highest probability of being among the most effective for reducing BMI. These findings support prioritizing core metabolic regulatory targets (e.g., PPAR agonists, FXR agonists) in future pediatric drug development and provide a preliminary theoretical basis for combination strategies pairing energy modulators with gut microbiota modulators. They also highlight that effective management of pediatric NAFLD may require addressing both hepatic pathology and systemic metabolic/axis homeostasis. Further well-designed head-to-head trials are needed before clinical recommendations can be made.

## Data Availability

The original contributions presented in the study are included in the article/Supplementary Material, further inquiries can be directed to the corresponding author.
