# Supplementary material for: Comparative efficacy of drugs with different mechanistic pathways/targets in the treatment of pediatric NAFLD: evidence from a Bayesian network meta-analysis
Source: Front Pharmacol. 2026 May 25;17:1777515. doi: 10.3389/fphar.2026.1777515 (PMC13243036; doi:10.3389/fphar.2026.1777515)
Supplement: Supplementary file 1 [file DataSheet1.pdf]

Contents

- ***Supplementary File 1. Actual Search Strategies***..........3
    - √ **Table S1-A.** Final search strategy for Ovid MEDLINE..........3
    - √ **Table S1-B.** Final search strategy for EMBASE..........5
    - √ **Table S1-C.** Final search strategy for Cochrane Central..........7
    - √ **Table S1-D.** Final search strategy for PUBMED..........9
    - √ **Table S1-E.** Final search strategy for Web of Science..........12
    - √ **Table S1-F.** Final search strategy for Scopus..........15
- ***Supplementary File 2. Summary of therapeutic categories***..........17
- ***Supplementary File 3. Risk of bias assessment***..........21
- ***Supplementary File 4. Results of NMA of primary outcome***..........23
- ***Supplementary File 5. Results of NMA of liver enzymes (ALT, AST)***..........25
- ***Supplementary File 6. Results of NMA of lipid profile (TC, TG, HDL-C, LDL-C)***..........32
- ***Supplementary File 7. Results of NMA of metabolic parameters (BMI, HOMA-IR)***..........44
- ***Supplementary File 8. Pooled summary estimates from direct and network meta-analysis***..........52
- ***Supplementary File 9. Distribution of potential effect modifiers across mechanistic pathway nodes***..........57
- ***Supplementary File 10. Node-splitting analysis of inconsistency***..........58
- ***Supplementary File 11. Sensitivity analysis after excluding trials with a high risk bias***..........61
- ***Supplementary File 12. Sensitivity analysis after excluding trials involving multi-target agents***..........64
- ***Supplementary File 13. Sensitivity analysis after reclassification of multi-target agents***..........67
- ***Supplementary File 14. Sensitivity analysis of NAFLD diagnostic methods***..........70

- ***Supplementary File 15. Sensitivity analysis by follow-up duration***..73
- ***Supplementary File 16. Sensitivity analysis for the assumption of correlation coefficients of continuous variables***..76
- ***Supplementary File 17. Safety profile of included interventions by mechanistic pathway***.......79

Supplementary File 1. Actual Search Strategies

Table S1-A. Final search strategy for Ovid MEDLINE

Database: OVID Medline Epub Ahead of Print, In-Process & Other Non-Indexed Citations, Ovid MEDLINE(R) Daily and Ovid MEDLINE(R) 1946 to Present

#	Query	Results
1	exp fatty liver/	43,871
2	exp Non-alcoholic Fatty Liver Disease/	21,309
3	((("non-alcoholic" or nonalcoholic) and ("fatty liver*" or steatohepatit* or "liver steatos*" or "hepatic steatos*" or "visceral steatos*" or "steatohepatitis" or "steatosis")) or ("Nonalcoholic Steatohepatitis" or NAFLD or NASH or non-AFLD or non-ASH)).mp.	41,335
4	1 or 2 or 3	58,719
5	exp child/	2,116,179
6	Infant/	858,697
7	Adolescent/	2,205,925
8	(child$ or adolescent$ or infant$ or juvenil$ or pediatric$ or paediatric$ or " young person$" or "young people" or youth$ or "young adult$").ab,kf,ti.	2,477,780
9	5 or 6 or 7 or 8	4,405,836
10	4 and 9	6,766
11	randomized controlled trial.pt.	582,703
12	controlled clinical trial.pt.	95,139
13	"random*".ab,ti.	1,373,083
14	"placebo*".ab,ti.	242,176
15	"single blind*".ab,ti.	22,225
16	"double blind*".ab,ti.	167,398
17	"triple blind*".ab,ti.	1,367
18	exp "Clinical Trials as Topic"/	380,198
19	exp drug therapy/	1,490,977
20	groups.ab,ti.	2,475,355
21	trial.ab,ti.	729,329
22	13 or 14 or 15 or 16 or 17 or 18 or 19 or 20 or 21	5,078,663
23	11 or 12 or 22	5,165,666
24	exp animals/ not humans.sh.	5,086,122
25	(alpaca or alpacas or amphibian or amphibians or animal or animals or antelope or armadillo or armadillos or avian or baboon or baboons or beagle or beagles or bee or bees or bird or birds or bison or bovine or buffalo or buffaloes or buffalos or "c elegans" or "Caenorhabditis elegans" or camel or camels or canine or canines or carp or cats or cattle or chick or chicken or chickens or chicks or chimp or chimpanze or chimpanzees or chimps or cow or cows or "D	10,667,714

	melanogaster" or "dairy calf" or "dairy calves" or deer or dog or dogs or donkey or donkeys or drosophila or "Drosophila melanogaster" or duck or duckling or ducklings or ducks or equid or equids or equine or equines or feline or felines or ferret or ferrets or finch or finches or fish or flatworm or flatworms or fox or foxes or frog or frogs or "fruit flies" or "fruit fly" or "G mellonella" or "Galleria mellonella" or geese or gerbil or gerbils or goat or goats or goose or gorilla or gorillas or hamster or hamsters or hare or hares or heifer or heifers or horse or horses or insect or insects or jellyfish or kangaroo or kangaroos or kitten or kittens or lagomorph or lagomorphs or lamb or lambs or llama or llamas or macaque or macaques or macaw or macaws or marmoset or marmosets or mice or minipig or minipigs or mink or minks or monkey or monkeys or mouse or mule or mules or nematode or nematodes or octopus or octopuses or orangutan or "orang-utan" or orangutans or "orang-utans" or oxen or parrot or parrots or pig or pigeon or pigeons or piglet or piglets or pigs or porcine or primate or primates or quail or rabbit or rabbits or rat or rats or reptile or reptiles or rodent or rodents or ruminant or ruminants or salmon or sheep or shrimp or slug or slugs or swine or tamarin or tamarins or toad or toads or trout or urchin or urchins or vole or voles or waxworm or waxworms or worm or worms or xenopus or "zebra fish" or zebrafish or cell).ab,hw,kw,ti.	
26	24 or 25	10,674,437
27	10 and 23	1,266
28	27 not 26	1,084
29	(congress* or letter or comment or editorial or published erratum or review or Meta-Analysis or Systematic Review).pt.	5,520,850
30	"clinical protocols".sh.	29,855
31	protocol.tw.	397,966
32	29 or 30 or 31	5,895,233
33	28 not 32	920
34	english.lg.	30,268,960
35	33 and 34	846

Table S1-B. Final search strategy for EMBASE

#1. 'fatty liver'/exp

#2. 'non-alcoholic fatty liver disease'/exp

#3. ('non-alcoholic':ti,ab,kw OR nonalcoholic:ti,ab,kw) AND ('fatty liver*':ti,ab,kw OR steatohepatit*:ti,ab,kw OR 'liver steatos*':ti,ab,kw OR 'hepatic steatos*':ti,ab,kw OR 'visceral steatos*':ti,ab,kw OR 'steatohepatitis':ti,ab,kw OR 'steatosis of liver':ti,ab,kw) OR 'nonalcoholic steatohepatitis':ti,ab,kw OR nafld:ti,ab,kw OR nash:ti,ab,kw OR 'non afld':ti,ab,kw OR 'non ash':ti,ab,kw

#4. #1 OR #2 OR #3

#5. 'child'/exp

#6. adolescent

#7. 'infant'/exp OR infant

#8. 'child*':ti,ab,kw OR 'adolescent*':ti,ab,kw OR 'infant*':ti,ab,kw OR 'juvenil*':ti,ab,kw OR 'pediatric*':ti,ab,kw OR 'paediatric*':ti,ab,kw OR 'young person*':ti,ab,kw OR 'young people':ti,ab,kw OR 'youth*':ti,ab,kw OR 'young adult*':ti,ab,kw

#9. #5 OR #6 OR #7 OR #8

#10. #4 AND #9

#11. 'crossover procedure':de OR 'double-blind procedure':de OR 'randomized controlled trial':de OR 'single-blind procedure':de OR random*:de,ab,ti OR factorial*:de,ab,ti OR crossover*:de,ab,ti OR ((cross NEXT/1 over*):de,ab,ti) OR placebo*:de,ab,ti OR ((doubl* NEAR/1 blind*):de,ab,ti) OR ((singl* NEAR/1 blind*):de,ab,ti) OR assign*:de,ab,ti OR allocat*:de,ab,ti OR volunteer*:de,ab,ti

#12. (animal OR nonhuman) NOT human

#13. (alpaca:ti,ab,kw OR alpacas:ti,ab,kw OR amphibian:ti,ab,kw OR amphibians:ti,ab,kw OR animal:ti,ab,kw OR animals:ti,ab,kw OR antelope:ti,ab,kw OR armadillo:ti,ab,kw OR armadillos:ti,ab,kw OR avian:ti,ab,kw OR baboon:ti,ab,kw OR baboons:ti,ab,kw OR beagle:ti,ab,kw OR beagles:ti,ab,kw OR bee:ti,ab,kw OR bees:ti,ab,kw OR bird:ti,ab,kw OR birds:ti,ab,kw OR bison:ti,ab,kw OR bovine:ti,ab,kw OR buffalo:ti,ab,kw OR buffaloes:ti,ab,kw OR buffalos:ti,ab,kw OR 'c elegans':ti,ab,kw OR 'caenorhabditis elegans':ti,ab,kw OR camel:ti,ab,kw OR camels:ti,ab,kw OR canine:ti,ab,kw OR canines:ti,ab,kw OR carp:ti,ab,kw OR cats:ti,ab,kw OR cattle:ti,ab,kw OR chick:ti,ab,kw OR chicken:ti,ab,kw OR chickens:ti,ab,kw OR chicks:ti,ab,kw OR chimp:ti,ab,kw OR chimpanze:ti,ab,kw OR chimpanzees:ti,ab,kw OR chimps:ti,ab,kw OR cow:ti,ab,kw OR cows:ti,ab,kw OR 'd melanogaster':ti,ab,kw OR 'dairy calf':ti,ab,kw OR 'dairy calves':ti,ab,kw OR deer:ti,ab,kw OR dog:ti,ab,kw OR dogs:ti,ab,kw OR donkey:ti,ab,kw OR donkeys:ti,ab,kw OR drosophila:ti,ab,kw OR 'drosophila melanogaster':ti,ab,kw OR duck:ti,ab,kw OR duckling:ti,ab,kw OR ducklings:ti,ab,kw OR ducks:ti,ab,kw OR equid:ti,ab,kw OR equids:ti,ab,kw OR equine:ti,ab,kw OR equines:ti,ab,kw OR feline:ti,ab,kw OR felines:ti,ab,kw OR ferret:ti,ab,kw OR ferrets:ti,ab,kw OR finch:ti,ab,kw OR finches:ti,ab,kw OR fish:ti,ab,kw OR flatworm:ti,ab,kw OR flatworms:ti,ab,kw OR fox:ti,ab,kw OR foxes:ti,ab,kw OR frog:ti,ab,kw OR frogs:ti,ab,kw OR 'fruit flies':ti,ab,kw OR 'fruit fly':ti,ab,kw OR 'g mellonella':ti,ab,kw OR 'galleria mellonella':ti,ab,kw OR geese:ti,ab,kw OR gerbil:ti,ab,kw OR gerbils:ti,ab,kw OR goat:ti,ab,kw OR goats:ti,ab,kw OR goose:ti,ab,kw OR gorilla:ti,ab,kw OR gorillas:ti,ab,kw OR hamster:ti,ab,kw OR hamsters:ti,ab,kw OR hare:ti,ab,kw OR hares:ti,ab,kw OR heifer:ti,ab,kw OR heifers:ti,ab,kw OR horse:ti,ab,kw OR horses:ti,ab,kw OR insect:ti,ab,kw OR insects:ti,ab,kw OR jellyfish:ti,ab,kw OR kangaroo:ti,ab,kw OR kangaroos:ti,ab,kw OR kitten:ti,ab,kw OR kittens:ti,ab,kw OR lagomorph:ti,ab,kw OR lagomorphs:ti,ab,kw OR lamb:ti,ab,kw OR lambs:ti,ab,kw OR llama:ti,ab,kw OR llamas:ti,ab,kw OR macaque:ti,ab,kw OR macaques:ti,ab,kw OR

macaw:ti,ab,kw OR macaws:ti,ab,kw OR marmoset:ti,ab,kw OR marmosets:ti,ab,kw OR mice:ti,ab,kw OR minipig:ti,ab,kw OR minipigs:ti,ab,kw OR mink:ti,ab,kw OR minks:ti,ab,kw OR monkey:ti,ab,kw OR monkeys:ti,ab,kw OR mouse:ti,ab,kw OR mule:ti,ab,kw OR mules:ti,ab,kw OR nematode:ti,ab,kw OR nematodes:ti,ab,kw OR octopus:ti,ab,kw OR octopuses:ti,ab,kw OR orangutan:ti,ab,kw OR 'orang-utan':ti,ab,kw OR orangutans:ti,ab,kw OR 'orang-utans':ti,ab,kw OR oxen:ti,ab,kw OR parrot:ti,ab,kw OR parrots:ti,ab,kw OR pig:ti,ab,kw OR pigeon:ti,ab,kw OR pigeons:ti,ab,kw OR piglet:ti,ab,kw OR piglets:ti,ab,kw OR pigs:ti,ab,kw OR porcine:ti,ab,kw OR primate:ti,ab,kw OR primates:ti,ab,kw OR quail:ti,ab,kw OR rabbit:ti,ab,kw OR rabbits:ti,ab,kw OR rat:ti,ab,kw OR rats:ti,ab,kw OR reptile:ti,ab,kw OR reptiles:ti,ab,kw OR rodent:ti,ab,kw OR rodents:ti,ab,kw OR ruminant:ti,ab,kw OR ruminants:ti,ab,kw OR salmon:ti,ab,kw OR sheep:ti,ab,kw OR shrimp:ti,ab,kw OR slug:ti,ab,kw OR slugs:ti,ab,kw OR swine:ti,ab,kw OR tamarin:ti,ab,kw OR tamarins:ti,ab,kw OR toad:ti,ab,kw OR toads:ti,ab,kw OR trout:ti,ab,kw OR urchin:ti,ab,kw OR urchins:ti,ab,kw OR vole:ti,ab,kw OR voles:ti,ab,kw OR waxworm:ti,ab,kw OR waxworms:ti,ab,kw OR worm:ti,ab,kw OR worms:ti,ab,kw OR xenopus:ti,ab,kw OR 'zebra fish':ti,ab,kw OR zebrafish:ti,ab,kw OR cell:ti,ab,kw) NOT (human:ti,ab,kw OR humans:ti,ab,kw OR patient:ti,ab,kw OR patients:ti,ab,kw)

#14. #12 OR #13

#15. #10 AND #11

#16. #15 NOT #14

#17. congress*:it OR letter:it OR comment:it OR editorial:it OR 'published erratum':it OR review:it OR 'meta analysis':it OR 'systematic review':it

#18. #16 NOT #17

#19. english:la

#20. #18 AND #19

Table S1-C. Final search strategy for Cochrane Central

ID Search Hits

#1 MeSH descriptor: [Fatty Liver] explode all trees 1721

#2 MeSH descriptor: [Non-alcoholic Fatty Liver Disease] explode all trees 1367

#3 (((("non-alcoholic" or nonalcoholic) and ("fatty liver*" or steatohepatit* or "liver steatos*" or "hepatic steatos*" or "visceral steatos*" or "steatohepatitis" or "steatosis of liver")) or ("Nonalcoholic Steatohepatitis" or NAFLD or NASH or non-AFLD or non-ASH))):ti,ab,kw (Word variations have been searched) 4481

#4 #1 or #2 or #3 4688

#5 MeSH descriptor: [Child] explode all trees 62386

#6 MeSH descriptor: [Infant] explode all trees 35368

#7 MeSH descriptor: [Adolescent] explode all trees 110885

#8 (child* or adolescent* or infant* or juvenil* or pediatric* or paediatric* or "young person*" or "young people" or youth* or "young adult*"):ti,ab,kw (Word variations have been searched) 341688

#9 #5 or #6 or #7 or #8 353715

#10 #4 and #9 583

#11 (randomized controlled trial):pt (Word variations have been searched) 4

#12 (controlled clinical trial):pt (Word variations have been searched) 2

#13 (random*):ti,ab,kw (Word variations have been searched) 1197413

#14 (placebo*):ti,ab,kw (Word variations have been searched) 355753

#15 (single blind*):ti,ab,kw (Word variations have been searched) 104385

#16 (double blind*):ti,ab,kw (Word variations have been searched) 327775

#17 (triple blind*):ti,ab,kw (Word variations have been searched) 4690

#18 MeSH descriptor: [Drug Therapy] explode all trees 148743

#19 MeSH descriptor: [Clinical Trials as Topic] explode all trees 48738

#20 (trial):ti,ab,kw (Word variations have been searched) 1028406

#21 (groups):ti,ab,kw (Word variations have been searched) 903735

#22 #11 OR #12 OR #13 OR #14 OR #15 OR #16 OR #17 OR #18 OR #19 OR #20 OR #21 1577555

#23 (((alpaca or alpacas or amphibian or amphibians or animal or animals or antelope or armadillo or armadillos or avian or baboon or baboons or beagle or beagles or bee or bees or bird or birds or bison or bovine or buffalo or buffaloes or buffalos or "c elegans" or "Caenorhabditis elegans" or camel or camels or canine or canines or carp or cats or cattle or chick or chicken or chickens or chicks or chimp or chimpanze or chimpanzees or chimps or cow or cows or "D melanogaster" or "dairy calf" or "dairy calves" or deer or dog or dogs or donkey or donkeys or drosophila or "Drosophila melanogaster" or duck or duckling or ducklings or ducks or equid or equids or equine or equines or feline or felines or ferret or ferrets or finch or finches or fish or flatworm or flatworms or fox or foxes or frog or frogs or "fruit flies" or "fruit fly" or "G mellonella" or "Galleria mellonella" or geese or gerbil or gerbils or goat or goats or goose or gorilla or gorillas or hamster or hamsters or hare or hares or heifer or heifers or horse or horses or insect or insects or jellyfish or kangaroo or kangaroos or kitten or kittens or lagomorph or lagomorphs or lamb or lambs or llama or llamas or macaque or macaques or macaw or macaws or marmoset or marmosets or mice or minipig or minipigs or mink or minks or monkey or monkeys or mouse or mule or mules or nematode or nematodes or octopus or octopuses or orangutan or "orang-utan" or orangutans or "orang-utans" or oxen or parrot or parrots or pig or pigeon or

pigeons or piglet or piglets or pigs or porcine or primate or primates or quail or rabbit or rabbits or rat or rats or reptile or reptiles or rodent or rodents or ruminant or ruminants or salmon or sheep or shrimp or slug or slugs or swine or tamarin or tamarins or toad or toads or trout or urchin or urchins or vole or voles or waxworm or waxworms or worm or worms or xenopus or "zebra fish" or zebrafish or cell) not (human or humans or patient or patients))):ti,ab,kw (Word variations have been searched) 21773

#24 #22 not #23 1562622

#25 #10 and #24 534

#26 ((congress* or letter or comment or editorial or published erratum or review or Meta-Analysis or Systematic Review)):pt 16664

#27 #25 not #26 in Trials 532

Table S1-D. Final search strategy for PUBMED

#	Query	Results
1	fatty liver[MeSH Terms]	43,827
2	Non-alcoholic Fatty Liver Disease[MeSH Terms]	21,322
3	((("non-alcoholic" or nonalcoholic) and ("fatty liver*" or steatohepatit* or "liver steatos*" or "hepatic steatos*" or "visceral steatos*" or "steatohepatitis" or "steatosis of liver")) or ("Nonalcoholic Steatohepatitis" or NAFLD or NASH or non-AFLD or non-ASH))	52,245
4	#1 OR #2 OR #3	69,575
5	Child[MeSH Terms]	2,117,005
6	Infant[MeSH Terms]	1,236,506
7	Adolescent[MeSH Terms]	2,197,412
8	child* or adolescent* or infant* or juvenil* or pediatric* or paediatric* or "young person*" or "young people" or youth* or "young adult*"	5,568,295
9	#5 OR #6 OR #7 OR #8	5,568,295
10	#4 AND #9	11,686
11	randomized controlled trial[Publication Type]	584,958
12	controlled clinical trial[Publication Type]	675,204
13	((((((((random*[Title/Abstract]) OR (placebo*[Title/Abstract])) OR (single blind*[Title/Abstract])) OR (double blind*[Title/Abstract])) OR (triple blind*[Title/Abstract])) OR (drug therapy[MeSH Subheading])) OR (clinical trials as topic[MeSH Major Topic]))) OR (groups[Title/Abstract])) OR (trial[Title/Abstract])	5,833,110
14	#11 OR #12 OR #13	5,917,052
15	("animals"[MeSH Terms]) NOT "humans"[MeSH Terms]	5,077,058
16	((alpaca[Title/Abstract] OR alpacas[Title/Abstract] OR amphibian[Title/Abstract] OR amphibians[Title/Abstract] OR animal[Title/Abstract] OR animals[Title/Abstract] OR antelope[Title/Abstract] OR armadillo[Title/Abstract] OR armadillos[Title/Abstract] OR avian[Title/Abstract] OR baboon[Title/Abstract] OR baboons[Title/Abstract] OR beagle[Title/Abstract] OR beagles[Title/Abstract] OR bee[Title/Abstract] OR bees[Title/Abstract] OR bird[Title/Abstract] OR birds[Title/Abstract] OR bison[Title/Abstract] OR bovine[Title/Abstract] OR buffalo[Title/Abstract] OR buffaloes[Title/Abstract] OR buffalos[Title/Abstract] OR "c elegans"[Title/Abstract] OR "Caenorhabditis elegans"[Title/Abstract] OR camel[Title/Abstract] OR camels[Title/Abstract] OR canine[Title/Abstract] OR canines[Title/Abstract] OR carp[Title/Abstract] OR cats[Title/Abstract] OR cattle[Title/Abstract] OR chick[Title/Abstract] OR chicken[Title/Abstract] OR chickens[Title/Abstract] OR chicks[Title/Abstract] OR chimp[Title/Abstract] OR chimpanze[Title/Abstract] OR chimpanzees[Title/Abstract] OR chimps[Title/Abstract] OR cow[Title/Abstract] OR cows[Title/Abstract] OR "D melanogaster"[Title/Abstract] OR "dairy calf"[Title/Abstract] OR "dairy calves"[Title/Abstract] OR deer[Title/Abstract] OR dog[Title/Abstract] OR dogs[Title/Abstract] OR donkey[Title/Abstract] OR donkeys[Title/Abstract] OR drosophila[Title/Abstract] OR "Drosophila	5,628,527

	melanogaster"[Title/Abstract] OR duck[Title/Abstract] OR duckling[Title/Abstract] OR ducklings[Title/Abstract] OR ducks[Title/Abstract] OR equid[Title/Abstract] OR equids[Title/Abstract] OR equine[Title/Abstract] OR equines[Title/Abstract] OR feline[Title/Abstract] OR felines[Title/Abstract] OR ferret[Title/Abstract] OR ferrets[Title/Abstract] OR finch[Title/Abstract] OR finches[Title/Abstract] OR fish[Title/Abstract] OR flatworm[Title/Abstract] OR flatworms[Title/Abstract] OR fox[Title/Abstract] OR foxes[Title/Abstract] OR frog[Title/Abstract] OR frogs[Title/Abstract] OR "fruit flies"[Title/Abstract] OR "fruit fly"[Title/Abstract] OR "G mellonella"[Title/Abstract] OR "Galleria mellonella"[Title/Abstract] OR geese[Title/Abstract] OR gerbil[Title/Abstract] OR gerbils[Title/Abstract] OR goat[Title/Abstract] OR goats[Title/Abstract] OR goose[Title/Abstract] OR gorilla[Title/Abstract] OR gorillas[Title/Abstract] OR hamster[Title/Abstract] OR hamsters[Title/Abstract] OR hare[Title/Abstract] OR hares[Title/Abstract] OR heifer[Title/Abstract] OR heifers[Title/Abstract] OR horse[Title/Abstract] OR horses[Title/Abstract] OR insect[Title/Abstract] OR insects[Title/Abstract] OR jellyfish[Title/Abstract] OR kangaroo[Title/Abstract] OR kangaroos[Title/Abstract] OR kitten[Title/Abstract] OR kittens[Title/Abstract] OR lagomorph[Title/Abstract] OR lagomorphs[Title/Abstract] OR lamb[Title/Abstract] OR lambs[Title/Abstract] OR llama[Title/Abstract] OR llamas[Title/Abstract] OR macaque[Title/Abstract] OR macaques[Title/Abstract] OR macaw[Title/Abstract] OR macaws[Title/Abstract] OR marmoset[Title/Abstract] OR marmosets[Title/Abstract] OR mice[Title/Abstract] OR minipig[Title/Abstract] OR minipigs[Title/Abstract] OR mink[Title/Abstract] OR minks[Title/Abstract] OR monkey[Title/Abstract] OR monkeys[Title/Abstract] OR mouse[Title/Abstract] OR mule[Title/Abstract] OR mules[Title/Abstract] OR nematode[Title/Abstract] OR nematodes[Title/Abstract] OR octopus[Title/Abstract] OR octopuses[Title/Abstract] OR orangutan[Title/Abstract] OR "orang-utan"[Title/Abstract] OR orangutans[Title/Abstract] OR "orang-utans"[Title/Abstract] OR oxen[Title/Abstract] OR parrot[Title/Abstract] OR parrots[Title/Abstract] OR pig[Title/Abstract] OR pigeon[Title/Abstract] OR pigeons[Title/Abstract] OR piglet[Title/Abstract] OR piglets[Title/Abstract] OR pigs[Title/Abstract] OR porcine[Title/Abstract] OR primate[Title/Abstract] OR primates[Title/Abstract] OR quail[Title/Abstract] OR rabbit[Title/Abstract] OR rabbits[Title/Abstract] OR rat[Title/Abstract] OR rats[Title/Abstract] OR reptile[Title/Abstract] OR reptiles[Title/Abstract] OR rodent[Title/Abstract] OR rodents[Title/Abstract] OR ruminant[Title/Abstract] OR ruminants[Title/Abstract] OR salmon[Title/Abstract] OR sheep[Title/Abstract] OR shrimp[Title/Abstract] OR slug[Title/Abstract] OR slugs[Title/Abstract] OR swine[Title/Abstract] OR tamarin[Title/Abstract] OR tamarins[Title/Abstract] OR toad[Title/Abstract] OR toads[Title/Abstract] OR trout[Title/Abstract] OR urchin[Title/Abstract] OR urchins[Title/Abstract] OR vole[Title/Abstract] OR voles[Title/Abstract] OR waxworm[Title/Abstract] OR waxworms[Title/Abstract] OR worm[Title/Abstract] OR worms[Title/Abstract] OR xenopus[Title/Abstract] OR "zebra fish"[Title/Abstract] OR zebrafish[Title/Abstract] OR cell[Title/Abstract]) NOT (human[Title/Abstract] OR humans[Title/Abstract] OR patient[Title/Abstract] OR patients[Title/Abstract]))	

17	#15 OR #16	7,090,506
18	#10 AND #14	3,028
19	#18 NOT #17	2,667
20	((((((((("congress*"[Publication Type]) OR "letter"[Publication Type]) OR "comment"[Publication Type]) OR "editorial"[Publication Type]) OR "published erratum"[Publication Type]) OR "review"[Publication Type]) OR "Meta-Analysis"[Publication Type]) OR "Systematic Review"[Publication Type]) OR "clinical protocols"[MeSH Terms]) OR protocol[Text Word]	6,017,654
21	#19 NOT #20	2,167
22	english[Language]	30,293,823
23	#21 AND #22	2,009

Table S1-E. Final search strategy for Web of Science

#	Search Query	Results
1	((("non-alcoholic" or nonalcoholic) and ("fatty liver*" or steatohepatit* or "liver steatos*" or "hepatic steatos*" or "visceral steatos*" or "steatohepatitis" or "steatosis of liver")) or ("Nonalcoholic Steatohepatitis" or NAFLD or NASH or non-AFLD or non-ASH)) (Topic)	111,898
2	TI=((("non-alcoholic" or nonalcoholic) and ("fatty liver*" or steatohepatit* or "liver steatos*" or "hepatic steatos*" or "visceral steatos*" or "steatohepatitis" or "steatosis of liver")) or ("Nonalcoholic Steatohepatitis" or NAFLD or NASH or non-AFLD or non-ASH))	46,812
3	AB=((("non-alcoholic" or nonalcoholic) and ("fatty liver*" or steatohepatit* or "liver steatos*" or "hepatic steatos*" or "visceral steatos*" or "steatohepatitis" or "steatosis of liver")) or ("Nonalcoholic Steatohepatitis" or NAFLD or NASH or non-AFLD or non-ASH))	79,298
4	#1 OR #2 OR #3	111,898
5	TS=(Child)	4,090,585
6	TS=(Infant)	1,691,070
7	TS=(Adolescent)	2,789,376
8	(TI=(child* or adolescent* or infant* or juvenil* or pediatric* or paediatric* or "young person*" or "young people" or youth* or "young adult*")) OR AB=(child* or adolescent* or infant* or juvenil* or pediatric* or paediatric* or "young person*" or "young people" or youth* or "young adult*")	4,167,836
9	#5 OR #6 OR #7 OR #8	6,821,119
10	(TI=((((control* NEAR/3 study) OR (control* NEAR/3 trial) OR (randomized NEAR/3 study) OR (randomized NEAR/3 trial) OR (randomised NEAR/3 study) OR (randomised NEAR/3 trial) OR "pragmatic clinical trial" OR (random* NEAR/1 allocat*) OR (doubl* NEAR/1 blind*) OR (doubl* NEAR/1 mask*) OR (singl* NEAR/1 blind*) OR (singl* NEAR/1 mask*) OR (tripl* NEAR/1 blind*) OR (tripl* NEAR/1 mask*) OR (trebl* NEAR/1 blind*) OR (trebl* NEAR/1 mask*) OR "latin square" OR placebo* OR nocebo* OR random*)))) OR AB=((((control* NEAR/3 study) OR (control* NEAR/3 trial) OR (randomized NEAR/3 study) OR (randomized NEAR/3 trial) OR (randomised NEAR/3 study) OR (randomised NEAR/3 trial) OR "pragmatic clinical trial" OR (random* NEAR/1 allocat*) OR (doubl* NEAR/1 blind*) OR (doubl* NEAR/1 mask*) OR (singl* NEAR/1 blind*) OR (singl* NEAR/1 mask*) OR (tripl* NEAR/1 blind*) OR (tripl* NEAR/1 mask*) OR (trebl* NEAR/1 blind*) OR (trebl* NEAR/1 mask*) OR "latin square" OR placebo* OR nocebo* OR random*)))	3,578,451

11	(TI=(((alpaca OR alpacas OR amphibian OR amphibians OR animal OR animals OR antelope OR armadillo OR armadillos OR avian OR baboon OR baboons OR beagle OR beagles OR bee OR bees OR bird OR birds OR bison OR bovine OR buffalo OR buffaloes OR buffalos OR "c elegans" OR "Caenorhabditis elegans" OR camel OR camels OR canine OR canines OR carp OR cats OR cattle OR chick OR chicken OR chickens OR chicks OR chimp OR chimpanze OR chimpanzees OR chimps OR cow OR cows OR "D melanogaster" OR "dairy calf" OR "dairy calves" OR deer OR dog OR dogs OR donkey OR donkeys OR drosophila OR "Drosophila melanogaster" OR duck OR duckling OR ducklings OR ducks OR equid OR equids OR equine OR equines OR feline OR felines OR ferret OR ferrets OR finch OR finches OR fish OR flatworm OR flatworms OR fox OR foxes OR frog OR frogs OR "fruit flies" OR "fruit fly" OR "G mellonella" OR "Galleria mellonella" OR geese OR gerbil OR gerbils OR goat OR goats OR goose OR gorilla OR gorillas OR hamster OR hamsters OR hare OR hares OR heifer OR heifers OR horse OR horses OR insect OR insects OR jellyfish OR kangaroo OR kangaroos OR kitten OR kittens OR lagomorph OR lagomorphs OR lamb OR lambs OR llama OR llamas OR macaque OR macaques OR macaw OR macaws OR marmoset OR marmosets OR mice OR minipig OR minipigs OR mink OR minks OR monkey OR monkeys OR mouse OR mule OR mules OR nematode OR nematodes OR octopus OR octopuses OR orangutan OR "orang-utan" OR orangutans OR "orang-utans" OR oxen OR parrot OR parrots OR pig OR pigeon OR pigeons OR piglet OR piglets OR pigs OR porcine OR primate OR primates OR quail OR rabbit OR rabbits OR rat OR rats OR reptile OR reptiles OR rodent OR rodents OR ruminant OR ruminants OR salmon OR sheep OR shrimp OR slug OR slugs OR swine OR tamarin OR tamarins OR toad OR toads OR trout OR urchin OR urchins OR vole OR voles OR waxworm OR waxworms OR worm OR worms OR xenopus OR "zebra fish" OR zebrafish OR cell*) NOT (human OR humans or patient or patients)))) OR AB=(((alpaca OR alpacas OR amphibian OR amphibians OR animal OR animals OR antelope OR armadillo OR armadillos OR avian OR baboon OR baboons OR beagle OR beagles OR bee OR bees OR bird OR birds OR bison OR bovine OR buffalo OR buffaloes OR buffalos OR "c elegans" OR "Caenorhabditis elegans" OR camel OR camels OR canine OR canines OR carp OR cats OR cattle OR chick OR chicken OR chickens OR chicks OR chimp OR chimpanze OR chimpanzees OR chimps OR cow OR cows OR "D melanogaster" OR "dairy calf" OR "dairy calves" OR deer OR dog OR dogs OR donkey OR donkeys OR drosophila OR "Drosophila melanogaster" OR duck OR duckling OR ducklings OR ducks OR equid OR equids OR equine OR equines OR feline OR felines OR ferret OR ferrets OR finch OR finches OR fish OR flatworm OR flatworms OR fox OR foxes OR frog OR frogs OR "fruit flies" OR "fruit fly" OR "G mellonella" OR "Galleria mellonella" OR geese OR gerbil OR gerbils OR goat OR goats OR goose OR gorilla OR gorillas OR hamster OR hamsters OR hare OR hares OR heifer OR heifers OR horse OR horses OR insect OR insects OR jellyfish OR kangaroo OR kangaroos OR kitten OR kittens OR lagomorph OR lagomorphs OR lamb OR lambs OR llama OR llamas OR macaque OR macaques OR macaw OR macaws OR marmoset OR marmosets OR mice OR minipig OR minipigs OR mink OR minks OR monkey OR monkeys OR mouse OR mule OR mules OR nematode OR nematodes OR octopus OR octopuses OR orangutan OR "orang-utan" OR	15,967,555

	orangutans OR "orang-utans" OR oxen OR parrot OR parrots OR pig OR pigeon OR pigeons OR piglet OR piglets OR pigs OR porcine OR primate OR primates OR quail OR rabbit OR rabbits OR rat OR rats OR reptile OR reptiles OR rodent OR rodents OR ruminant OR ruminants OR salmon OR sheep OR shrimp OR slug OR slugs OR swine OR tamarin OR tamarins OR toad OR toads OR trout OR urchin OR urchins OR vole OR voles OR waxworm OR waxworms OR worm OR worms OR xenopus OR "zebra fish" OR zebrafish OR cell*) NOT (human OR humans or patient or patients)))	
12	#10 NOT #11	3,054,238
13	#9 AND #4	7,151
14	#13 AND #12	633
15	TS=(((case* NEAR/3 report*) or (case* NEAR/3 series) or "in sillico" or "in vitro" or "observational study"))	4,364,695
16	#14 NOT #15	612
17	(((TI=(review)) OR TI=("meta analysis")) OR TI=("meta-analysis")) OR TI=("systematic review*")	1,247,559
18	#16 NOT #17	506
19	#16 NOT #17 and English (Languages)	489

Table S1-F. Final search strategy for Scopus

#	Query	Results
1	TITLE-ABS-KEY((("non-alcoholic" or nonalcoholic) and ("fatty liver*" or steatohepatit* or "liver steatos*" or "hepatic steatos*" or "visceral steatos*" or "steatohepatitis" or "steatosis of liver")) or ("Nonalcoholic Steatohepatitis" or NAFLD or NASH or non-AFLD or non-ASH))	92,206
2	TITLE-ABS-KEY (child* OR adolescent* OR infant* OR juvenil* OR pediatric* OR paediatric* OR "young person*" OR "young people" OR youth* OR "young adult*")	6,391,411
3	#1 AND #2	6,812
4	TITLE-ABS-KEY("clinical trials" OR "clinical trials as a topic" OR "randomized controlled trial" OR "Randomized Controlled Trials as Topic" OR "controlled clinical trial" OR "Controlled Clinical Trials as Topic" OR "random allocation" OR "randomly allocated" OR "allocated randomly" OR "Double-Blind Method" OR "Single-Blind Method" OR "Cross-Over Studies" OR "Placebos" OR"cross-over trial" OR "single-blind" OR "double-blind" OR "factorial design" OR "factorial trial") OR (TITLE-ABS-KEY(clinical AND trial* OR trial* OR RCT* OR random* OR blind*))	2,824,681
5	TITLE-ABS-KEY ((alpaca OR alpacas OR amphibian OR amphibians OR animal OR animals OR antelope OR armadillo OR armadillos OR avian OR baboon OR baboons OR beagle OR beagles OR bee OR bees OR bird OR birds OR bison OR bovine OR buffalo OR buffaloes OR buffalos OR "c elegans" OR "Caenorhabditis elegans" OR camel OR camels OR canine OR canines OR carp OR cats OR cattle OR chick OR chicken OR chickens OR chicks OR chimp OR chimpanze OR chimpanzees OR chimps OR cow OR cows OR "D melanogaster" OR "dairy calf" OR "dairy calves" OR deer OR dog OR dogs OR donkey OR donkeys OR drosophila OR "Drosophila melanogaster" OR duck OR duckling OR ducklings OR ducks OR equid OR equids OR equine OR equines OR feline OR felines OR ferret OR ferrets OR finch OR finches OR fish OR flatworm OR flatworms OR fox OR foxes OR frog OR frogs OR "fruit flies" OR "fruit fly" OR "G mellonella" OR "Galleria mellonella" OR geese OR gerbil OR gerbils OR goat OR goats OR goose OR gorilla OR gorillas OR hamster OR hamsters OR hare OR hares OR heifer OR heifers OR horse OR horses OR insect OR insects OR jellyfish OR kangaroo OR kangaroos OR kitten OR kittens OR lagomorph OR lagomorphs OR lamb OR lambs OR llama OR llamas OR macaque OR macaques OR macaw OR macaws OR marmoset OR marmosets OR mice OR minipig OR minipigs OR mink OR minks OR monkey OR monkeys OR mouse OR mule OR mules OR nematode OR nematodes OR octopus OR octopuses OR orangutan OR "orang-utan" OR orangutans OR "orang-utans" OR oxen OR parrot OR parrots OR pig OR pigeon OR pigeons OR piglet OR piglets OR pigs OR porcine OR primate OR primates OR quail OR rabbit OR rabbits OR rat OR rats OR reptile OR reptiles OR rodent OR rodents OR ruminant OR ruminants OR salmon OR sheep OR shrimp OR slug OR slugs OR swine OR tamarin OR tamarins OR toad OR toads OR trout OR urchin OR urchins OR vole OR voles OR waxworm OR waxworms OR worm OR worms OR xenopus OR "zebra fish" OR zebrafish OR cell*) AND NOT (human OR humans or patient or patients))	9,692,989
6	#4 AND NOT #5	2,700,170
7	#3 AND #6	1,141

8	TITLE-ABS-KEY((case* W/3 report*) or (case* W/3 series) or "in sillico" or "in vitro" or "observational study")	5,816,385
9	#7 AND NOT #8	1,063
10	DOCTYPE(bk) OR DOCTYPE(ch) OR DOCTYPE(cp) OR DOCTYPE(cr) OR DOCTYPE(dp) OR DOCTYPE(ed) OR DOCTYPE(le) OR DOCTYPE(le) OR DOCTYPE(mm) OR DOCTYPE(no) OR DOCTYPE(pr) OR DOCTYPE(rp) OR DOCTYPE(tb) OR DOCTYPE(re) OR DOCTYPE(sh) OR DOCTYPE(re)	25,290,466
11	#9 AND NOT #10	748
12	#9 AND NOT #10 AND (LIMIT-TO (DOCTYPE,"ar")) AND (LIMIT-TO (LANGUAGE , "English"))	726

Supplementary File 2. Summary of therapeutic categories

Table S1. Summary of therapeutic categories and mechanisms/pathways

Treatment	Subset 1	Classification 1	Subset 2	Classification 2	Mechanism
rhGH	Lipid lowering	Energy	/	/	① IGF-1 has been proven to be a protective factor against NAFLD, and rhGH improves NAFLD by increasing IGF-1 levels [1].<br>② Improve body mass index and lipid metabolism [2, 3].<br>③ Ameliorat hepatocyte steatosis by suppressing denovo lipogenesis via carbohydrate responsive element-binding protein and fatty acid synthase downregulation [4].<br>④ As a key anabolic hormone, rhGH possesses well-characterized lipolytic effects, especially in visceral adipose tissue [5].
Losartan	ARB	Fibrosis	/	/	Losartan, an angiotensin II receptor blocker, reduces the degree of liver fibrosis, a mechanism that may be associated with the decreased expression of TGF-β1 and TIMP-1 mRNA [6, 7].
Probiotics	Gut-liver axis	Modify gut microbiota	/	/	Enhance the barrier function of epithelial cells and decrease intestinal permeability and endotoxemia in patients with liver disease [8, 9].
Metformin	Insulin sensitizers	Energy	/	/	Metformin: insulin sensitizers, increase liver sensitivity to insulin and reduce ALT levels [10, 11].
Vitamin D	Anti-oxidant	Inflammation	/	/	① Inhibition of inflammation and oxidative stress [12].<br>② Vitamin D exerts its anti-inflammatory effect through inhibition of the activity of nuclear factor kappa-β (NF-κβ), which has a key role for the release of inflammatory mediators and cytokines [13].
L-carnitine	Anti-inflammatory	Inflammation	Lipid metabolism	Energy	① Increase hepatic mitochondrial beta oxidation of long chain fatty acids [14].<br>② L-carnitine has essential intracellular and metabolic functions and can stimulate mitochondrial functions. It is essential for long chain fatty acid beta oxidation and regulation of the mitochondrial acyl-CoA/CoA ratio and stabilization of cell membranes. It transports long-chain acyl groups into the mitochondrial matrix. The inhibition of beta oxidation leads to the accumulation of lipids within hepatocytes [15].<br>③ Regulates peroxisome proliferator activator receptor gamma in the liver [16].
Silymarin	Reducing fibrosis	Fibrosis	Anti-inflammatory	Inflammation	① Increase hepatocyte protein synthesis, which needs for repairing liver tissue, slowing fibrosis process and even improvement of liver fibrosis [17].<br>② The anti-inflammatory effect of silymarin is due to inhibiting leukotriene and prostaglandin synthesis, inhibit Kupffer cells, stabilizing mast cells and avoiding neutrophil migration in human body [18, 19].
CBDR	Anti-oxidant	Inflammation	/	/	Exogenous supplementation of precursor amino acids (especially cysteine) to support intracellular glutathione synthesis [20, 21].

Table S1. Summary of therapeutic categories and mechanisms/pathways (***Continued***).

Treatment	Subset 1	Classification 1	Subset 2	Classification 2	Mechanism
DHA	Fatty Acids	Energy	/	/	① In children, DHA supplementation improves liver steatosis and is able to reduce the levels of serum ALT and triglycerides, and to improve insulin sensitivity [22].<br>② DHA downregulates hepatic triglyceride accumulation by decreasing the transcriptional activity of the sterol regulatory element binding protein-1 and by activating the peroxisome proliferator-activated receptor-mediated pathway of lipid catabolism [23].
Omega-3	Fatty Acids	Energy	/	/	Omega-3 fatty acids improve lipid metabolism by regulating miR-34a [24, 25].
PUFA	Fatty Acids	Energy	/	/	Omega-3 PUFA, especially eicosapentaenoic acid (C20: 5n3, EPA) and docosahexaenoic acid (C22: 6n3, DHA), by regulating gene transcription factors (i.e., PPARα, PPARγ, SREBP-1, ChREBP), can control key pathways involved in hepatic lipid metabolism [26, 27].
Vitamin E	Anti-oxidant	Inflammation	/	/	The useful effect of vitamin E in patients with steatohepatitis has been attributed to its antioxidant effects. Oxidative stress plays an essential role in NAFLD pathogenesis [28].
VSL#3	Gut-liver axis	Modify gut microbiota	/	/	Reset the 'leaky gut', and offered protection against NAFLD development and its progression to NASH by modulating the expression of nuclear receptors and correcting insulin resistance in the liver and the adipose tissues [29].

Abbreviations: CBDR – Cysteamine bitartrate delayed release; DHA – Docosahexaenoic acid; ARB – Angiotensin receptor blocker; TGF-β – 1transforming growth factor-β1; TIMP1 – Tissue inhibitors of metalloproteinase 1; PUFA – Polyunsaturated fatty acid; rhGH – Recombinant human growth hormone; IGF-1 – Insulin-like growth factors; VSL#3 – a mixture of eight probiotic strains (Streptococcus thermophilus, bifidobacteria [B. breve, B. infantis, B. longum], Lactobacillus acidophilus, L. plantarum, L. paracasei, and L. delbrueckii subsp. bulgaricus); NAFLD – Nonalcoholic fatty liver disease; NASH – Nonalcoholic steatohepatitis;

References:

[1] Akcam M, Boyaci A, Pirgon O, *et al.* Therapeutic effect of metformin and vitamin E versus prescriptive diet in obese adolescents with fatty liver. International Journal for Vitamin & Nutrition Research, 2011, 81(6): 398-406. https://doi.org/https://dx.doi.org/10.1024/0300-9831/a000086.

[2] Liang S, Xue J, Li G. Effects of recombinant human growth hormone administration on cardiovascular risk factors in obese children with relative growth hormone deficiency. Lipids Health Dis, 2018, 17(1): 66. https://doi.org/10.1186/s12944-018-0721-9.

[3] Wu J, Zhao F, Zhang Y, *et al.* Effect of One-Year Growth Hormone Therapy on Cardiometabolic Risk Factors in Boys with Obesity. Biomed Res Int, 2020, 2020: 2308124. https://doi.org/10.1155/2020/2308124.

[4] Baumgartner C, Metz M, Beghini M, *et al.* Growth Hormone Promotes Hepatic Triglyceride Export in Humans. J Clin Endocrinol Metab, 2025, 110(12): 3420-3429. https://doi.org/10.1210/clinem/dgaf155.

[5] Gravhølt CH, Schmitz O, Simonsen L, *et al*. Effects of a physiological GH pulse on interstitial glycerol in abdominal and femoral adipose tissue. Am J Physiol, 1999, 277(5): E848-854. https://doi.org/10.1152/ajpendo.1999.277.5.E848.
[6] Avasthi D, Zerilli N, Shaikh F, *et al.* Impact of Losartan on Portal Hypertension and Liver Cirrhosis: A Systematic Review. Cureus, 2025, 17(5): e83309. https://doi.org/10.7759/cureus.83309.
[7] Vos MB, Van Natta ML, Blondet NM, *et al.* Randomized placebo-controlled trial of losartan for pediatric NAFLD. Hepatology, 2022, 76(2): 429-444. https://doi.org/10.1002/hep.32403.
[8] Rodrigo T, Dulani S, Nimali Seneviratne S, *et al.* Effects of probiotics combined with dietary and lifestyle modification on clinical, biochemical, and radiological parameters in obese children with nonalcoholic fatty liver disease/nonalcoholic steatohepatitis: a randomized clinical trial. Clinical And Experimental Pediatrics, 2022, 65(6): 304-311. https://doi.org/https://dx.doi.org/10.3345/cep.2021.00787.
[9] Ghosh S, Ghosh AJ, Adhikari MD, *et al.* Probiotics for managing non-alcoholic fatty liver disease: efficacy and mechanistic insights. J Diabetes Metab Disord, 2026, 25(1): 42. https://doi.org/10.1007/s40200-026-01861-3.
[10] Homaei A, Alhadad M, Arad B, *et al.* Effect of Metformin or Vitamin E on Ultrasonographic Grade and Biochemical Findings of Children and Adolescents with Nonalcoholic Fatty Liver Disease: A Randomized Clinical Trial.Compr. Pediatr, 2022, 13(2). https://doi.org/10.5812/compreped-123944.
[11] Malin SK, Heiston EM, Battillo DJ, *et al.* Metformin attenuates metabolic insulin sensitivity and insulin-stimulated carbohydrate oxidation after high-intensity exercise training in adults at risk for metabolic syndrome. Diabetes Obes Metab, 2026, 28(4): 2941-2952. https://doi.org/10.1111/dom.70478.
[12] Fuentes-Barría H, Aguilera-Eguía R, Alarcón-Rivera M, *et al.* Vitamin D and Metabolic Dysfunction-Associated Steatotic Liver Disease: Molecular Mechanisms and Clinical Implications-A Narrative Review. Int J Mol Sci, 2026, 27(6). https://doi.org/10.3390/ijms27062532.
[13] Ding C, Wilding JP, Bing C. 1,25-dihydroxyvitamin D3 protects against macrophage-induced activation of NFκB and MAPK signalling and chemokine release in human adipocytes. PLoS One, 2013, 8(4): e61707. https://doi.org/10.1371/journal.pone.0061707.
[14] Saneian H, Khalilian L, Heidari-Beni M, *et al.* Effect of l-carnitine supplementation on children and adolescents with nonalcoholic fatty liver disease (NAFLD): a randomized, triple-blind, placebo-controlled clinical trial. J Pediatr Endocrinol Metab, 2021, 34(7): 897-904. https://doi.org/https://dx.doi.org/10.1515/jpem-2020-0642.
[15] Jin Y, Zhao J, Wang X, *et al.* Effects of prenatal DINP exposure induced hepatic steatosis and underlying mechanism. Toxicol Appl Pharmacol, 2026, 507: 117707. https://doi.org/10.1016/j.taap.2026.117707.
[16] Li JL, Wang QY, Luan HY, *et al.* Effects of L-carnitine against oxidative stress in human hepatocytes: involvement of peroxisome proliferator-activated receptor alpha. J Biomed Sci, 2012, 19(1): 32. https://doi.org/10.1186/1423-0127-19-32.
[17] Amjad MZ, Hassan MU, Rehman M, *et al.* Impact of Silymarin Supplementation on Liver Function and Enzyme Profiles in Diverse Chronic Liver Disease Etiologies. Cureus, 2024, 16(12): e76313. https://doi.org/10.7759/cureus.76313.
[18] Shaker E, Mahmoud H, Mnaa S. Silymarin, the antioxidant component and Silybum marianum extracts prevent liver damage. Food Chem Toxicol, 2010, 48(3): 803-806. https://doi.org/10.1016/j.fct.2009.12.011.
[19] Schrieber SJ, Wen Z, Vourvahis M, *et al.* The pharmacokinetics of silymarin is altered in patients with hepatitis C virus and nonalcoholic Fatty liver disease and correlates with plasma caspase-3/7 activity. Drug Metab Dispos, 2008, 36(9): 1909-1916. https://doi.org/10.1124/dmd.107.019604.
[20] Langman CB, Greenbaum LA, Sarwal M, *et al.* A randomized controlled crossover trial with delayed-release cysteamine bitartrate in nephropathic cystinosis:

effectiveness on white blood cell cystine levels and comparison of safety. Clin J Am Soc Nephrol, 2012, 7(7): 1112-1120. https://doi.org/10.2215/cjn.12321211.
[21] Schwimmer JB, Lavine JE, Wilson LA, *et al.* In Children With Nonalcoholic Fatty Liver Disease, Cysteamine Bitartrate Delayed Release Improves Liver Enzymes but Does Not Reduce Disease Activity Scores. Gastroenterology, 2016, 151(6): 1141-1154.e1149. https://doi.org/10.1053/j.gastro.2016.08.027.
[22] Nobili V, Bedogni G, Alisi A, *et al.* Docosahexaenoic acid supplementation decreases liver fat content in children with non-alcoholic fatty liver disease: double-blind randomised controlled clinical trial. Arch Dis Child, 2011, 96(4): 350-353. https://doi.org/10.1136/adc.2010.192401.
[23] Lorente-Cebrián S, Costa AG, Navas-Carretero S, *et al.* Role of omega-3 fatty acids in obesity, metabolic syndrome, and cardiovascular diseases: a review of the evidence. J Physiol Biochem, 2013, 69(3): 633-651. https://doi.org/10.1007/s13105-013-0265-4.
[24] Li L, Tang Y, Wang X, *et al.* Omega-3 fatty acids improve lipid metabolism by regulating miR-34a. Sci Rep, 2026. https://doi.org/10.1038/s41598-026-43353-7.
[25] Kim SJ, Cho SH, Yun JM. Omega-3 polyunsaturated fatty acids and nonalcoholic fatty liver disease in adults: A meta-analysis of randomized controlled trials. Clin Nutr, 2025, 50: 164-174. https://doi.org/10.1016/j.clnu.2025.05.013.
[26] Jump DB. N-3 polyunsaturated fatty acid regulation of hepatic gene transcription. Curr Opin Lipidol, 2008, 19(3): 242-247. https://doi.org/10.1097/MOL.0b013e3282ffaf6a.
[27] Boyraz M, Pirgon Ö, Dündar B, *et al.* Long-term treatment with n-3 polyunsaturated fatty acids as a monotherapy in children with nonalcoholic fatty liver disease. JCRPE Journal of Clinical Research in Pediatric Endocrinology, 2015, 7(2): 121-127. https://doi.org/10.4274/jcrpe.1749.
[28] Shan M, Apolinario MEC, Tokumaru T, *et al.* Vitamin E Modulates Hepatic Extracellular Adenosine Signaling to Attenuate Metabolic Dysfunction-Associated Steatotic Liver Disease (MASLD). Int J Mol Sci, 2026, 27(2). https://doi.org/10.3390/ijms27020614.
[29] Alisi A, Bedogni G, Baviera G, *et al.* Randomised clinical trial: The beneficial effects of VSL#3 in obese children with non-alcoholic steatohepatitis. Alimentary Pharmacology & Therapeutics, 2014, 39(11): 1276-1285. https://doi.org/https://dx.doi.org/10.1111/apt.12758.

Supplementary File 3. Risk of bias assessment

Table S1. Risk of bias assessment

No.	Trial name	Random sequence generation	Allocation concealment	Blinding of participants and personnel	Blinding of outcome assessment	Incomplete outcome data	Selecting reporting	Other source of bias	Overall
1	Xue *et al* (2022)	Unclear	Unclear	Low	Low	High	Low	Low	High
2	Vos *et al* (2022)	Low	Low	Low	Low	Low	Low	Low	Low
3	Rodrigo *et al* (2022)	Low	Low	Low	Low	Low	Low	Low	Low
4	Homaei *et al* (2022)	Low	Low	Low	Low	Low	Unclear	Low	Unclear
5	El Amrousy *et al* (2022)	Low	Low	Low	Low	Low	Low	Low	Low
6	Saneian *et al* (2021)	Low	Unclear	Low	Low	Low	Low	Low	Unclear
7	Namakin *et al* (2021)	Unclear	Unclear	Low	Low	Low	High	Low	High
8	Lavine *et al* (2011)	Low	Low	Low	Low	Low	Low	Low	Low
9	Famouri *et al* (2017)	Low	Low	Low	Low	Low	Low	Low	Low
10	Famouri *et al* (2017)	High	High	Low	Low	Low	Low	Low	High
11	Schwimmer *et al* (2016)	Low	Low	Low	Low	Low	Low	Low	Low
12	Pacifico *et al* (2015)	Low	Low	Low	Low	Low	Low	Low	Low
13	Janczyk *et al* (2015)	Low	Low	Low	Low	Low	Low	Low	Low
14	Boyraz *et al* (2015)	Unclear	Unclear	Low	Low	High	Low	Low	High
15	Shiasi *et al* (2014)	Unclear	Unclear	High	High	High	Low	Low	High
16	Alisi *et al* (2014)	Low	Low	Low	Low	Low	Low	Low	Low
17	Ghergherehchi *et al* (2013)	Low	Unclear	Low	Low	Low	Low	Low	Unclear
18	Nobili *et al* (2011)	Low	Low	Low	Low	Low	Low	Low	Low
19	Akcam *et al* (2011)	Unclear	Unclear	Low	Low	Low	Low	Low	Unclear

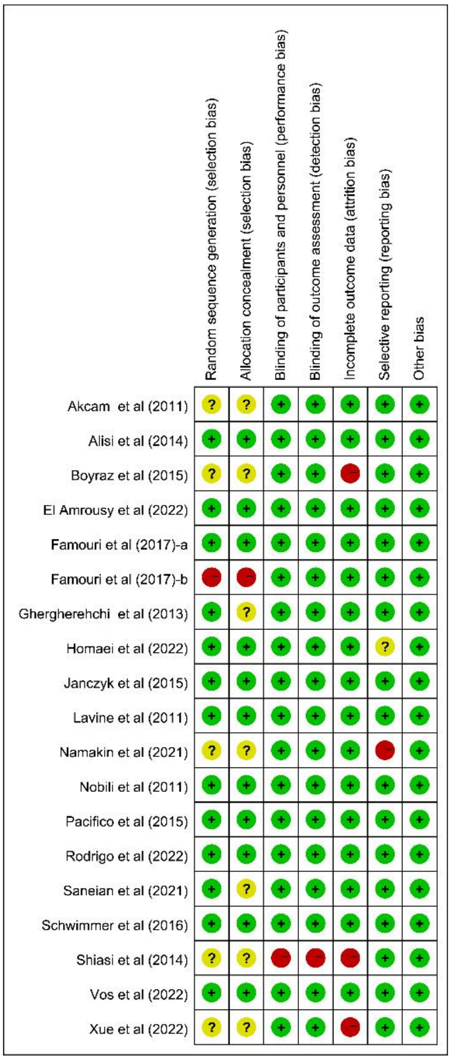

Figure S1. Risk of bias summary for the review authors' judgments about each risk-of-bias item for each included study

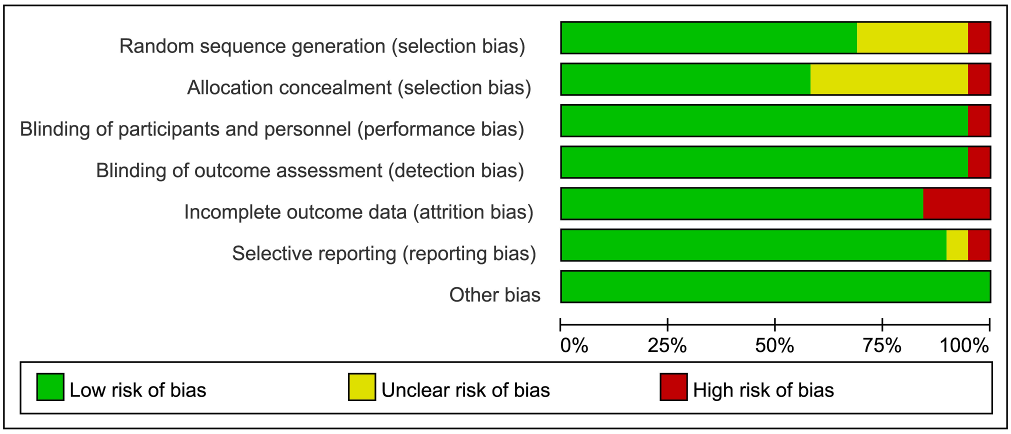

Figure S2. Risk of bias graph showing the review authors' judgments about each risk-of-bias item presented as percentages across all included studies

Supplementary File 4. Results of NMA of primary outcome

Table S1. Ranking probability of different pharmacological interventions for improving hepatic steatosis

Subset	1	2	3	4	5
Placebo	0.000	0.000	0.004	0.079	0.917†
Energy	0.095	0.325	0.440†	0.138	0.003
Inflammation	0.016	0.094	0.307	0.568†	0.015
Fibrosis	0.632†	0.128	0.074	0.107	0.059
Modify gut microbiota	0.257	0.453†	0.175	0.108	0.006

Note: † represents the maximum value of the ranking probability of the five interventions involved in the corresponding rank sequence. Where horizontal rows 1 to 5 represent the rank sequence, the values in the table represent the probability values of the corresponding interventions, with the highest probability value of the corresponding intervention ranking first when ranking = 1, and so forth.

Table S2. Summary of cumulative ranking probabilities and SUCRA values for different pharmacological interventions for improving hepatic steatosis

Subset	1	2	3	4	5	SUCRA (%)
Placebo	0.000	0.000	0.004	0.083	1.000	2.17%
Energy	0.095	0.419	0.859	0.997	1.000	59.29%
Inflammation	0.016	0.110	0.417	0.985	1.000	38.21%
Fibrosis	0.632	0.760	0.834	0.941	1.000	79.18%
Modify gut microbiota	0.257	0.711	0.886	0.994	1.000	71.15%

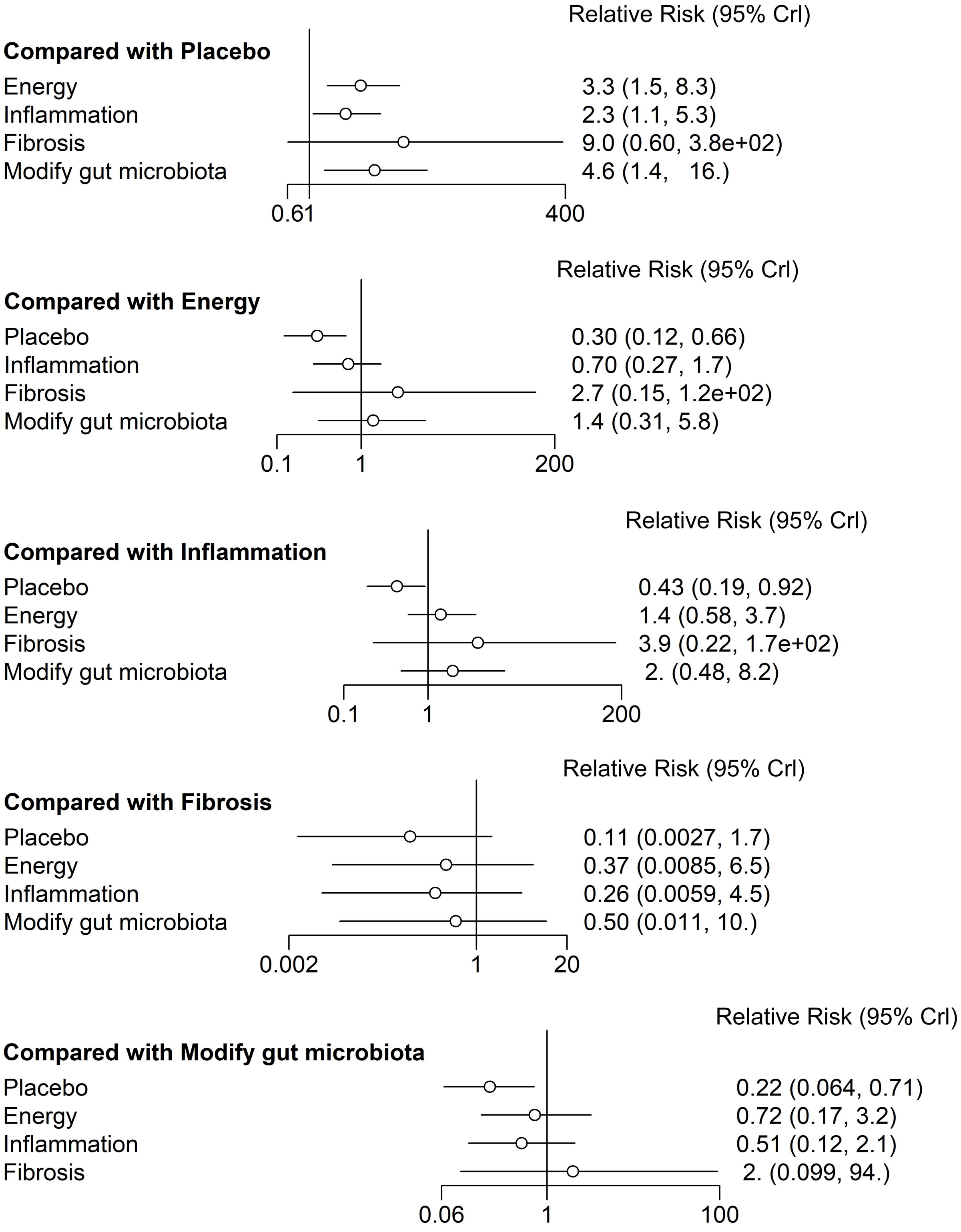

Figure S1. Network meta-analysis forest plot of different pharmacological interventions for improving hepatic steatosis

Supplementary File 5. Results of NMA of liver enzymes (ALT, AST)

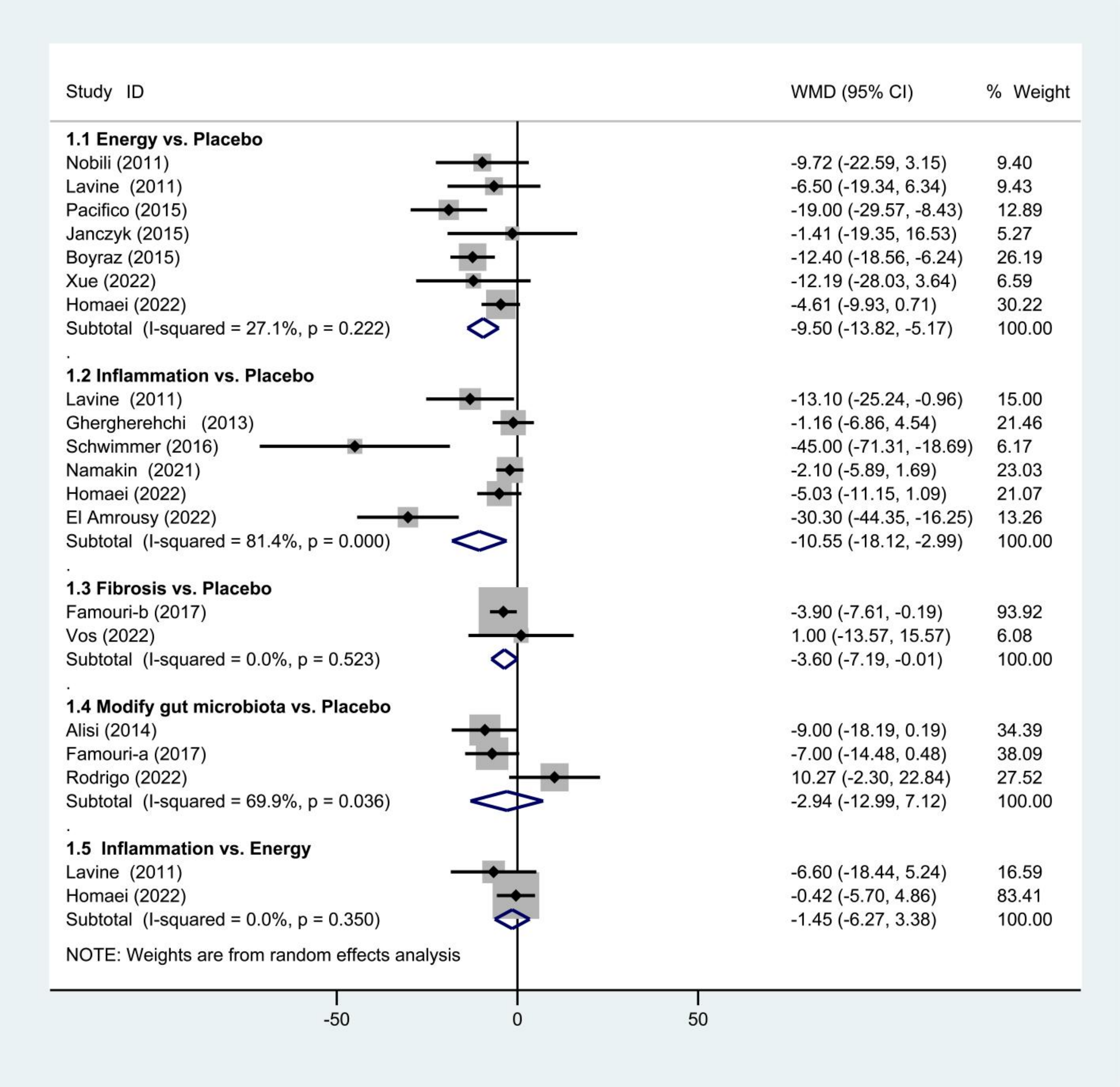

Figure S1. Direct meta-analysis of different pharmacological interventions for reducing in ALT levels.

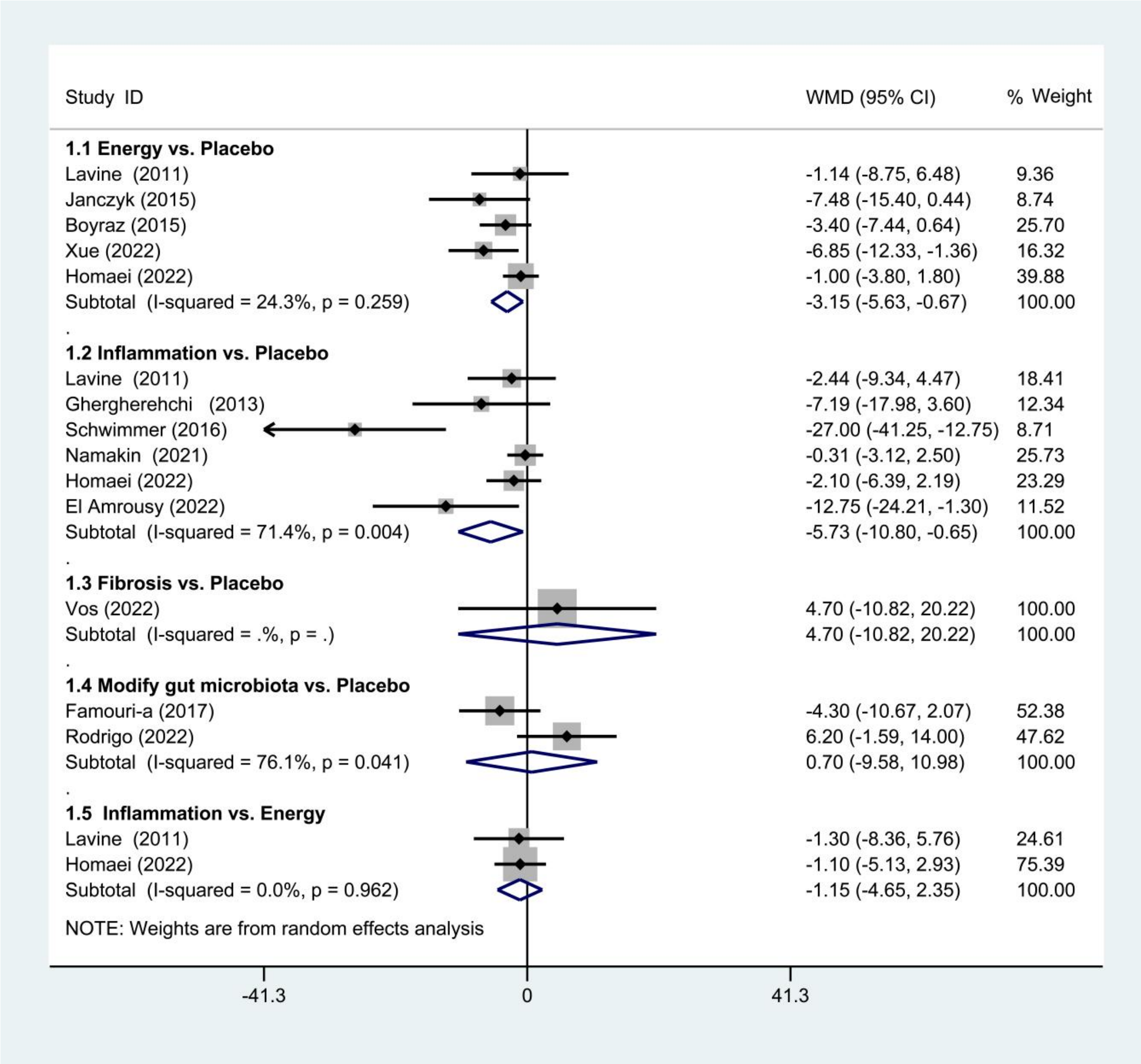

Figure S2. Direct meta-analysis of different pharmacological interventions for reducing in AST levels.

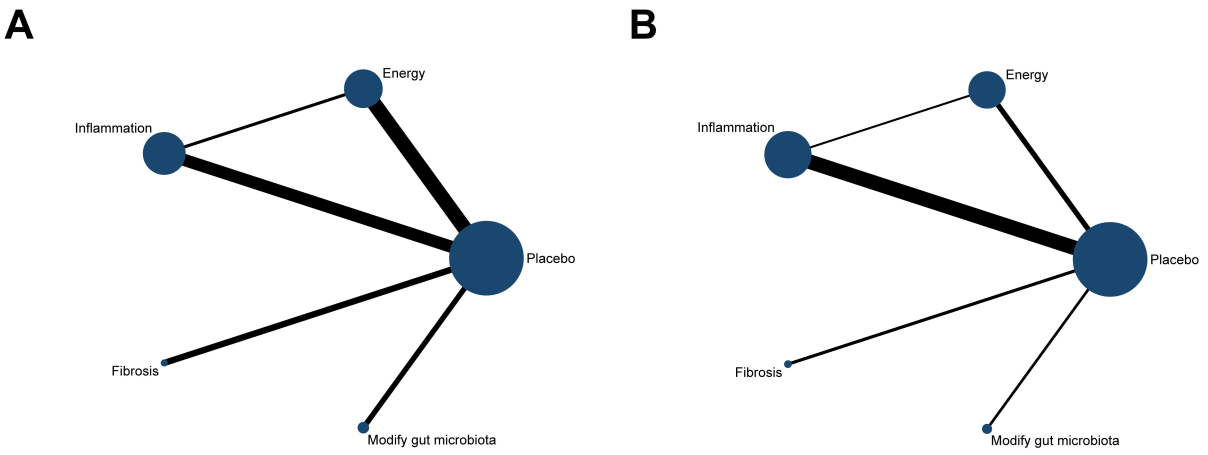

Figure S3. Network evidence map of reduction in liver enzymes. **(A)** ALT, **(B)** AST.

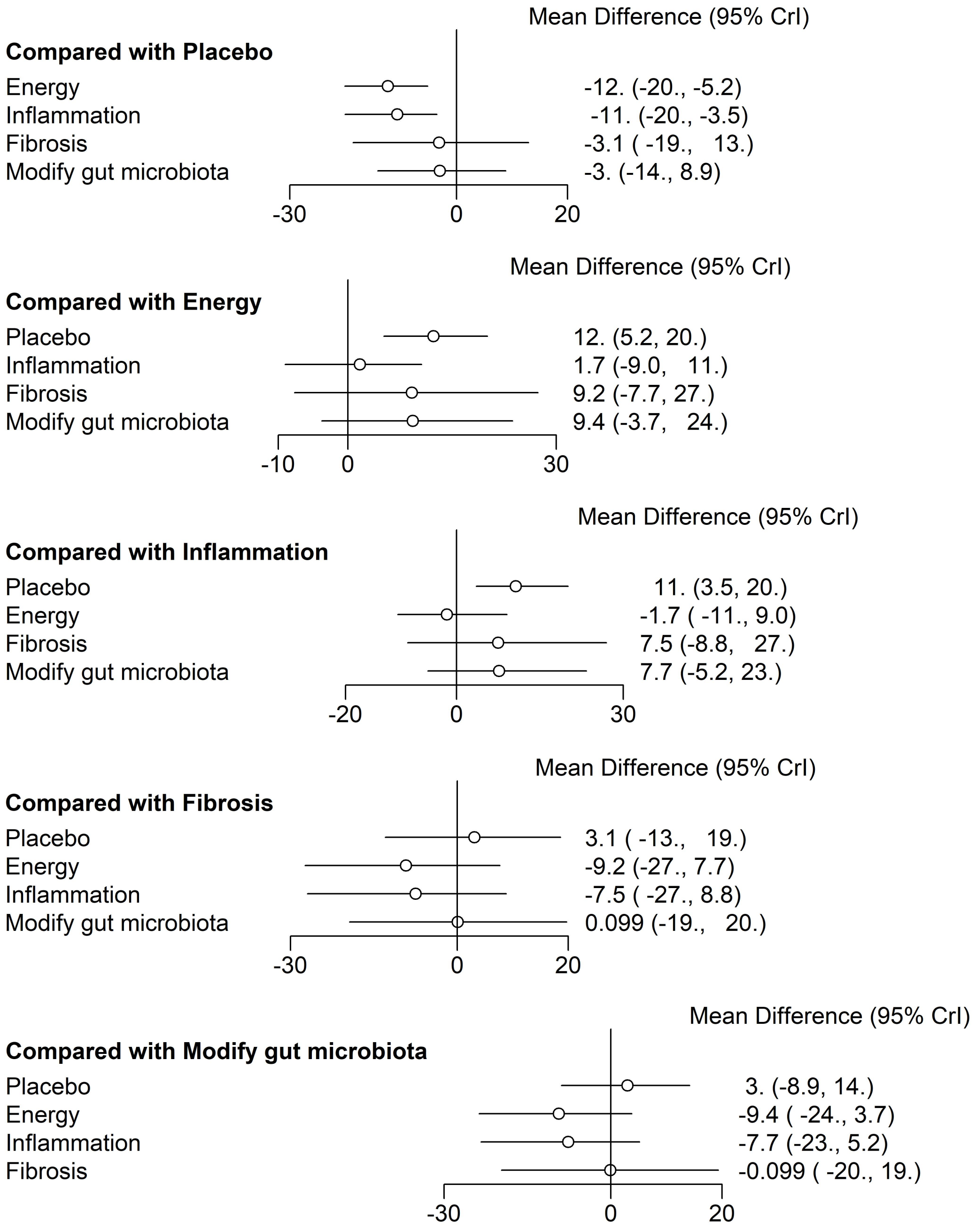

Figure S4. Network meta-analysis forest plot of different pharmacological interventions for reducing ALT levels.

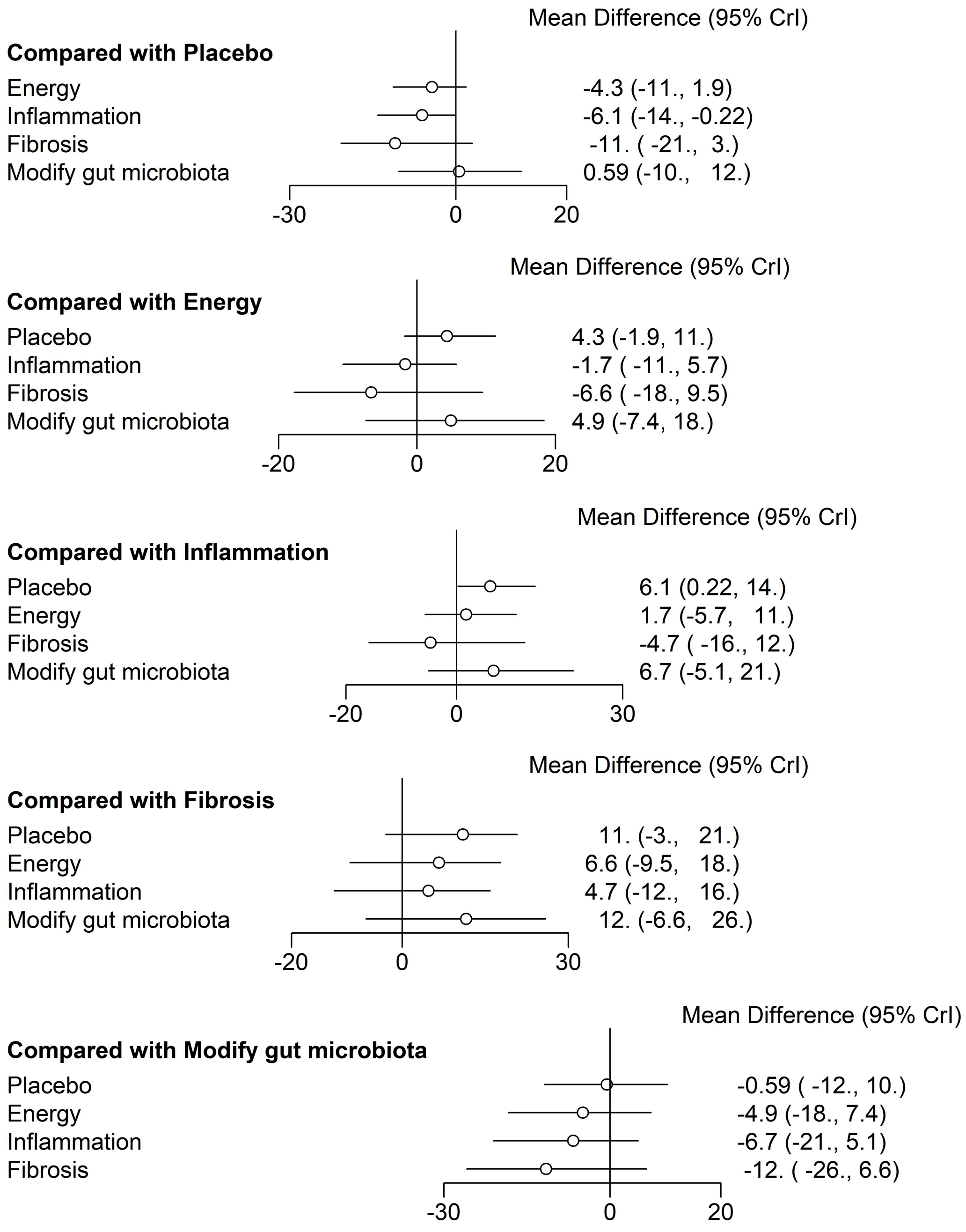

Figure S5. Network meta-analysis forest plot of different pharmacological interventions for reducing AST levels.

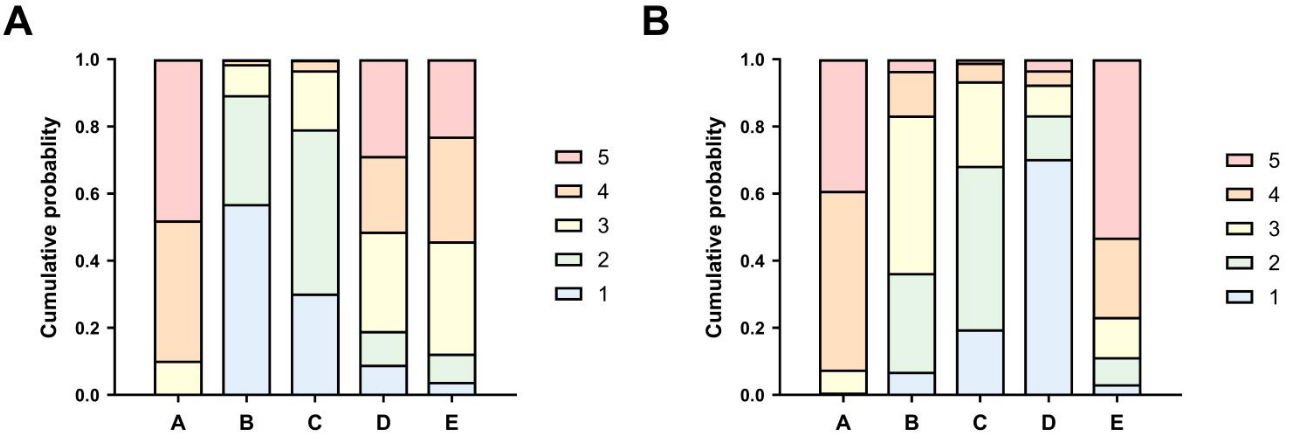

Figure S6. The cumulative ranking probability plot of reduction in liver enzymes. (A) ALT, (B) AST.

Note: A ~ E represent five different interventions, where A is placebo, B is an energy-modifying agent, C is an inflammation-modifying agent, D is a fibrosis-modifying agent, and E is a microecological agent);

Table S1. Ranking probability of different pharmacological interventions for reducing liver enzymes.

	1	2	3	4	5
**ALT**					
Placebo	0.000	0.001	0.102	0.418†	0.480
Energy	0.569†	0.325	0.092	0.013	0.001
Inflammation	0.302	0.490†	0.176	0.031	0.002
Fibrosis	0.090	0.101	0.296	0.225	0.288†
Modify gut microbiota	0.039	0.084	0.335†	0.312	0.230
**AST**					
Placebo	0.000	0.007	0.068	0.533†	0.392
Energy	0.069	0.294	0.469†	0.133	0.035
Inflammation	0.196	0.487†	0.252	0.055	0.010
Fibrosis	0.703†	0.130	0.092	0.042	0.033
Modify gut microbiota	0.032	0.081	0.119	0.237	0.531†

Note: † represents the maximum value of the ranking probability of the five interventions involved in the corresponding rank sequence. Where horizontal rows 1 to 5 represent the rank sequence, the values in the table represent the probability values of the corresponding interventions, with the highest probability value of the corresponding intervention ranking first when ranking = 1, and so forth.

Table S2. Summary of cumulative ranking probabilities and SUCRA values for different pharmacological interventions for reducing liver enzymes.

	1	2	3	4	5	SUCRA (%)
**ALT**						
Placebo	0.000	0.001	0.102	0.520	1.000	15.59%
Energy	0.569	0.894	0.986	0.999	1.000	86.20%
Inflammation	0.302	0.792	0.967	0.998	1.000	76.49%
Fibrosis	0.090	0.191	0.487	0.712	1.000	36.98%
Modify gut microbiota	0.039	0.123	0.458	0.770	1.000	34.74%
**AST**						
Placebo	0.000	0.007	0.075	0.608	1.000	17.29%
Energy	0.069	0.363	0.833	0.965	1.000	55.75%
Inflammation	0.196	0.683	0.935	0.990	1.000	70.07%
Fibrosis	0.703	0.833	0.925	0.967	1.000	85.72%
Modify gut microbiota	0.032	0.113	0.232	0.469	1.000	21.17%

Supplementary File 6. Results of NMA of lipid profile (TC, TG, HDL-C, LDL-C)

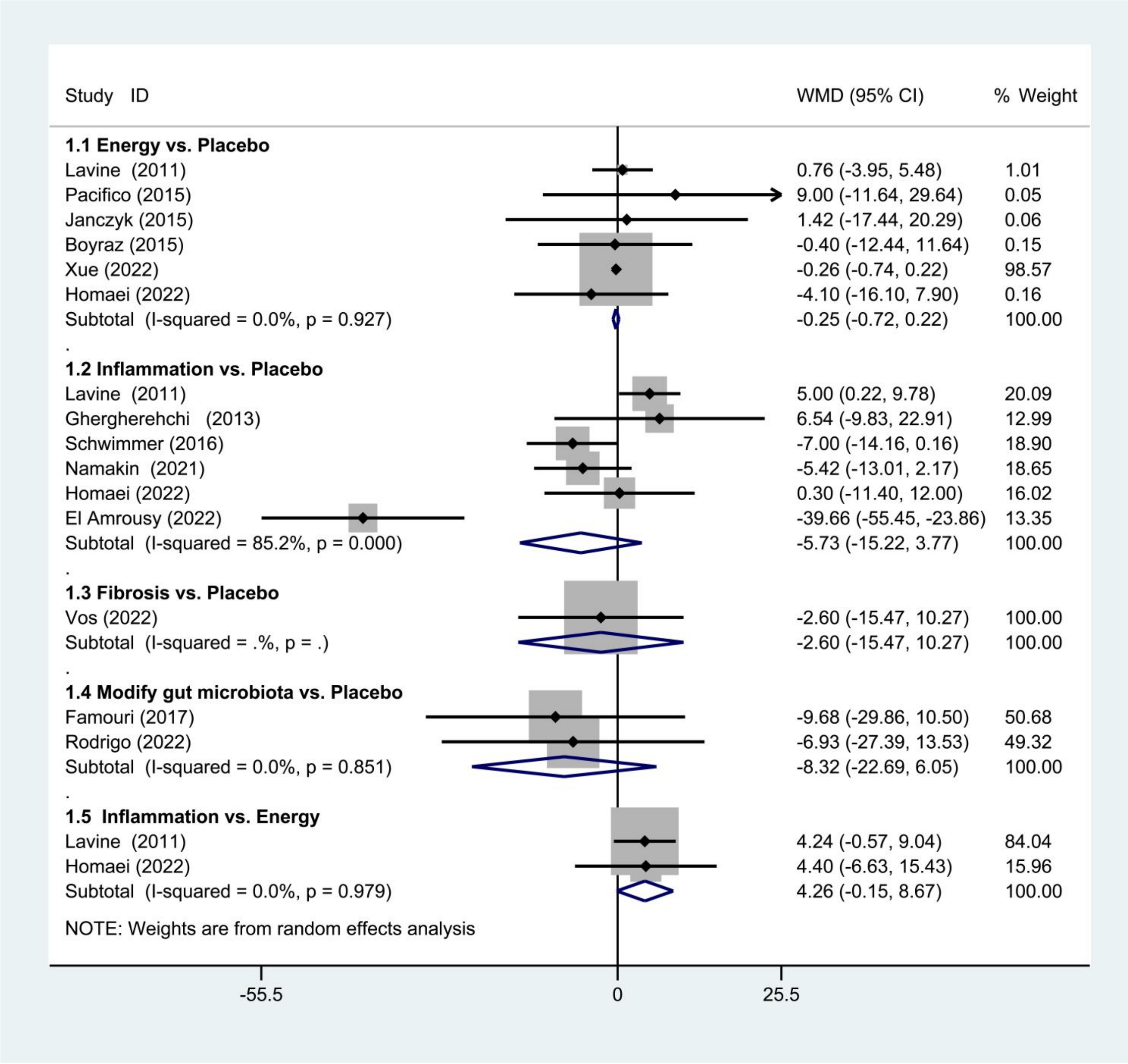

Figure S1. Direct meta-analysis of different pharmacological interventions for reducing TC levels.

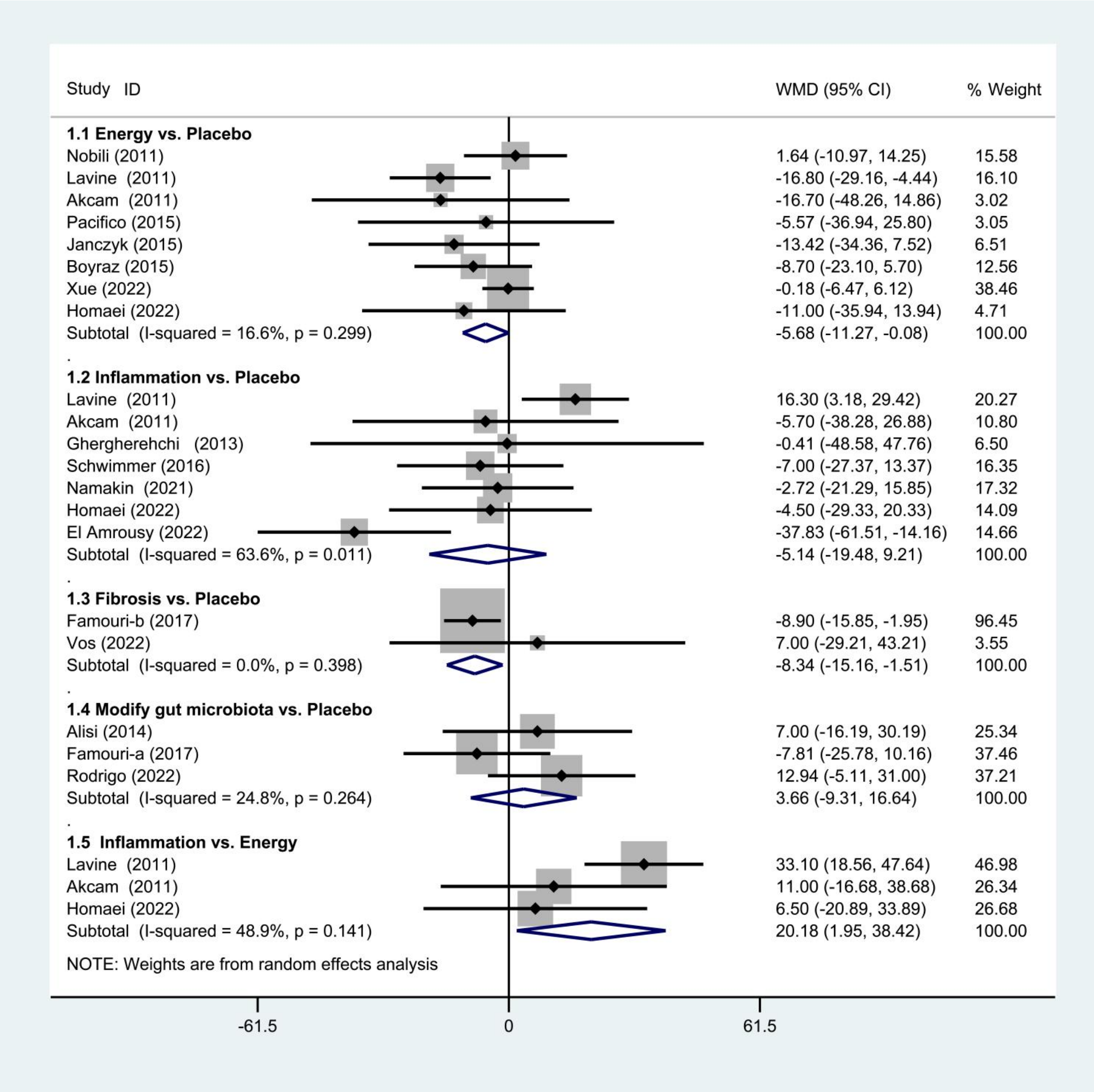

Figure S2. Direct meta-analysis of different pharmacological interventions for reducing TG levels.

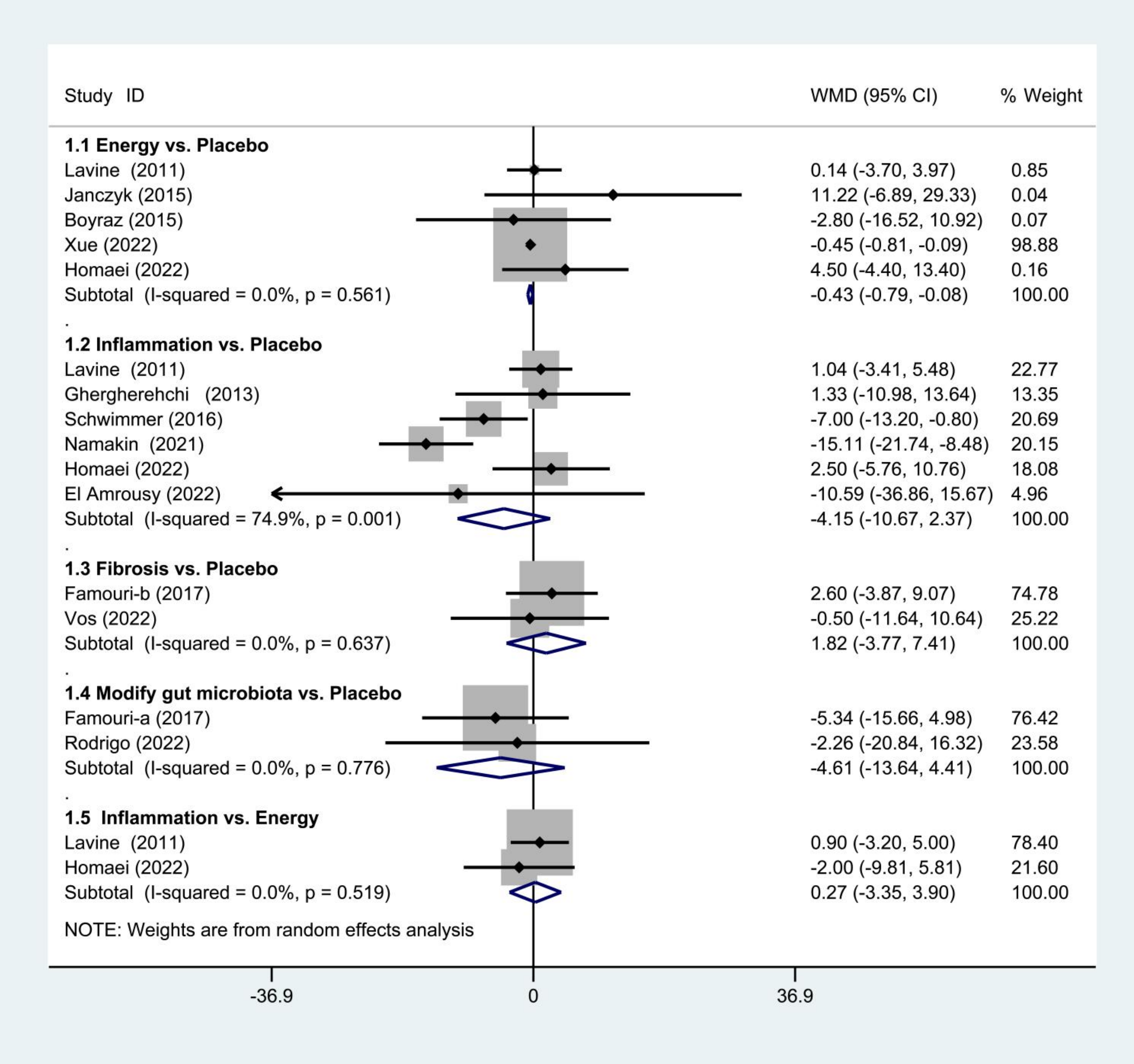

Figure S3. Direct meta-analysis of different pharmacological interventions for reducing LDL-C levels.

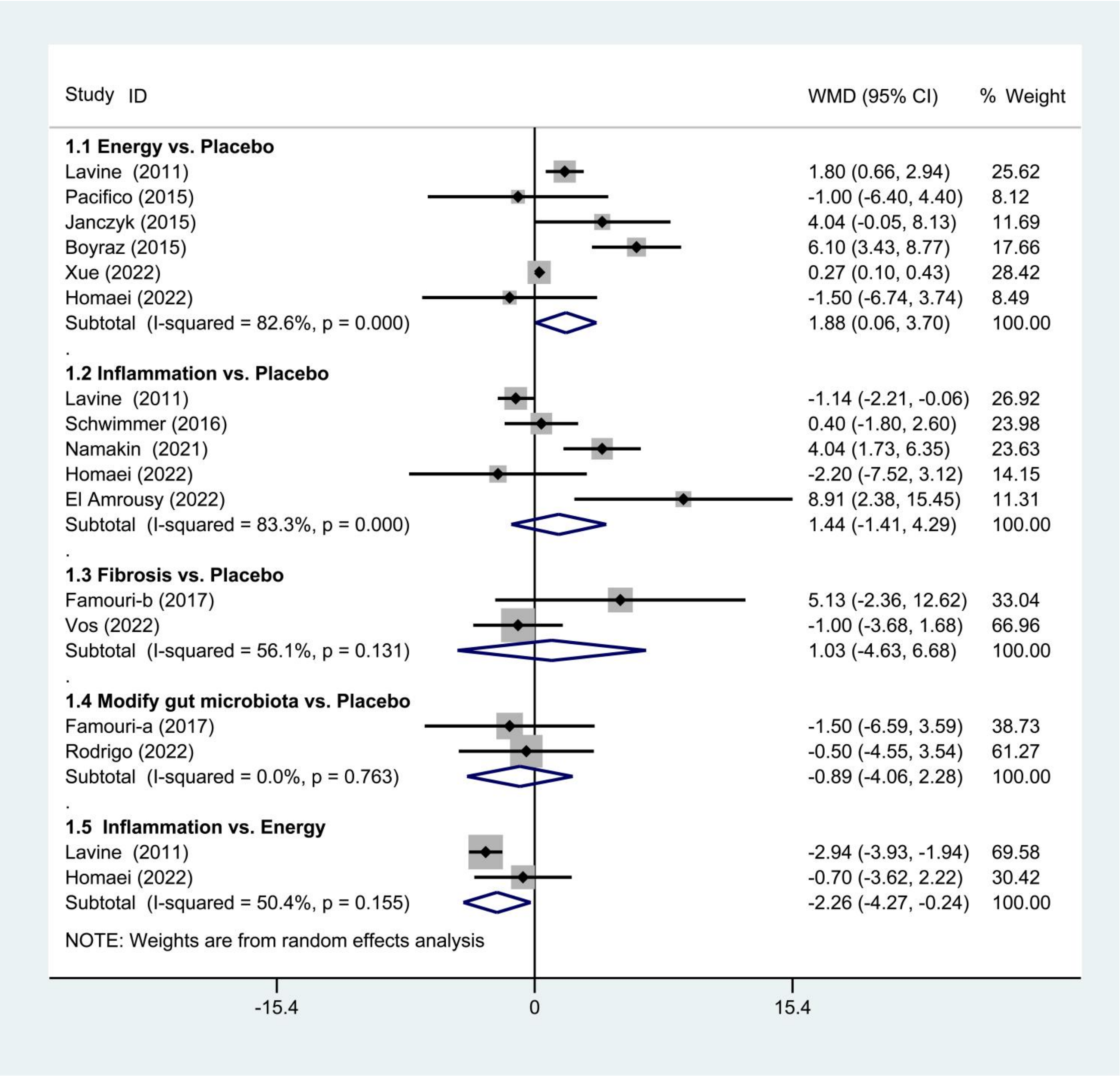

Figure S4. Direct meta-analysis of different pharmacological interventions to improve HDL-C levels.

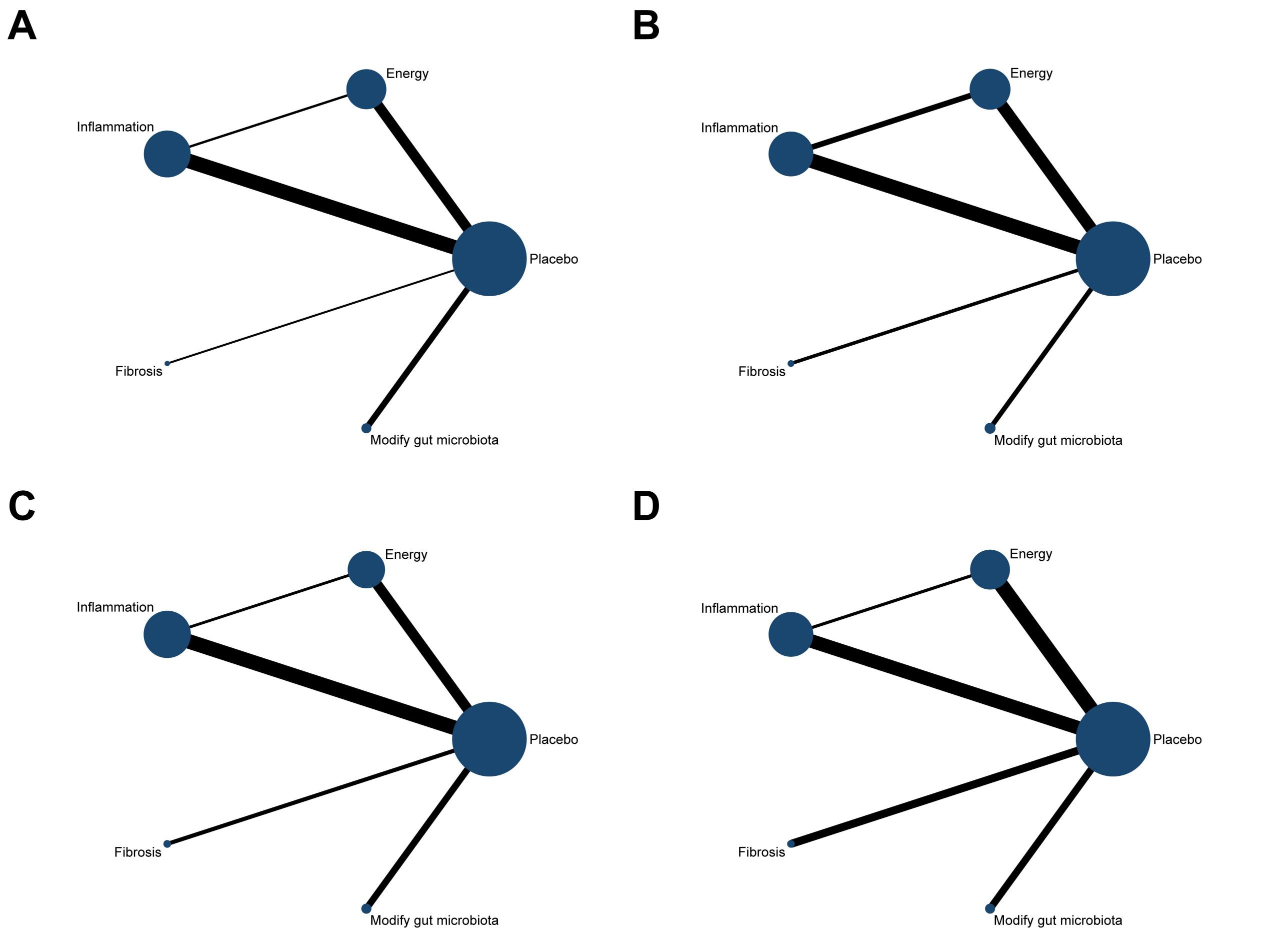

Figure S5. Network evidence plot of improvement in lipid profiles. (A) TC, (B) TG, (C) LDL-C, (D) HDL-C.

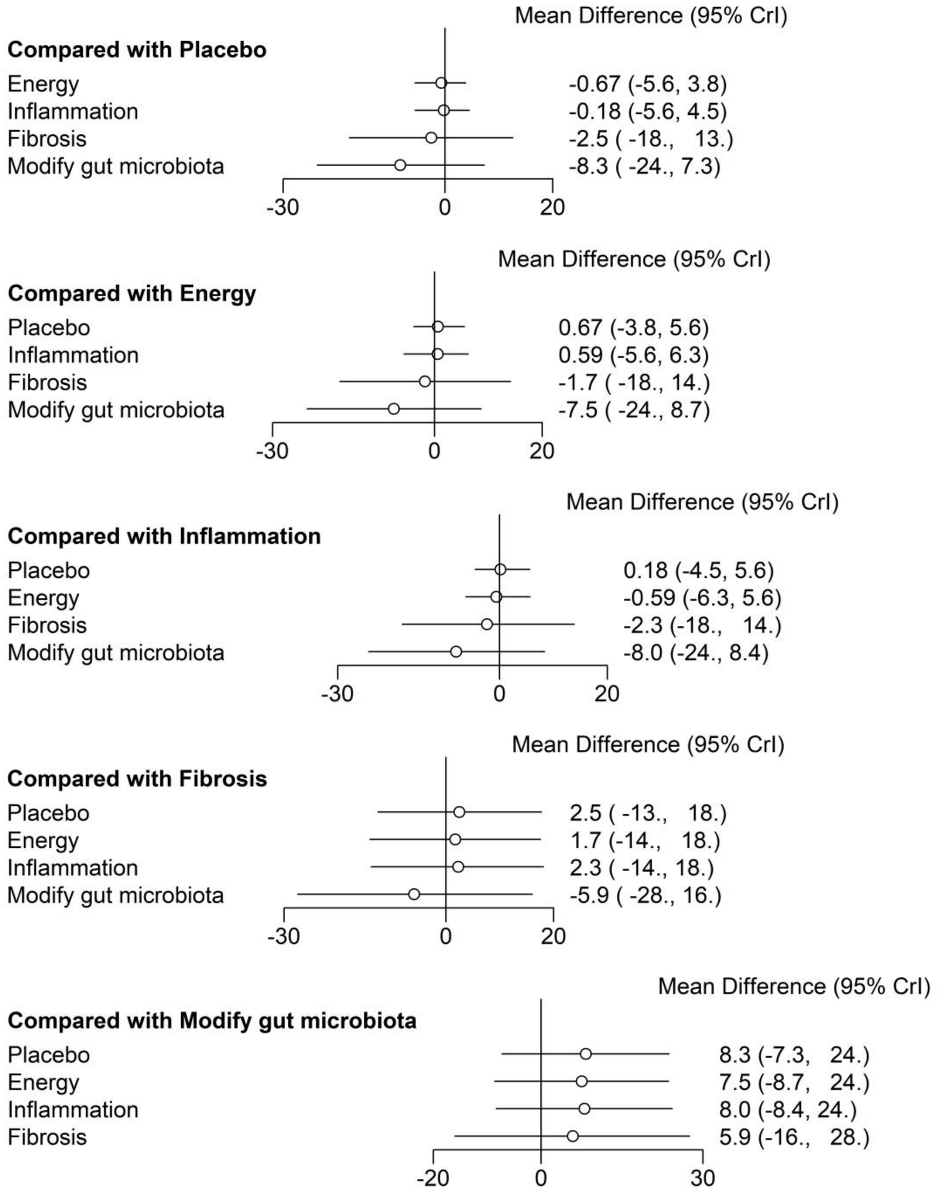

Figure S6. Network meta-analysis forest plot of different pharmacological interventions for reducing TC levels.

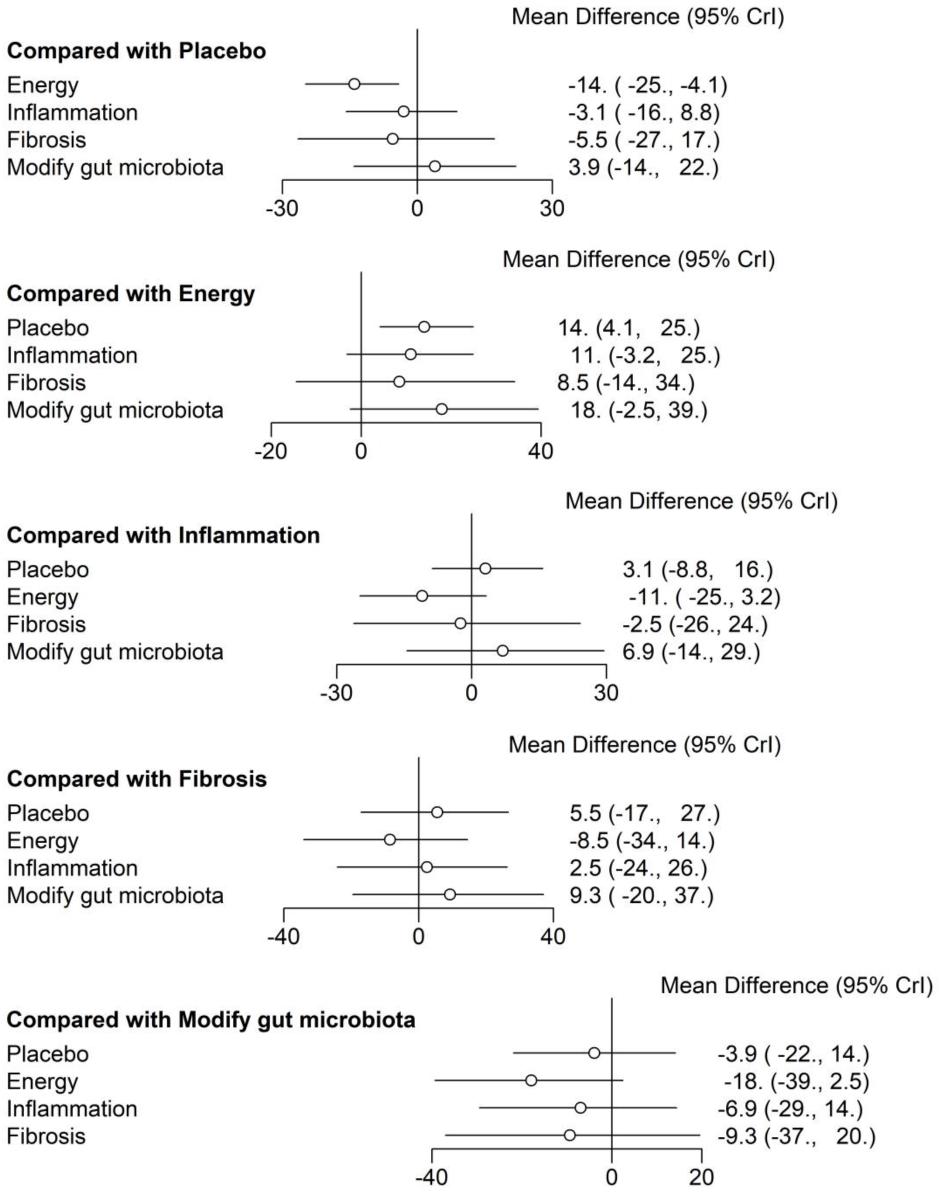

Figure S7. Network meta-analysis forest plot of different pharmacological interventions for reducing TG levels.

frontiers

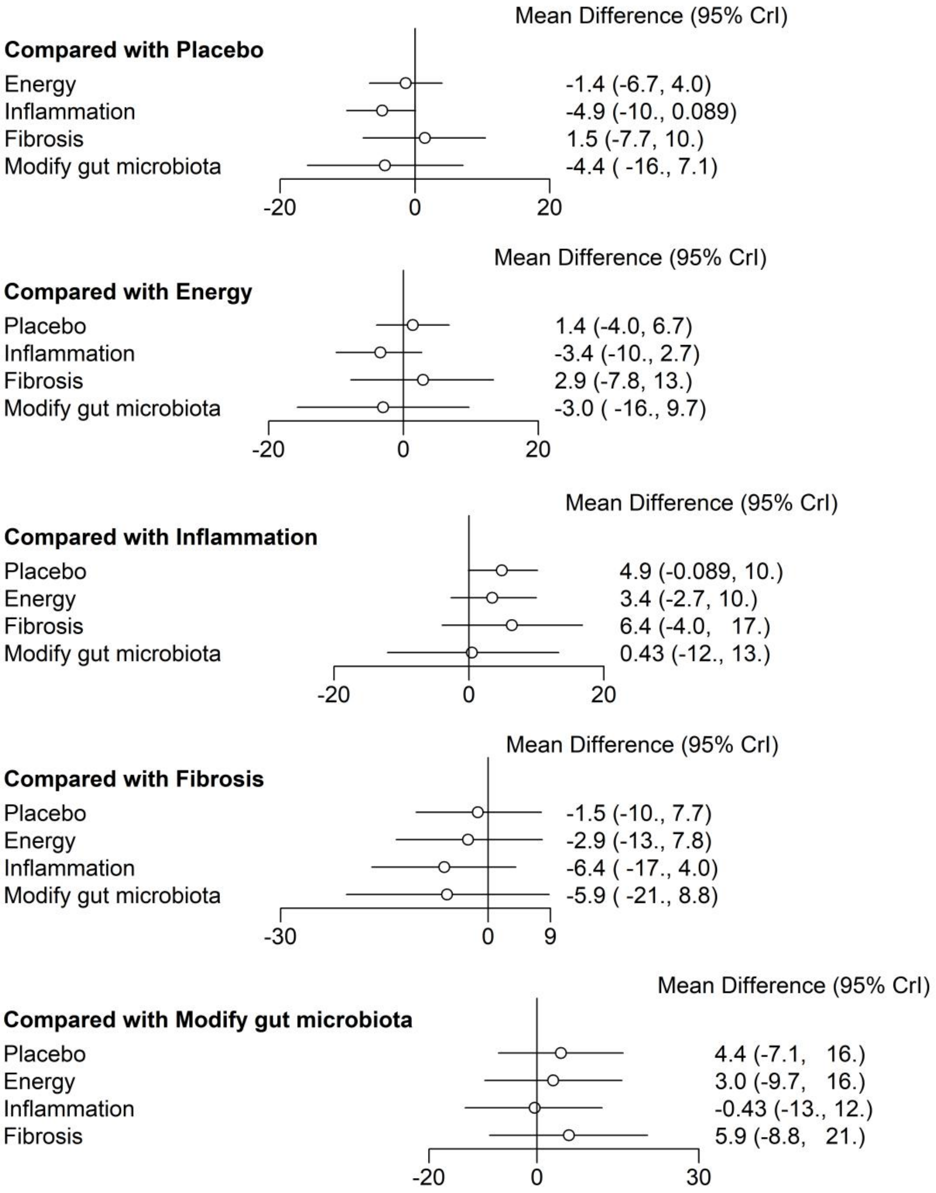

Figure S8. Network meta-analysis forest plot of different pharmacological interventions for reducing LDL-C levels.

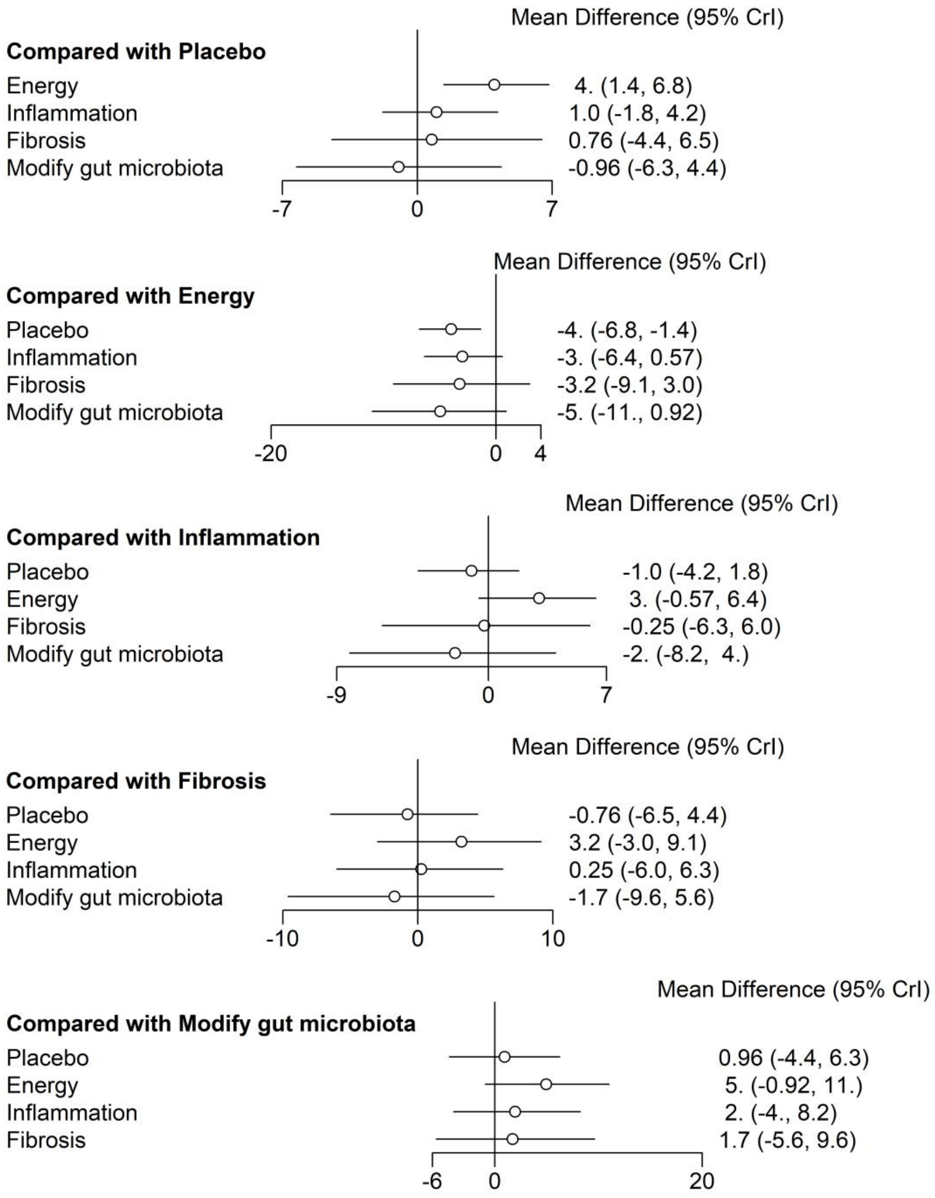

Figure S9. Network meta-analysis forest plot of different pharmacological interventions to improve HDL-C levels.

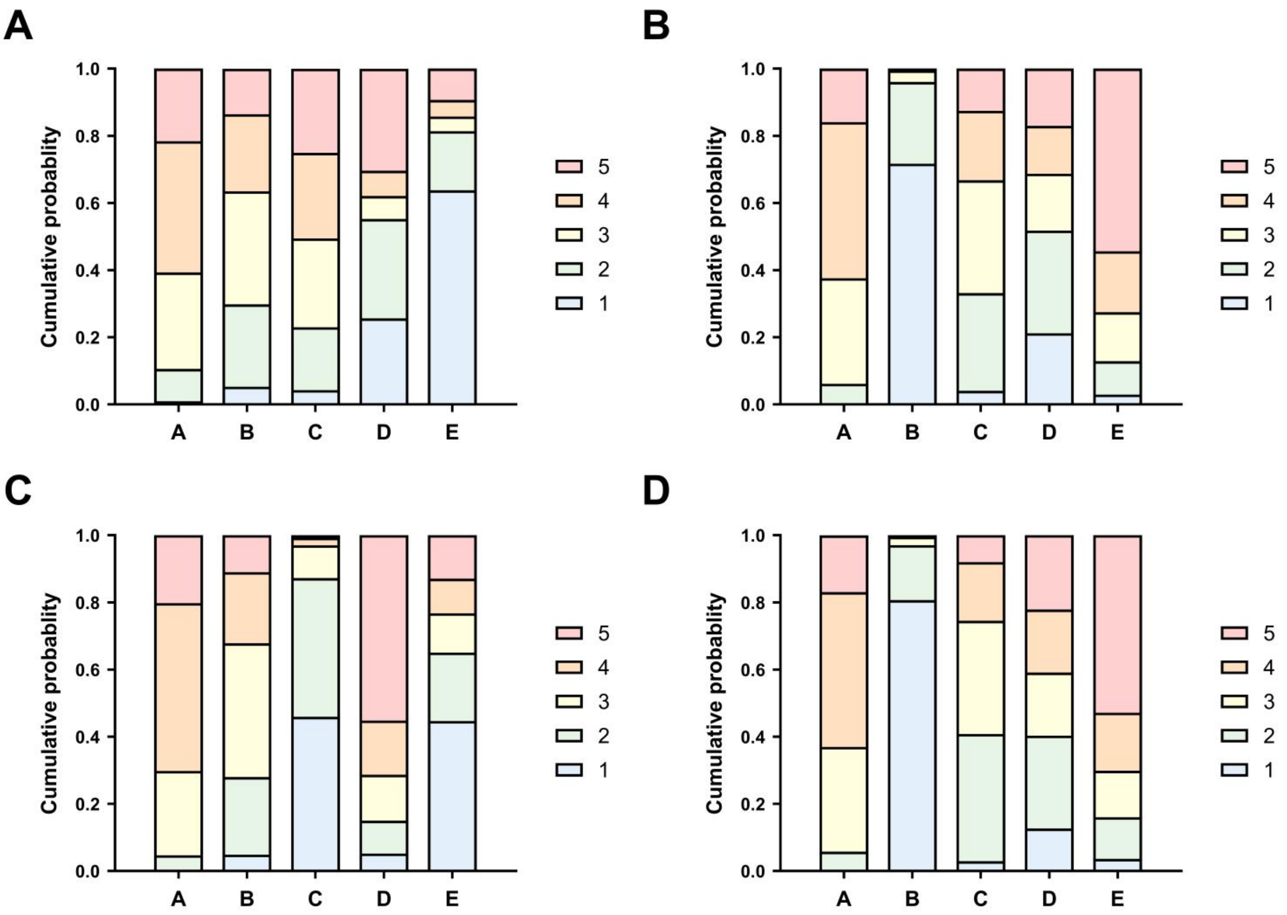

Figure S10. The cumulative ranking probability plot for improvement in lipid profiles. (A) TC, (B) TG, (C) LDL-C, (D) HDL-C.

Note: A ~ E represent five different interventions, where A is placebo, B is an energy-modifying agent, C is an inflammation-modifying agent, D is a fibrosis-modifying agent, and E is a microecological agent);

Table S1. Ranking probability of different pharmacological interventions for improvement in lipid profiles.

	1	2	3	4	5
**TC**					
Placebo	0.010	0.096	0.287	0.391†	0.216
Energy	0.053	0.245	0.336†	0.230	0.136
Inflammation	0.043	0.187	0.264	0.255	0.251†
Fibrosis	0.256	0.296†	0.069	0.075	0.304
Modify gut microbiota	0.638†	0.176	0.044	0.049	0.093
**TG**					
Placebo	0.000	0.061	0.314	0.465†	0.159
Energy	0.717†	0.243	0.034	0.005	0.001
Inflammation	0.041	0.291	0.336†	0.207	0.126
Fibrosis	0.212	0.305†	0.169	0.142	0.170
Modify gut microbiota	0.030	0.099	0.146	0.181	0.544†
**LDL-C**					
Placebo	0.002	0.045	0.252	0.500†	0.202
Energy	0.049	0.231†	0.398†	0.212	0.110
Inflammation	0.460†	0.413	0.097	0.023	0.007
Fibrosis	0.052	0.098	0.136	0.161	0.552†
Modify gut microbiota	0.447	0.204	0.117	0.103	0.129
**HDL-C**					
Placebo	0.000	0.057	0.312†	0.461	0.169
Energy	0.807†	0.164	0.025	0.004	0.001
Inflammation	0.030	0.379†	0.337	0.174	0.080
Fibrosis	0.127	0.276	0.188	0.188†	0.221
Modify gut microbiota	0.036	0.124	0.139	0.173	0.529†

Note: † represents the maximum value of the ranking probability of the five interventions involved in the corresponding rank sequence. Where horizontal rows 1 to 5 represent the rank sequence, the values in the table represent the probability values of the corresponding interventions, with the highest probability value of the corresponding intervention ranking first when ranking = 1, and so forth.

Table S2. Summary of cumulative ranking probabilities and SUCRA values for different pharmacological interventions for improvement in lipid profiles.

	1	2	3	4	5	SUCRA (%)
**TC**						
Placebo	0.010	0.106	0.393	0.784	1.000	32.33%
Energy	0.053	0.298	0.634	0.864	1.000	46.24%
Inflammation	0.043	0.230	0.494	0.749	1.000	37.91%
Fibrosis	0.256	0.552	0.621	0.696	1.000	53.14%
Modify gut microbiota	0.638	0.814	0.857	0.907	1.000	80.38%
**TG**						
Placebo	0.000	0.062	0.376	0.841	1.000	31.96%
Energy	0.717	0.960	0.995	0.999	1.000	91.77%
Inflammation	0.041	0.332	0.667	0.874	1.000	47.85%
Fibrosis	0.212	0.518	0.687	0.830	1.000	56.17%
Modify gut microbiota	0.030	0.129	0.275	0.456	1.000	22.23%
**LDL-C**						
Placebo	0.002	0.047	0.298	0.798	1.000	28.64%
Energy	0.049	0.280	0.678	0.890	1.000	47.44%
Inflammation	0.460	0.872	0.970	0.993	1.000	82.37%
Fibrosis	0.052	0.150	0.287	0.448	1.000	23.42%
Modify gut microbiota	0.447	0.651	0.767	0.871	1.000	68.38%
**HDL-C**						
Placebo	0.000	0.058	0.370	0.831	1.000	31.46%
Energy	0.807	0.970	0.995	0.999	1.000	94.29%
Inflammation	0.030	0.409	0.745	0.920	1.000	52.59%
Fibrosis	0.127	0.403	0.591	0.779	1.000	47.51%
Modify gut microbiota	0.036	0.160	0.299	0.471	1.000	24.16%

Supplementary File 7. Results of NMA of metabolic parameters (BMI, HOMA-IR)

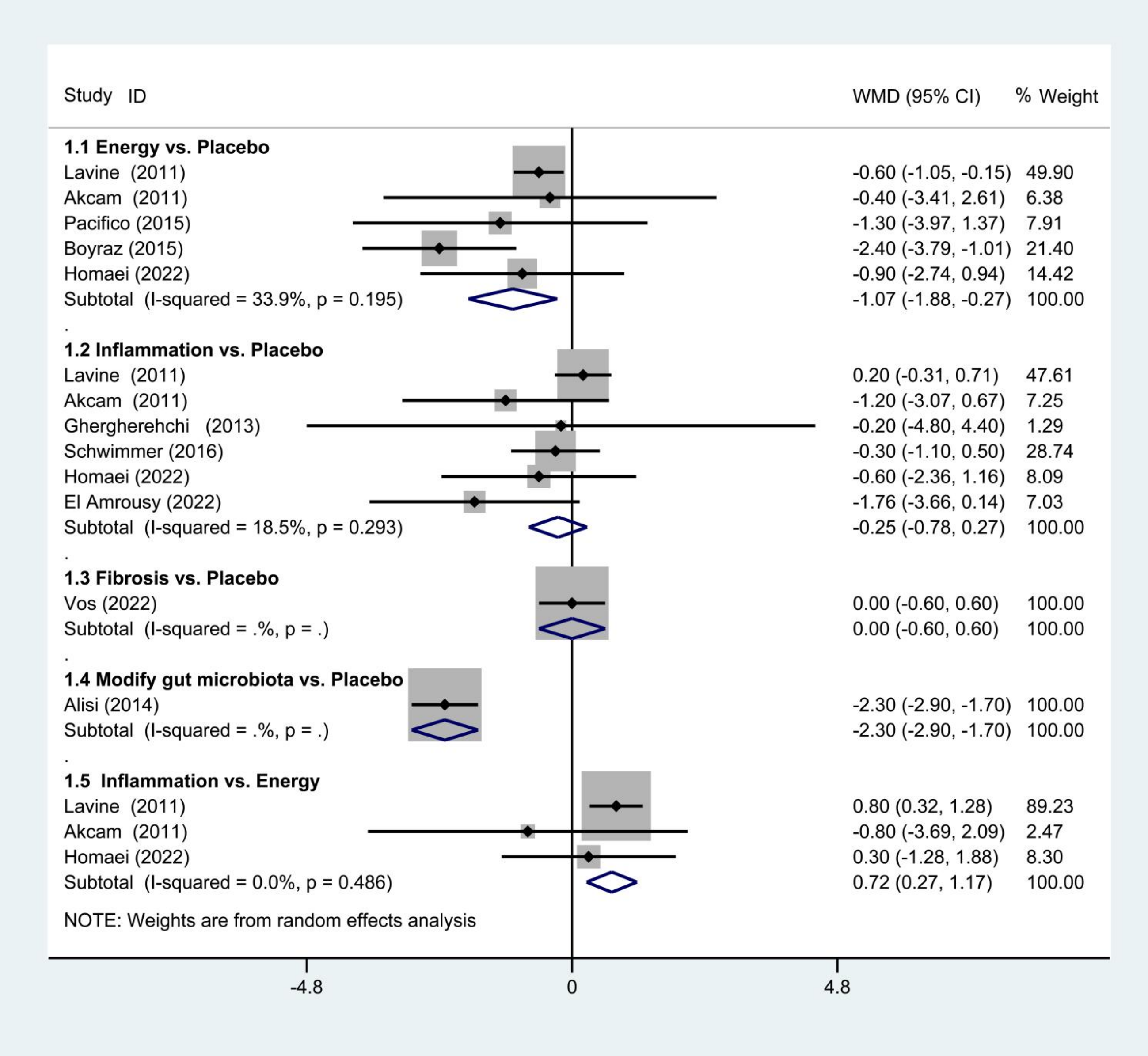

Figure S1. Direct meta-analysis of different pharmacological interventions for reducing BMI values.

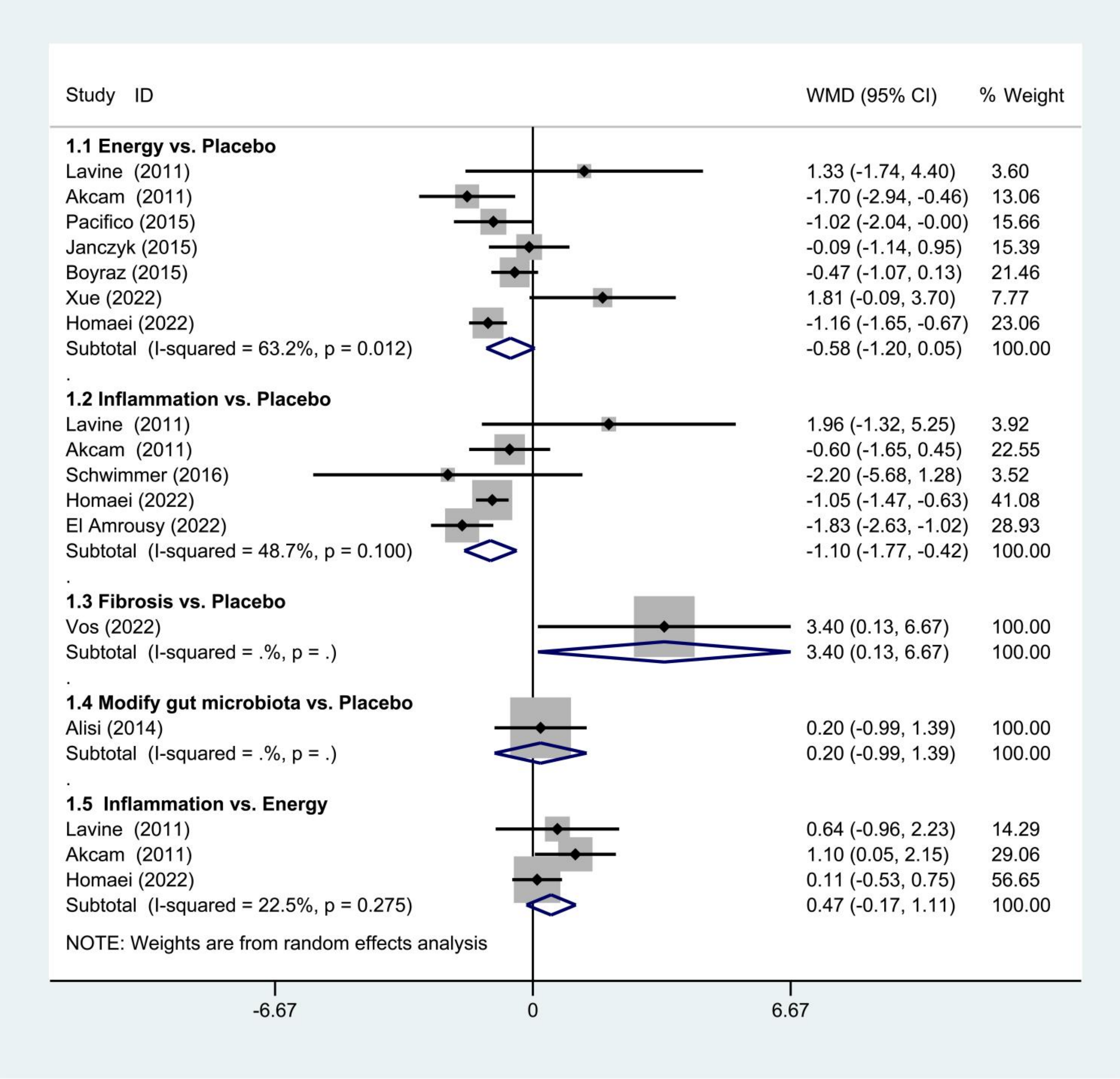

Figure S2. Direct meta-analysis of different pharmacological interventions for reducing HOMA-IR values.

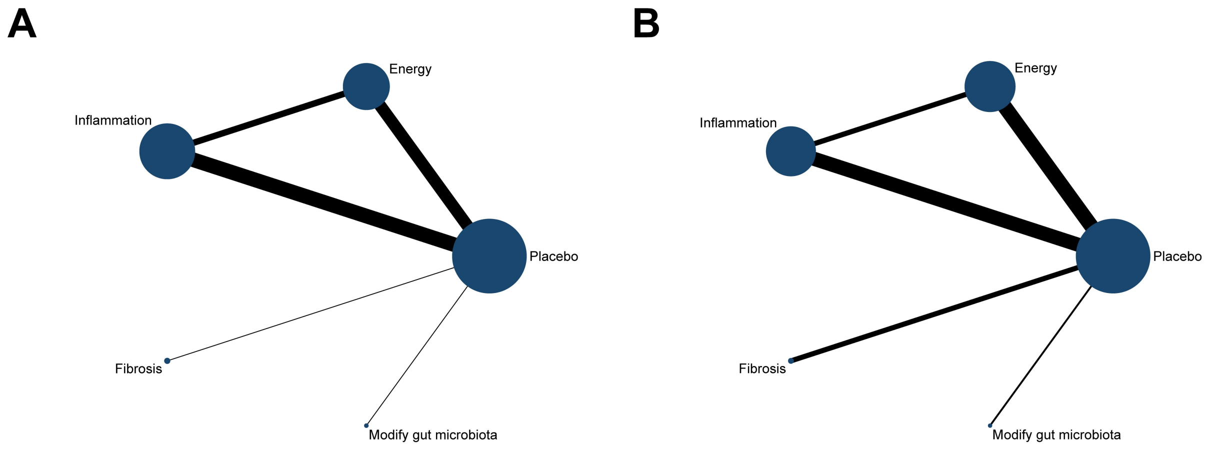

Figure S3. Network evidence plot for improvement in metabolic indicators. (A) BMI, (B) HOMA-IR.

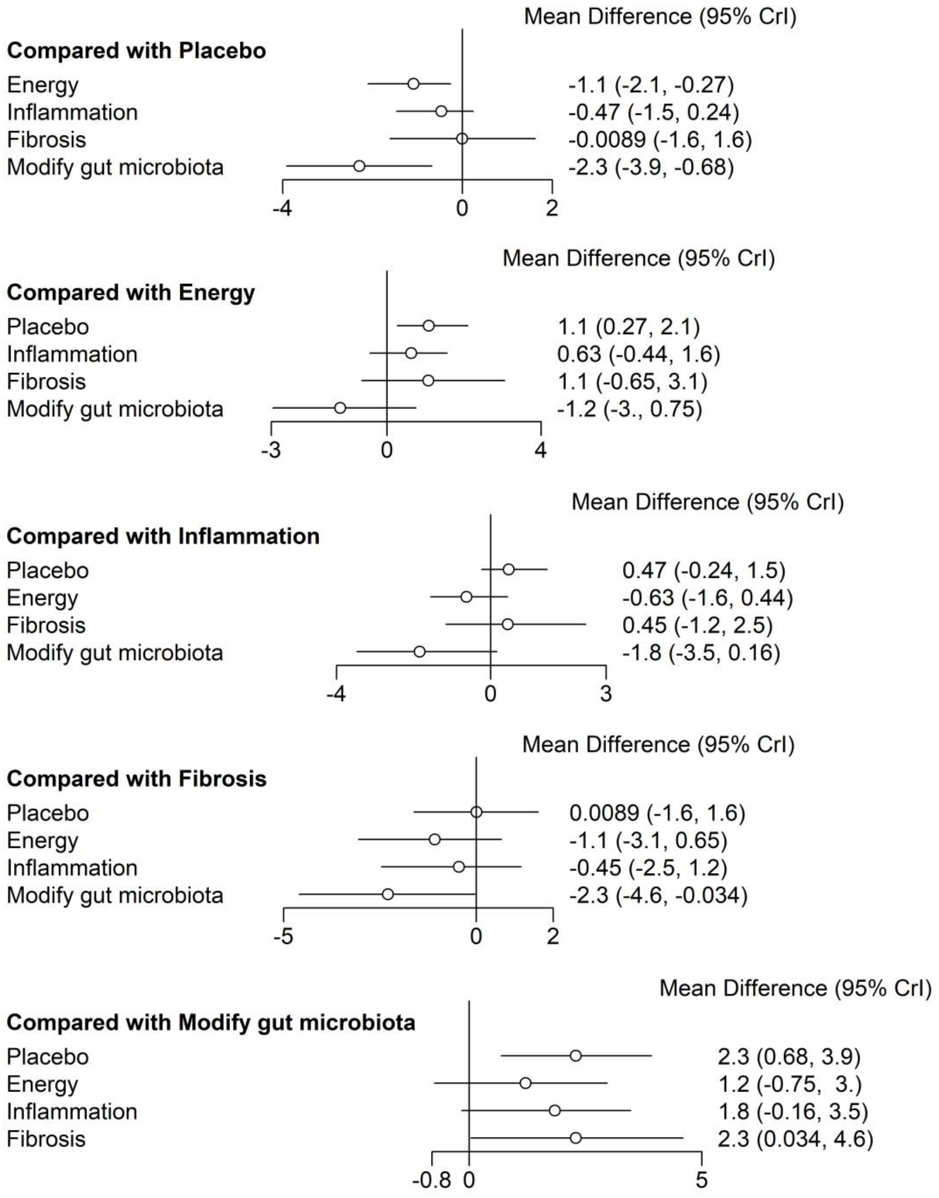

Figure S4. Network meta-analysis forest plot of different pharmacological interventions for reducing BMI values.

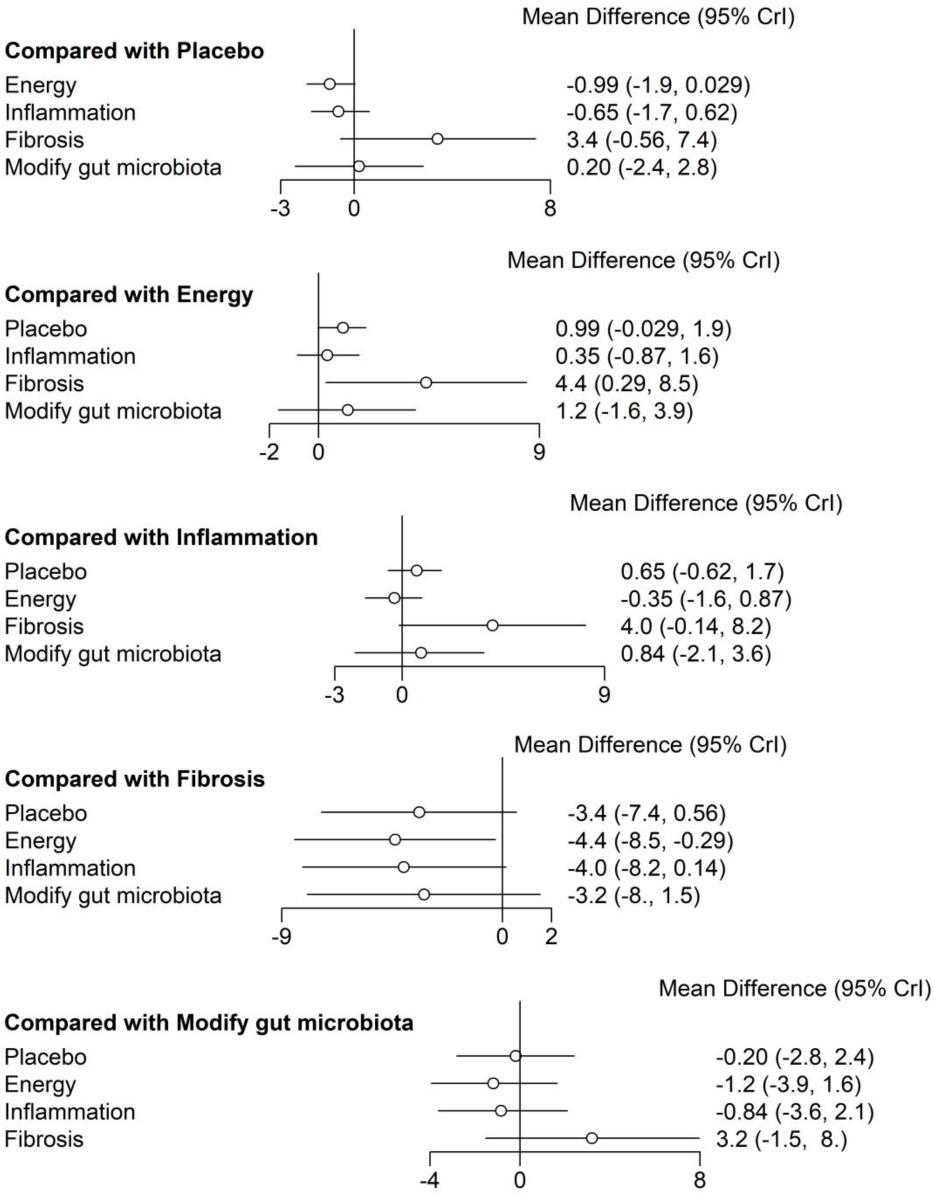

Figure S5. Network meta-analysis forest plot of different pharmacological interventions reducing HOMA-IR values.

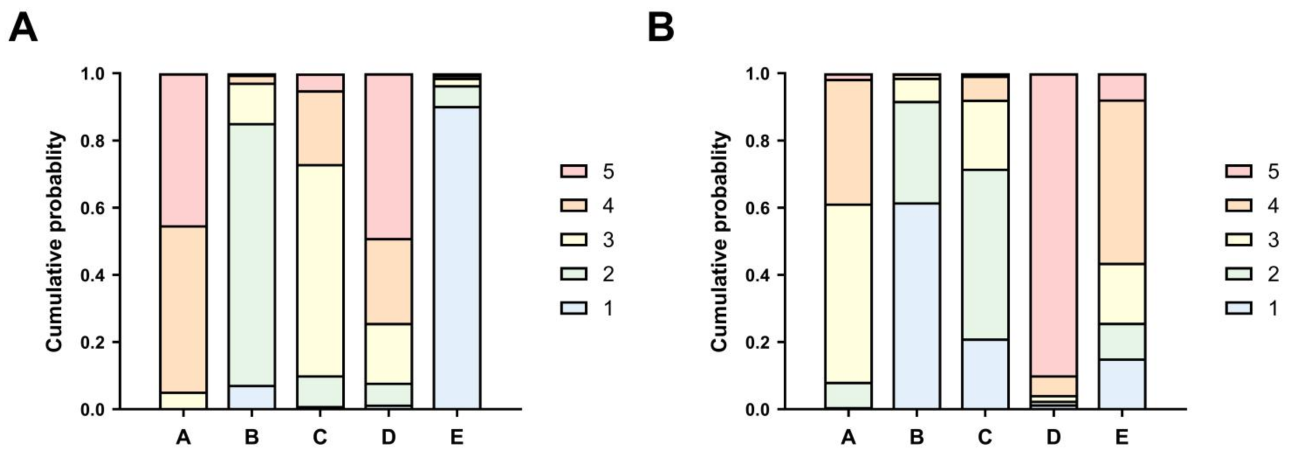

Figure S6. The cumulative ranking probability plot for improvement in metabolic indicators. (A) BMI, (B) HOMA-IR.

Note: A ~ E represent five different interventions, where A is placebo, B is an energy-modifying agent, C is an inflammation-modifying agent, D is a fibrosis-modifying agent, and E is a microecological agent);

Table S1. Ranking probability of different pharmacological interventions for improvement in metabolic indicators.

	1	2	3	4	5
**BMI**					
Placebo	0.000	0.002	0.051	0.496†	0.452
Energy	0.073	0.780†	0.120	0.024	0.004
Inflammation	0.010	0.091	0.629†	0.219	0.050
Fibrosis	0.014	0.065	0.178	0.253	0.490†
Modify gut microbiota	0.903†	0.062	0.022	0.008	0.005
**HOMA-IR**					
Placebo	0.007	0.075	0.530†	0.372	0.016
Energy	0.617†	0.301	0.069	0.012	0.001
Inflammation	0.211	0.506†	0.205	0.072	0.006
Fibrosis	0.015	0.011	0.017	0.058	0.899†
Modify gut microbiota	0.151	0.107	0.178	0.486†	0.078

Note: † represents the maximum value of the ranking probability of the five interventions involved in the corresponding rank sequence. Where horizontal rows 1 to 5 represent the rank sequence, the values in the table represent the probability values of the corresponding interventions, with the highest probability value of the corresponding intervention ranking first when ranking = 1, and so forth.

Table S2. Summary of cumulative ranking probabilities and SUCRA values for different pharmacological interventions for improvement in metabolic indicators.

	1	2	3	4	5	SUCRA (%)
**BMI**						
Placebo	0.000	0.002	0.053	0.548	1.000	15.07%
Energy	0.073	0.853	0.972	0.996	1.000	72.35%
Inflammation	0.010	0.101	0.730	0.950	1.000	44.79%
Fibrosis	0.014	0.079	0.257	0.510	1.000	21.53%
Modify gut microbiota	0.903	0.965	0.987	0.995	1.000	96.25%
**HOMA-IR**						
Placebo	0.007	0.082	0.612	0.984	1.000	42.13%
Energy	0.617	0.918	0.987	0.999	1.000	88.01%
Inflammation	0.211	0.716	0.922	0.994	1.000	71.05%
Fibrosis	0.015	0.026	0.043	0.101	1.000	4.62%
Modify gut microbiota	0.151	0.258	0.436	0.922	1.000	44.19%

Supplementary File 8. Pooled summary estimates derived from direct and network meta-analysis

Table S1. Pooled summary estimates derived from direct and network meta-analysis informing on the comparative efficacy of pharmacological strategies for improving hepatic steatosis in pediatric NAFLD patients.

Comparisons	Meta-analysis	
	Direct	Network
Primary outcome: an improvement in hepatic steatosis		
Energy *vs.* Placebo	**3.11 (1.48, 6.53) ***	**3.32 (1.52, 8.26) ***
Inflammation *vs.* Placebo	**1.92 (1.12, 3.28) ***	**2.33 (1.09, 5.27) ***
Fibrosis *vs.* Placebo	6.33 (0.67, 60.16)	9.04 (0.60, 376.10)
Modify gut microbiota *vs.* Placebo	**4.36 (1.54, 12.33) ***	**4.60 (1.42, 15.69) ***
Inflammation vs. Energy	0.80 (0.31, 2.02)	0.70 (0.27, 1.72)
Fibrosis *vs.* Energy	/	2.73 (0.15, 118.29)
Modify gut microbiota *vs.* Energy	/	1.39 (0.31, 5.80)
Fibrosis *vs.* Inflammation	/	3.91 (0.22, 168.79)
Modify gut microbiota *vs.* Inflammation	/	1.97 (0.48, 8.17)
Modify gut microbiota *vs.* Fibrosis	/	0.50 (0.01, 10.13)

Note: Results were presented as odd ratios (95% credible intervals) unless otherwise stated. Numbers in bold and marked with * represent statistically significant differences, *P* value < 0.05.

Table S2. Pooled summary estimates derived from direct and network meta-analysis informing on the comparative efficacy of pharmacological strategies for reducing liver enzymes in pediatric NAFLD patients.

**Comparisons**	**Meta-analysis**	
	**Direct**	**Network**
Secondary outcomes: ALT		
Energy *vs.* Placebo	**-9.495 (-13.823, -5.168) ***	**-12.346 (-20.098, -5.177)**
Inflammation *vs.* Placebo	**-10.553 (-18.116, -2.990)**	**-10.642 (-20.051, -3.537)**
Fibrosis *vs.* Placebo	**-3.602 (-7.193, -0.012) ***	-3.123 (-18.595, 12.952)
Modify gut microbiota *vs.* Placebo	-2.936 (-12.995, 7.124)	-2.994 (-14.183, 8.877)
Inflammation *vs.* Energy	-1.445 (-6.265, 3.375)	1.738 (-9.048, 10.615)
Fibrosis *vs.* Energy	/	9.226 (-7.687, 27.374)
Modify gut microbiota *vs.* Energy	/	9.363 (-3.750, 23.724)
Fibrosis *vs.* Inflammation	/	7.472 (-8.802, 26.936)
Modify gut microbiota *vs.* Inflammation	/	7.656 (-5.178, 23.358)
Modify gut microbiota *vs.* Fibrosis	/	0.099 (-19.361, 19.686)
Secondary outcomes: AST		
Energy *vs.* Placebo	**-3.150 (-5.627, -0.672) ***	-4.302 (-11.337, 1.863)
Inflammation *vs.* Placebo	**-5.725 (-10.796, -0.654) ***	**-6.092 (-14.167, -0.224)**
Fibrosis *vs.* Placebo	4.700 (-10.818, 20.218)	-10.957 (-20.732, 2.959)
Modify gut microbiota *vs.* Placebo	0.701 (-9.580, 10.982)	0.592 (-10.340, 11.859)
Inflammation *vs.* Energy	-1.149 (-4.652, 2.354)	-1.703 (-10.725, 5.687)
Fibrosis *vs.* Energy	/	-6.613 (-17.763, 9.470)
Modify gut microbiota *vs.* Energy	/	4.896 (-7.391, 18.354)
Fibrosis *vs.* Inflammation	/	-4.734 (-15.852, 12.313)
Modify gut microbiota *vs.* Inflammation	/	6.655 (-5.084, 21.077)
Modify gut microbiota *vs.* Fibrosis	/	11.550 (-6.567, 25.903)

Note: Results were presented as weighted mean difference (95% credible intervals) unless otherwise stated. Numbers in bold and marked with * represent statistically significant differences, *P* value < 0.05.

Table S3. Pooled summary estimates derived from direct and network meta-analysis informing on the comparative efficacy of pharmacological strategies for improving lipid profiles in pediatric NAFLD patients

Comparisons	Meta-analysis	
	Direct	Network
Secondary outcomes: TC		
Energy *vs.* Placebo	-0.250 (-0.723, 0.223)	-0.670 (-5.571, 3.824)
Inflammation *vs.* Placebo	-5.725 (-15.221, 3.771)	-0.176 (-5.584, 4.501)
Fibrosis *vs.* Placebo	-2.600 (-15.470, 10.270)	-2.512 (-17.728, 12.610)
Modify gut microbiota *vs.* Placebo	-8.322 (-22.691, 6.046)	-8.283 (-23.708, 7.319)
Inflammation *vs.* Energy	4.262 (-0.146, 8.669)	0.594 (-5.646, 6.259)
Fibrosis *vs.* Energy	/	-1.745 (-17.555, 14.100)
Modify gut microbiota *vs.* Energy	/	-7.514 (-23.607, 8.673)
Fibrosis *vs.* Inflammation	/	-2.294 (-18.073, 13.854)
Modify gut microbiota *vs.* Inflammation	/	-8.029 (-24.284, 8.354)
Modify gut microbiota *vs.* Fibrosis	/	-5.866 (-27.507, 16.038)
Secondary outcomes: TG		
Energy *vs.* Placebo	**-5.675 (-11.268, -0.082) ***	**-14.004 (-24.889, -4.149)**
Inflammation *vs.* Placebo	-5.136 (-19.479, 9.208)	-3.062 (-15.828, 8.786)
Fibrosis *vs.* Placebo	**-8.336 (-15.158, -1.513)**	-5.501 (-26.588, 17.106)
Modify gut microbiota *vs.* Placebo	3.664 (-9.308, 16.636)	3.891 (-14.140, 21.888)
Inflammation *vs.* Energy	**20.183 (1.946, 38.420) ***	10.992 (-3.208, 24.886)
Fibrosis *vs.* Energy	/	8.495 (-14.490, 34.132)
Modify gut microbiota *vs.* Energy	/	17.904 (-2.462, 39.345)
Fibrosis *vs.* Inflammation	/	-2.479 (-26.242, 24.151)
Modify gut microbiota *vs.* Inflammation	/	6.948 (-14.388, 29.446)
Modify gut microbiota *vs.* Fibrosis	/	9.336 (-19.572, 37.017)
Secondary outcomes: LDL-C		
Energy *vs.* Placebo	**-0.434 (-0.789, -0.080) ***	-1.381 (-6.750, 4.016)
Inflammation *vs.* Placebo	-4.153 (-10.673, 2.366)	-4.872 (-10.154, 0.089)
Fibrosis *vs.* Placebo	1.818 (-3.774, 7.410)	1.470 (-7.697, 10.445)
Modify gut microbiota *vs.* Placebo	-4.614 (-13.637, 4.410)	-4.443 (-15.955, 7.138)
Inflammation *vs.* Energy	0.274 (-3.355, 3.902)	-3.445 (-10.004, 2.685)
Fibrosis *vs.* Energy	/	2.892 (-7.831, 13.322)
Modify gut microbiota *vs.* Energy	/	-3.025 (-15.758, 9.690)
Fibrosis *vs.* Inflammation	/	6.355 (-4.007, 16.840)

(*Continued*)

Comparisons	Meta-analysis	
	Direct	Network
Modify gut microbiota *vs.* Inflammation	/	0.433 (-12.085, 13.314)
Modify gut microbiota *vs.* Fibrosis	/	-5.932 (-20.510, 8.816)
Secondary outcomes: HDL-C		
Energy *vs.* Placebo	**1.879 (0.057, 3.700) ***	**3.996 (1.374, 6.830) ***
Inflammation *vs.* Placebo	1.442 (-1.405, 4.289)	1.003 (-1.791, 4.175)
Fibrosis *vs.* Placebo	1.026 (-4.626, 6.677)	0.758 (-4.436, 6.466)
Modify gut microbiota *vs.* Placebo	-0.888 (-4.055, 2.279)	-0.961 (-6.272, 4.360)
Inflammation *vs.* Energy	**-2.255 (-4.271, -0.240) ***	-2.998 (-6.361, 0.575)
Fibrosis *vs.* Energy	/	-3.241 (-9.122, 3.004)
Modify gut microbiota *vs.* Energy	/	-4.971 (-11.013, 0.917)
Fibrosis *vs.* Inflammation	/	-0.251 (-6.298, 6.015)
Modify gut microbiota *vs.* Inflammation	/	-1.979 (-8.237, 3.984)
Modify gut microbiota *vs.* Fibrosis	/	-1.721 (-9.625, 5.647)

Note: Results were presented as weighted mean difference (95% credible intervals) unless otherwise stated. Numbers in bold and marked with * represent statistically significant differences, *P* value < 0.05.

Table S4. Pooled summary estimates derived from direct and network meta-analysis informing on the comparative efficacy of pharmacological strategies for improving metabolic indicators in pediatric NAFLD patients.

Comparisons	Meta-analysis	
	Direct	Network
Secondary outcomes: BMI		
Energy *vs.* Placebo	**-1.071 (-1.876, -0.266) ***	**-1.089 (-2.102, -0.268) ***
Inflammation *vs.* Placebo	-0.253 (-0.778, 0.273)	-0.471 (-1.465, 0.237)
Fibrosis *vs.* Placebo	0.000 (-0.599, 0.599)	-0.009 (-1.610, 1.618)
Modify gut microbiota *vs.* Placebo	**-2.300 (-2.899, -1.701) ***	**-2.294 (-3.916, -0.684) ***
Inflammation *vs.* Energy	**0.719 (0.265, 1.173) ***	0.629 (-0.442, 1.560)
Fibrosis *vs.* Energy	/	1.075 (-0.653, 3.054)
Modify gut microbiota *vs.* Energy	/	-1.212 (-2.963, 0.749)
Fibrosis *vs.* Inflammation	/	0.447 (-1.163, 2.465)
Modify gut microbiota *vs.* Inflammation	/	-1.842 (-3.470, 0.162)
Modify gut microbiota *vs.* Fibrosis	/	**-2.290 (-4.596, -0.034) ***
Secondary outcomes: HOMA-IR		
Energy *vs.* Placebo	-0.577 (-1.203, 0.049)	-0.995 (-1.927, 0.029)
Inflammation *vs.* Placebo	**-1.096 (-1.774, -0.418) ***	-0.646 (-1.740, 0.618)
Fibrosis *vs.* Placebo	**3.400 (0.130, 6.670) ***	3.395 (-0.563, 7.391)
Modify gut microbiota *vs.* Placebo	0.200 (-0.992, 1.392)	0.198 (-2.409, 2.816)
Inflammation *vs.* Energy	0.473 (-0.166, 1.112)	0.348 (-0.870, 1.647)
Fibrosis *vs.* Energy	/	**4.383 (0.295, 8.475) ***
Modify gut microbiota *vs.* Energy	/	1.191 (-1.641, 3.943)
Fibrosis *vs.* Inflammation	/	4.025 (-0.140, 8.153)
Modify gut microbiota *vs.* Inflammation	/	0.842 (-2.102, 3.634)
Modify gut microbiota *vs.* Fibrosis	/	-3.199 (-7.961, 1.533)

Note: Results were presented as weighted mean difference (95% credible intervals) unless otherwise stated. Numbers in bold and marked with * represent statistically significant differences, *P* value < 0.05.

Supplementary File 9. Distribution of potential effect modifiers across mechanistic pathway nodes

Table S1. Summary table of the distribution of potential effect modifiers across mechanistic pathway nodes.

Effect Modifier	**Energy** (9 studies, 604 patients)	**Inflammation** (9 studies, 728 patients)	**Fibrosis** (2 studies, 107 patients)	**Gut Microbiota** (3 studies, 184 patients)
**Baseline Severity**				
Steatosis grade (ultrasound)				
— Grade 1–3 (weighted mean, range)	1.4 (1.1–1.8)*	1.3 (1.1–1.5)*	1.6 (1 study)	1.3 (1.2–1.4)
— Ultrasound-confirmed only (no grade)	n = 152 (3 study)	n = 146 (2 study)	/	/
Steatosis score (Biopsy)				
— Grade 1–3 (weighted mean, range)	2.3 (2.1–2.6)	2.3 (2.2–2.5)	2.5 (1 study)	2.3 (1 study)
— NAS score (weighted mean, range)	4.5 (4.4–4.6)	4.6 (4.2–4.8)	4.6 (1 study)	4.5 (1 study)
**Diagnostic Modality**				
Ultrasound- based (%)	65.6% (396/604)	49.7% (362/728)	37.4% (40/107)	65.2% (120/184)
Biopsy- based (%)	34.4% (208/604)	50.3% (366/728)	62.6% (67/107)	34.8% (64/184)
**Participant Characteristics**				
Age, years (weighted mean, range)	12.2 (8.2–13.9)	11.8 (7.4–13.7)	12.6 (11.8–13.0)	11.6 (10.5–12.7)
Sex, proportion of female (%)	44.0% (266/604)	39.0% (284/728)	33.6% (36/107)	40.2% (74/184)
BMI, kg/m^2 [#] (weighted mean, range)	29.4 (26.6–34.9)[†]	30.4 (26.7–34.0)[†]	31.6 (25.8–35.0)	28.7 (27.1–29.4)[†]
BMI SDS [#] (weighted mean, range)	2.36 (2.15–2.56)[††]	3.10 (1 study)	/	2.56 (1 study)
**Co- interventions**				
Lifestyle modification (%)	100.0% (604/604)	100.0% (728/728)	100.0% (107/107)	100.0% (184/184)
**Treatment/evaluation period**				
Median (range), months	6 (3–24)	6 (3–24)	4 (3–6)	4 (3–6)
**Follow-up duration**				
Median (range), months	6 (3–30)	6 (3–30)	6 (3–12)	4 (3–6)

Note: NAS score — NAFLD activity score; BMI — Body mass index; BMI SDS — Body mass index standard deviation score;

* Weighted mean steatosis grade was calculated based on studies that explicitly reported ultrasound grading (scale 1–3). Studies that confirmed NAFLD by ultrasound without reporting the specific grade are listed separately under "ultrasound-confirmed only (no grade)" to reflect the available information without imputing missing data.

[#] BMI (kg/m^2) and BMI SDS are different indicators for assessing weight status and cannot be directly converted. In this table, they are presented separately to reflect the actual reporting practices of the individual studies.

[†] The weighted mean of BMI was calculated based on studies that reported raw BMI values.

[††] The weighted mean of BMI SDS was calculated based on studies that reported raw BMI SDS values.

Supplementary File 10. Node-splitting analysis of inconsistency

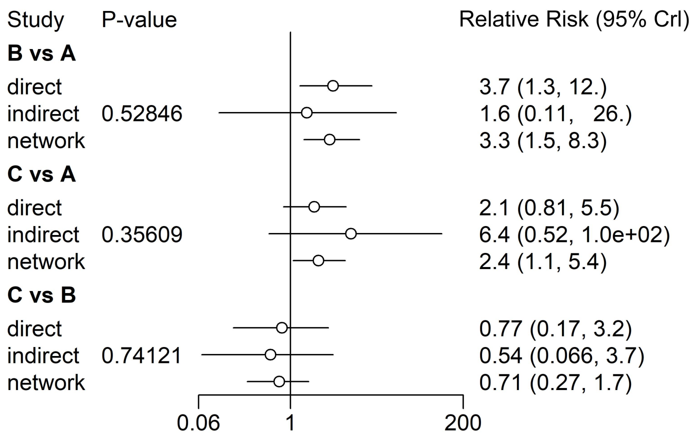

Figure S1. Node-splitting analysis of inconsistency for the primary outcome of this study

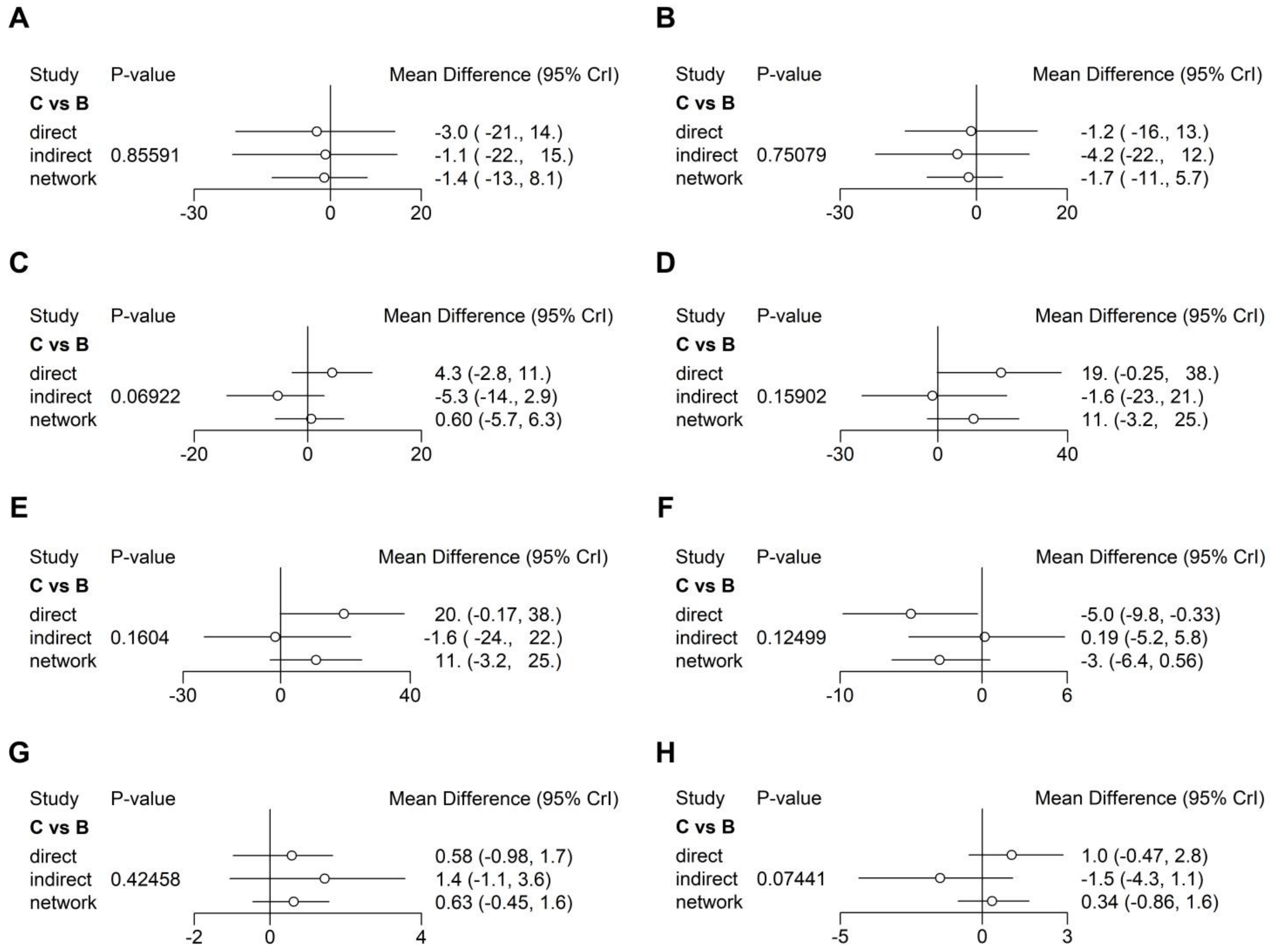

Figure S2. Node-splitting analysis of inconsistency for the secondary outcomes of this study.

(A) ALT, (B) AST, (C) TC, (D) TG, (E) LDL-C, (F) HDL-C, (G) BMI, (H) HOMA-IR.

Table S1. A summary table comparing the degree of fit of the consistency and non-consistency models

Outcome	Model	Dbar	pD	Data points	Ratio	I^2	DIC value
Hepatic steatosis	Consistency	31.652	26.008	30	1.055	8%	57.659
	Non-consistency	31.372	26.520	30	1.046	8%	57.892
ALT	Consistency	36.299	29.819	34	1.068	9%	66.119
	Non-consistency	36.368	29.800	34	1.070	9%	66.168
AST	Consistency	30.717	25.075	28	1.097	12%	55.792
	Non-consistency	30.627	25.061	28	1.094	12%	55.688
TC	Consistency	27.209	20.829	28	0.972	0.8%	48.039
	Non-consistency	27.280	20.818	28	0.974	1%	48.099
TG	Consistency	34.674	30.015	37	0.937	0%	64.689
	Non-consistency	34.685	30.043	37	0.937	0%	64.728
LDL-C	Consistency	27.130	22.605	28	0.969	0.5%	49.735
	Non-consistency	27.158	22.587	28	0.969	0.6%	49.744
HDL-C	Consistency	28.184	24.871	28	1.007	4%	53.055
	Non-consistency	28.174	24.894	28	1.006	4%	53.069
BMI	Consistency	21.063	17.676	23	0.916	0%	38.739
	Non-consistency	21.108	17.745	23	0.918	0%	38.853
HOMA-IR	Consistency	27.453	22.481	25	1.098	13%	49.934
	Non-consistency	27.291	22.364	25	1.092	12%	49.655

Table S2. A summary table comparing the degree of fit of the fixed effects and random effects models

Outcomes	Model	Dbar	pD	Data points	Ratio	I^2	DIC value
Hepatic steatosis	Fixed	47.941	18.239	30	1.598	40%	66.180
	Random	31.513	25.978	30	1.050	8%	57.491
ALT	Fixed	68.737	20.012	34	2.022	52%	88.749
	Random	36.535	29.783	34	1.075	10%	66.319
AST	Fixed	49.980	17.009	28	1.785	46%	66.990
	Random	30.694	25.026	28	1.096	12%	55.719
TC	Fixed	31.513	16.987	28	1.125	14%	48.500
	Random	27.213	20.845	28	0.972	0.8%	48.058
TG	Fixed	75.167	21.144	37	2.032	52%	96.311
	Random	34.681	30.046	37	0.937	0%	64.728
LDL-C	Fixed	38.348	16.997	28	1.370	30%	55.345
	Random	27.125	22.643	28	0.9687	0.5%	49.768
HDL-C	Fixed	118.352	17.034	28	4.227	77%	135.386
	Random	28.177	24.860	28	1.006	4%	53.037
BMI	Fixed	25.920	13.997	23	1.127	15%	39.917
	Random	21.108	17.747	23	0.918	0%	38.855
HOMA-IR	Fixed	46.367	15.011	25	1.855	48%	61.378
	Random	27.321	22.350	25	1.093	12%	49.671

Supplementary File 11. Sensitivity analysis after excluding trials with a high risk of bias.

Table S1. Sensitivity analysis: a summary table of the results of the primary analysis and of the results after excluding trials with a high risk of bias.

Comparisons	Sensitivity analysis	
	Main analysis	HQ trials analysis
Primary outcome: an improvement in hepatic steatosis		
Energy *vs.* Placebo	**3.32 (1.52, 8.26) ***	**3.99 (1.38, 14.01) ***
Inflammation *vs.* Placebo	**2.33 (1.09, 5.27) ***	**2.17 (1.05, 5.80) ***
Fibrosis *vs.* Placebo	9.04 (0.60, 376.10)	/
Modify gut microbiota *vs.* Placebo	**4.60 (1.42, 15.69) ***	**4.69 (1.21, 18.76) ***
Inflammation *vs.* Energy	0.70 (0.27, 1.72)	0.54 (0.14, 1.81)
Fibrosis *vs.* Energy	2.73 (0.15, 118.29)	/
Modify gut microbiota *vs.* Energy	1.39 (0.31, 5.80)	1.17 (0.18, 6.51)
Fibrosis *vs.* Inflammation	3.91 (0.22, 168.79)	/
Modify gut microbiota *vs.* Inflammation	1.97 (0.48, 8.17)	2.16 (0.4, 11.37)
Modify gut microbiota *vs.* Fibrosis	0.50 (0.01, 10.13)	/
Secondary outcomes: ALT		
Energy *vs.* Placebo	**-12.346 (-20.098, -5.177) ***	**-13.278 (-24.533, -2.898) ***
Inflammation *vs.* Placebo	**-10.642 (-20.051, -3.537) ***	**-13.951 (-26.735, -4.096) ***
Fibrosis *vs.* Placebo	-3.123 (-18.595, 12.952)	0.987 (-37.223, 39.008)
Modify gut microbiota *vs.* Placebo	-2.994 (-14.183, 8.877)	-2.741 (-16.624, 11.933)
Inflammation *vs.* Energy	1.738 (-9.048, 10.615)	-0.620 (-15.208, 11.650)
Fibrosis *vs.* Energy	9.226 (-7.687, 27.374)	14.351 (-25.074, 53.970)
Modify gut microbiota *vs.* Energy	9.363 (-3.750, 23.724)	10.516 (-6.551, 29.284)
Fibrosis *vs.* Inflammation	7.472 (-8.802, 26.936)	15.185 (-23.951, 55.495)
Modify gut microbiota *vs.* Inflammation	7.656 (-5.178, 23.358)	11.168 (-5.184, 31.340)
Modify gut microbiota *vs.* Fibrosis	0.099 (-19.361, 19.686)	-3.674 (-43.961, 37.336)
Secondary outcomes: AST		
Energy *vs.* Placebo	-4.302 (-11.337, 1.863)	-4.672 (-15.742, 4.895)
Inflammation *vs.* Placebo	**-6.092 (-14.167, -0.224) ***	**-8.314 (-19.145, -0.229) ***
Fibrosis *vs.* Placebo	-10.957 (-20.732, 2.959)	4.735 (-18.463, 27.749)
Modify gut microbiota *vs.* Placebo	0.592 (-10.340, 11.859)	0.527 (-12.476, 14.127)
Inflammation *vs.* Energy	-1.703 (-10.725, 5.687)	-3.493 (-15.634, 7.046)
Fibrosis *vs.* Energy	-6.613 (-17.763, 9.470)	9.504 (-15.302, 35.324)
Modify gut microbiota *vs.* Energy	4.896 (-7.391, 18.354)	5.238 (-10.592, 23.020)

Fibrosis *vs.* Inflammation	-4.734 (-15.852, 12.313)	13.098 (-10.882, 39.109)
Modify gut microbiota *vs.* Inflammation	6.655 (-5.084, 21.077)	8.815 (-5.706, 26.867)
Modify gut microbiota *vs.* Fibrosis	11.550 (-6.567, 25.903)	-4.174 (-30.417, 22.650)
Secondary outcomes: TC		
Energy *vs.* Placebo	-0.670 (-5.571, 3.824)	-0.925 (-9.004, 7.602)
Inflammation *vs.* Placebo	-0.176 (-5.584, 4.501)	0.680 (-7.389, 7.767)
Fibrosis *vs.* Placebo	-2.512 (-17.728, 12.610)	-2.500 (-20.402, 15.165)
Modify gut microbiota *vs.* Placebo	-8.283 (-23.708, 7.319)	-8.508 (-25.378, 8.444)
Inflammation *vs.* Energy	0.594 (-5.646, 6.259)	1.618 (-8.357, 10.082)
Fibrosis *vs.* Energy	-1.745 (-17.555, 14.100)	-1.623 (-21.530, 17.625)
Modify gut microbiota *vs.* Energy	-7.514 (-23.607, 8.673)	-7.603 (-26.278, 11.115)
Fibrosis *vs.* Inflammation	-2.294 (-18.073, 13.854)	-3.154 (-22.096, 16.407)
Modify gut microbiota *vs.* Inflammation	-8.029 (-24.284, 8.354)	-9.102 (-27.264, 9.602)
Modify gut microbiota *vs.* Fibrosis	-5.866 (-27.507, 16.038)	-5.986 (-30.394, 18.702)
Secondary outcomes: TG		
Energy *vs.* Placebo	**14.004 (-24.889, -4.149) ***	**-15.299 (-28.187, -2.966) ***
Inflammation *vs.* Placebo	-3.062 (-15.828, 8.786)	-3.163 (-17.891, 9.877)
Fibrosis *vs.* Placebo	-5.501 (-26.588, 17.106)	7.120 (-36.478, 50.810)
Modify gut microbiota *vs.* Placebo	3.891 (-14.140, 21.888)	3.752 (-14.333, 22.077)
Inflammation *vs.* Energy	10.992 (-3.208, 24.886)	12.148 (-4.140, 27.101)
Fibrosis *vs.* Energy	8.495 (-14.490, 34.132)	22.447 (-22.760, 68.172)
Modify gut microbiota *vs.* Energy	17.904 (-2.462, 39.345)	19.062 (-2.796, 41.589)
Fibrosis *vs.* Inflammation	-2.479 (-26.242, 24.151)	10.363 (-34.786, 56.611)
Modify gut microbiota *vs.* Inflammation	6.948 (-14.388, 29.446)	6.912 (-14.982, 30.864)
Modify gut microbiota *vs.* Fibrosis	9.336 (-19.572, 37.017)	-3.389 (-50.547, 44.098)
Secondary outcomes: LDL-C		
Energy *vs.* Placebo	-1.381 (-6.750, 4.016)	-0.932 (-6.995, 5.832)
Inflammation *vs.* Placebo	-4.872 (-10.154, 0.089)	-2.420 (-7.960, 2.680)
Fibrosis *vs.* Placebo	1.470 (-7.697, 10.445)	-0.550 (-14.183, 13.135)
Modify gut microbiota *vs.* Placebo	-4.443 (-15.955, 7.138)	-4.382 (-15.380, 6.645)
Inflammation *vs.* Energy	-3.445 (-10.004, 2.685)	-1.467 (-8.672, 4.555)
Fibrosis *vs.* Energy	2.892 (-7.831, 13.322)	0.398 (-14.906, 15.163)
Modify gut microbiota *vs.* Energy	-3.025 (-15.758, 9.690)	-3.494 (-16.365, 9.111)
Fibrosis *vs.* Inflammation	6.355 (-4.007, 16.840)	1.911 (-12.580, 16.730)
Modify gut microbiota *vs.* Inflammation	0.433 (-12.085, 13.314)	-1.932 (-13.998, 10.381)
Modify gut microbiota *vs.* Fibrosis	-5.932 (-20.510, 8.816)	-3.822 (-21.486, 13.834)

Secondary outcomes: HDL-C		
Energy *vs.* Placebo	**3.996 (1.374, 6.830) ***	**4.092 (1.931, 6.909) ***
Inflammation *vs.* Placebo	1.003 (-1.791, 4.175)	-0.263 (-2.092, 2.947)
Fibrosis *vs.* Placebo	0.758 (-4.436, 6.466)	-0.996 (-5.708, 3.737)
Modify gut microbiota *vs.* Placebo	-0.961 (-6.272, 4.360)	-0.902 (-5.110, 3.260)
Inflammation *vs.* Energy	-2.998 (-6.361, 0.575)	-4.360 (-6.740, 0.193)
Fibrosis *vs.* Energy	-3.241 (-9.122, 3.004)	-5.121 (-10.751, 0.086)
Modify gut microbiota *vs.* Energy	-4.971 (-11.013, 0.917)	-5.027 (-10.164, 1.394)
Fibrosis *vs.* Inflammation	-0.251 (-6.298, 6.015)	-0.769 (-6.764, 3.919)
Modify gut microbiota *vs.* Inflammation	-1.979 (-8.237, 3.984)	-0.710 (-6.161, 3.679)
Modify gut microbiota *vs.* Fibrosis	-1.721 (-9.625, 5.647)	0.109 (-6.235, 6.325)
Secondary outcomes: BMI		
Energy *vs.* Placebo	**-1.089 (-2.102, -0.268) ***	**-0.797 (-1.774, -0.062) ***
Inflammation *vs.* Placebo	-0.471 (-1.465, 0.237)	-0.308 (-1.259, 0.299)
Fibrosis *vs.* Placebo	-0.009 (-1.610, 1.618)	0.004 (-1.396, 1.405)
Modify gut microbiota *vs.* Placebo	**-2.294 (-3.916, -0.684) ***	**-2.300 (-3.702, -0.897) ***
Inflammation *vs.* Energy	0.629 (-0.442, 1.560)	0.497 (-0.591, 1.295)
Fibrosis *vs.* Energy	1.075 (-0.653, 3.054)	0.804 (-0.811, 2.531)
Modify gut microbiota *vs.* Energy	-1.212 (-2.963, 0.749)	-1.502 (-3.108, 0.241)
Fibrosis *vs.* Inflammation	0.447 (-1.163, 2.465)	0.305 (-1.073, 2.103)
Modify gut microbiota *vs.* Inflammation	-1.842 (-3.470, 0.162)	-2.002 (-3.383, 0.185)
Modify gut microbiota *vs.* Fibrosis	**-2.290 (-4.596, -0.034) ***	**-2.299 (-4.295, -0.309) ***
Secondary outcomes: HOMA-IR		
Energy *vs.* Placebo	-0.995 (-1.927, 0.029)	-1.444 (-2.359, 0.579)
Inflammation *vs.* Placebo	-0.646 (-1.740, 0.618)	-0.906 (-1.734, 0.145)
Fibrosis *vs.* Placebo	3.395 (-0.563, 7.391)	3.414 (-0.229, 7.025)
Modify gut microbiota *vs.* Placebo	0.198 (-2.409, 2.816)	0.203 (-1.861, 2.275)
Inflammation *vs.* Energy	0.348 (-0.870, 1.647)	0.543 (-0.360, 1.697)
Fibrosis *vs.* Energy	**4.383 (0.295, 8.475) ***	**4.875 (1.119, 8.578) ***
Modify gut microbiota *vs.* Energy	1.191 (-1.641, 3.943)	1.648 (-0.593, 3.925)
Fibrosis *vs.* Inflammation	4.025 (-0.140, 8.153)	4.302 (-0.479, 7.987)
Modify gut microbiota *vs.* Inflammation	0.842 (-2.102, 3.634)	1.102 (-1.272, 3.285)
Modify gut microbiota *vs.* Fibrosis	-3.199 (-7.961, 1.533)	-3.213 (-7.367, 0.962)

Note: HQ – High quality.

Supplementary File 12. Sensitivity analysis after excluding trials involving multi-target agents.

Table S1. Sensitivity analysis: a summary table of the results of the primary analysis and of the results after excluding trials involving multi-target agents.

Comparisons	Sensitivity analysis	
	**Main analysis**	**Exclusion of multi-target agents**
Primary outcome: an improvement in hepatic steatosis		
Energy *vs.* Placebo	**3.32 (1.52, 8.26) ***	**3.43 (1.46, 9.12) ***
Inflammation *vs.* Placebo	**2.33 (1.09, 5.27) ***	**2.52 (1.04, 6.59) ***
Fibrosis *vs.* Placebo	9.04 (0.60, 376.10)	/
Modify gut microbiota *vs.* Placebo	**4.60 (1.42, 15.69) ***	**4.56 (1.35, 16.37) ***
Inflammation *vs.* Energy	0.70 (0.27, 1.72)	0.73 (0.25, 1.94)
Fibrosis *vs.* Energy	2.73 (0.15, 118.29)	/
Modify gut microbiota *vs.* Energy	1.39 (0.31, 5.80)	1.33 (0.27, 6.16)
Fibrosis *vs.* Inflammation	3.91 (0.22, 168.79)	/
Modify gut microbiota *vs.* Inflammation	1.97 (0.48, 8.17)	1.82 (0.39, 8.66)
Modify gut microbiota *vs.* Fibrosis	0.50 (0.01, 10.13)	/
Secondary outcomes: ALT		
Energy *vs.* Placebo	**-12.346 (-20.098, -5.177) ***	**-12.364 (-20.303, -4.978) ***
Inflammation *vs.* Placebo	**-10.642 (-20.051, -3.537) ***	**-10.75 (-20.445, -3.438) ***
Fibrosis *vs.* Placebo	-3.123 (-18.595, 12.952)	0.768 (-35.301, 36.919)
Modify gut microbiota *vs.* Placebo	-2.994 (-14.183, 8.877)	-2.997 (-14.486, 9.339)
Inflammation *vs.* Energy	1.738 (-9.048, 10.615)	1.644 (-9.446, 10.806)
Fibrosis *vs.* Energy	9.226 (-7.687, 27.374)	13.2 (-23.522, 50.07)
Modify gut microbiota *vs.* Energy	9.363 (-3.750, 23.724)	9.395 (-4.117, 24.24)
Fibrosis *vs.* Inflammation	7.472 (-8.802, 26.936)	11.705 (-24.838, 49.307)
Modify gut microbiota *vs.* Inflammation	7.656 (-5.178, 23.358)	7.785 (-5.366, 24.084)
Modify gut microbiota *vs.* Fibrosis	0.099 (-19.361, 19.686)	-3.706 (-41.512, 34.362)
Secondary outcomes: AST		
Energy *vs.* Placebo	-4.302 (-11.337, 1.863)	-4.018 (-10.339, 1.044)
Inflammation *vs.* Placebo	**-6.092 (-14.167, -0.224) ***	**-5.442 (-13.065, -0.479) ***
Fibrosis *vs.* Placebo	-10.957 (-20.732, 2.959)	4.723 (-14.323, 23.978)
Modify gut microbiota *vs.* Placebo	0.592 (-10.340, 11.859)	0.442 (-9.057, 10.398)
Inflammation *vs.* Energy	-1.703 (-10.725, 5.687)	-1.305 (-9.527, 4.957)
Fibrosis *vs.* Energy	-6.613 (-17.763, 9.470)	8.879 (-10.773, 29.069)
Modify gut microbiota *vs.* Energy	4.896 (-7.391, 18.354)	4.481 (-6.044, 16.554)

Fibrosis *vs.* Inflammation	-4.734 (-15.852, 12.313)	10.346 (-9.036, 31.27)
Modify gut microbiota *vs.* Inflammation	6.655 (-5.084, 21.077)	5.859 (-4.159, 19.063)
Modify gut microbiota *vs.* Fibrosis	11.550 (-6.567, 25.903)	-4.257 (-25.529, 17.302)
Secondary outcomes: TC		
Energy *vs.* Placebo	-0.670 (-5.571, 3.824)	-0.683 (-5.693, 3.909)
Inflammation *vs.* Placebo	-0.176 (-5.584, 4.501)	-0.229 (-5.644, 4.547)
Fibrosis *vs.* Placebo	-2.512 (-17.728, 12.610)	-2.639 (-17.89, 12.775)
Modify gut microbiota *vs.* Placebo	-8.283 (-23.708, 7.319)	-8.201 (-23.801, 7.211)
Inflammation *vs.* Energy	0.594 (-5.646, 6.259)	0.573 (-5.750, 6.360)
Fibrosis *vs.* Energy	-1.745 (-17.555, 14.100)	-1.817 (-17.707, 14.242)
Modify gut microbiota *vs.* Energy	-7.514 (-23.607, 8.673)	-7.406 (-23.68, 8.693)
Fibrosis *vs.* Inflammation	-2.294 (-18.073, 13.854)	-2.326 (-18.237, 13.979)
Modify gut microbiota *vs.* Inflammation	-8.029 (-24.284, 8.354)	-7.880 (-24.151, 8.420)
Modify gut microbiota *vs.* Fibrosis	-5.866 (-27.507, 16.038)	-5.585 (-27.468, 16.298)
Secondary outcomes: TG		
Energy *vs.* Placebo	**14.004 (-24.889, -4.149) ***	**-14.071 (-25.195, -3.95) ***
Inflammation *vs.* Placebo	-3.062 (-15.828, 8.786)	-3.082 (-16.279, 8.966)
Fibrosis *vs.* Placebo	-5.501 (-26.588, 17.106)	6.862 (-37.172, 50.61)
Modify gut microbiota *vs.* Placebo	3.891 (-14.140, 21.888)	3.895 (-14.495, 22.176)
Inflammation *vs.* Energy	10.992 (-3.208, 24.886)	11.011 (-3.468, 25.197)
Fibrosis *vs.* Energy	8.495 (-14.490, 34.132)	20.950 (-24.022, 66.252)
Modify gut microbiota *vs.* Energy	17.904 (-2.462, 39.345)	17.995 (-2.908, 39.709)
Fibrosis *vs.* Inflammation	-2.479 (-26.242, 24.151)	9.983 (-35.475, 55.932)
Modify gut microbiota *vs.* Inflammation	6.948 (-14.388, 29.446)	6.957 (-14.805, 29.882)
Modify gut microbiota *vs.* Fibrosis	9.336 (-19.572, 37.017)	-2.927 (-50.19, 44.764)
Secondary outcomes: LDL-C		
Energy *vs.* Placebo	-1.381 (-6.750, 4.016)	-1.389 (-7.075, 4.386)
Inflammation *vs.* Placebo	-4.872 (-10.154, 0.089)	-4.905 (-10.509, 0.359)
Fibrosis *vs.* Placebo	1.470 (-7.697, 10.445)	-0.563 (-15.873, 14.686)
Modify gut microbiota *vs.* Placebo	-4.443 (-15.955, 7.138)	-4.461 (-16.428, 7.713)
Inflammation *vs.* Energy	-3.445 (-10.004, 2.685)	-3.479 (-10.514, 3.052)
Fibrosis *vs.* Energy	2.892 (-7.831, 13.322)	0.857 (-15.482, 17.051)
Modify gut microbiota *vs.* Energy	-3.025 (-15.758, 9.690)	-3.024 (-16.263, 10.371)
Fibrosis *vs.* Inflammation	6.355 (-4.007, 16.840)	4.35 (-11.725, 20.647)
Modify gut microbiota *vs.* Inflammation	0.433 (-12.085, 13.314)	0.469 (-12.57, 13.9)
Modify gut microbiota *vs.* Fibrosis	-5.932 (-20.510, 8.816)	-3.917 (-23.145, 15.706)

Secondary outcomes: HDL-C		
Energy *vs.* Placebo	**3.996 (1.374, 6.830) ***	**3.994 (1.400, 6.804) ***
Inflammation *vs.* Placebo	1.003 (-1.791, 4.175)	1.005 (-1.768, 4.193)
Fibrosis *vs.* Placebo	0.758 (-4.436, 6.466)	-0.977 (-7.619, 5.682)
Modify gut microbiota *vs.* Placebo	-0.961 (-6.272, 4.360)	-0.966 (-6.306, 4.386)
Inflammation *vs.* Energy	-2.998 (-6.361, 0.575)	-2.986 (-6.353, 0.573)
Fibrosis *vs.* Energy	-3.241 (-9.122, 3.004)	-4.983 (-12.247, 2.103)
Modify gut microbiota *vs.* Energy	-4.971 (-11.013, 0.917)	-4.972 (-11.011, 0.952)
Fibrosis *vs.* Inflammation	-0.251 (-6.298, 6.015)	-1.987 (-9.474, 5.104)
Modify gut microbiota *vs.* Inflammation	-1.979 (-8.237, 3.984)	-1.979 (-8.251, 4.014)
Modify gut microbiota *vs.* Fibrosis	-1.721 (-9.625, 5.647)	0.012 (-8.542, 8.547)
Secondary outcomes: BMI		
Energy *vs.* Placebo	**-1.089 (-2.102, -0.268) ***	**-1.081 (-2.117, -0.255) ***
Inflammation *vs.* Placebo	-0.471 (-1.465, 0.237)	-0.465 (-1.481, 0.239)
Fibrosis *vs.* Placebo	-0.009 (-1.610, 1.618)	0.004 (-1.641, 1.638)
Modify gut microbiota *vs.* Placebo	**-2.294 (-3.916, -0.684) ***	**-2.302 (-3.923, -0.675) ***
Inflammation *vs.* Energy	0.629 (-0.442, 1.560)	0.624 (-0.461, 1.571)
Fibrosis *vs.* Energy	1.075 (-0.653, 3.054)	1.075 (-0.686, 3.065)
Modify gut microbiota *vs.* Energy	-1.212 (-2.963, 0.749)	-1.226 (-2.978, 0.772)
Fibrosis *vs.* Inflammation	0.447 (-1.163, 2.465)	0.451 (-1.195, 2.481)
Modify gut microbiota *vs.* Inflammation	-1.842 (-3.470, 0.162)	-1.85 (-3.491, 0.179)
Modify gut microbiota *vs.* Fibrosis	**-2.290 (-4.596, -0.034) ***	-2.301 (-4.607, 0.003)
Secondary outcomes: HOMA-IR		
Energy *vs.* Placebo	-0.995 (-1.927, 0.029)	-0.993 (-1.918, 0.022)
Inflammation *vs.* Placebo	-0.646 (-1.740, 0.618)	-0.653 (-1.735, 0.614)
Fibrosis *vs.* Placebo	3.395 (-0.563, 7.391)	3.424 (-0.548, 7.333)
Modify gut microbiota *vs.* Placebo	0.198 (-2.409, 2.816)	0.194 (-2.410, 2.790)
Inflammation *vs.* Energy	0.348 (-0.870, 1.647)	0.340 (-0.869, 1.643)
Fibrosis *vs.* Energy	**4.383 (0.295, 8.475) ***	**4.410 (0.298, 8.416) ***
Modify gut microbiota *vs.* Energy	1.191 (-1.641, 3.943)	1.186 (-1.630, 3.906)
Fibrosis *vs.* Inflammation	4.025 (-0.140, 8.153)	4.058 (-0.127, 8.088)
Modify gut microbiota *vs.* Inflammation	0.842 (-2.102, 3.634)	0.840 (-2.102, 3.613)
Modify gut microbiota *vs.* Fibrosis	-3.199 (-7.961, 1.533)	-3.224 (-7.894, 1.507)

Supplementary File 13. Sensitivity analysis after reclassification of multi-target agents.

Table S1. Sensitivity analysis: a summary table of the results of the primary analysis and of the results after reclassification of multi-target agents.

Comparisons	Sensitivity analysis	
	Main analysis	Reclassification of multi-target agents
Primary outcome: an improvement in hepatic steatosis		
Energy *vs.* Placebo	**3.32 (1.52, 8.26) ***	**3.18 (1.50, 7.45) ***
Inflammation *vs.* Placebo	**2.33 (1.09, 5.27) ***	**2.63 (1.22, 6.15) ***
Fibrosis *vs.* Placebo	9.04 (0.60, 376.10)	/
Modify gut microbiota *vs.* Placebo	**4.60 (1.42, 15.69) ***	**4.57 (1.43, 15.60) ***
Inflammation *vs.* Energy	0.70 (0.27, 1.72)	0.83 (0.33, 2.06)
Fibrosis *vs.* Energy	2.73 (0.15, 118.29)	/
Modify gut microbiota *vs.* Energy	1.39 (0.31, 5.80)	1.44 (0.34, 5.96)
Fibrosis *vs.* Inflammation	3.91 (0.22, 168.79)	/
Modify gut microbiota *vs.* Inflammation	1.97 (0.48, 8.17)	1.75 (0.41, 7.27)
Modify gut microbiota *vs.* Fibrosis	0.50 (0.01, 10.13)	/
Secondary outcomes: ALT		
Energy *vs.* Placebo	**-12.346 (-20.098, -5.177) ***	**-12.034 (-19.466, -5.12) ***
Inflammation *vs.* Placebo	**-10.642 (-20.051, -3.537) ***	**-9.238 (-17.609, -3.055) ***
Fibrosis *vs.* Placebo	-3.123 (-18.595, 12.952)	0.965 (-34.407, 36.472)
Modify gut microbiota *vs.* Placebo	-2.994 (-14.183, 8.877)	-3.048 (-13.786, 8.496)
Inflammation *vs.* Energy	1.738 (-9.048, 10.615)	2.819 (-7.201, 11.050)
Fibrosis *vs.* Energy	9.226 (-7.687, 27.374)	13.082 (-22.950, 49.363)
Modify gut microbiota *vs.* Energy	9.363 (-3.750, 23.724)	8.979 (-3.641, 22.864)
Fibrosis *vs.* Inflammation	7.472 (-8.802, 26.936)	10.410 (-25.401, 46.970)
Modify gut microbiota *vs.* Inflammation	7.656 (-5.178, 23.358)	6.224 (-5.782, 21.126)
Modify gut microbiota *vs.* Fibrosis	0.099 (-19.361, 19.686)	-4.007 (-41.041, 33.297)
Secondary outcomes: AST		
Energy *vs.* Placebo	-4.302 (-11.337, 1.863)	-4.944 (-11.774, 1.579)
Inflammation *vs.* Placebo	**-6.092 (-14.167, -0.224) ***	**-8.442 (-15.104, -2.511) ***
Fibrosis *vs.* Placebo	-10.957 (-20.732, 2.959)	4.636 (-16.409, 25.671)
Modify gut microbiota *vs.* Placebo	0.592 (-10.340, 11.859)	0.673 (-10.55, 12.151)
Inflammation *vs.* Energy	-1.703 (-10.725, 5.687)	-3.478 (-11.826, 4.329)
Fibrosis *vs.* Energy	-6.613 (-17.763, 9.470)	9.622 (-12.434, 31.746)
Modify gut microbiota *vs.* Energy	4.896 (-7.391, 18.354)	5.632 (-7.313, 19.038)

Fibrosis *vs.* Inflammation	-4.734 (-15.852, 12.313)	13.102 (-8.657, 35.241)
Modify gut microbiota *vs.* Inflammation	6.655 (-5.084, 21.077)	9.090 (-3.433, 22.631)
Modify gut microbiota *vs.* Fibrosis	11.550 (-6.567, 25.903)	-3.965 (-27.765, 20.065)
Secondary outcomes: TC		
Energy *vs.* Placebo	-0.670 (-5.571, 3.824)	-0.642 (-5.618, 3.943)
Inflammation *vs.* Placebo	-0.176 (-5.584, 4.501)	-0.180 (-5.572, 4.474)
Fibrosis *vs.* Placebo	-2.512 (-17.728, 12.610)	-2.522 (-17.692, 12.319)
Modify gut microbiota *vs.* Placebo	-8.283 (-23.708, 7.319)	-8.530 (-23.825, 6.914)
Inflammation *vs.* Energy	0.594 (-5.646, 6.259)	0.581 (-5.760, 6.259)
Fibrosis *vs.* Energy	-1.745 (-17.555, 14.100)	-1.755 (-17.584, 13.881)
Modify gut microbiota *vs.* Energy	-7.514 (-23.607, 8.673)	-7.781 (-23.676, 8.379)
Fibrosis *vs.* Inflammation	-2.294 (-18.073, 13.854)	-2.285 (-18.079, 13.515)
Modify gut microbiota *vs.* Inflammation	-8.029 (-24.284, 8.354)	-8.274 (-24.307, 8.118)
Modify gut microbiota *vs.* Fibrosis	-5.866 (-27.507, 16.038)	-5.993 (-27.349, 15.613)
Secondary outcomes: TG		
Energy *vs.* Placebo	**14.004 (-24.889, -4.149) ***	**-14.254 (-24.828, -4.518) ***
Inflammation *vs.* Placebo	-3.062 (-15.828, 8.786)	-4.300 (-15.453, 6.288)
Fibrosis *vs.* Placebo	-5.501 (-26.588, 17.106)	7.021 (-35.745, 50.390)
Modify gut microbiota *vs.* Placebo	3.891 (-14.140, 21.888)	3.827 (-13.987, 21.532)
Inflammation *vs.* Energy	10.992 (-3.208, 24.886)	9.951 (-3.090, 23.264)
Fibrosis *vs.* Energy	8.495 (-14.490, 34.132)	21.368 (-22.588, 66.075)
Modify gut microbiota *vs.* Energy	17.904 (-2.462, 39.345)	18.098 (-1.989, 38.957)
Fibrosis *vs.* Inflammation	-2.479 (-26.242, 24.151)	11.424 (-32.854, 56.276)
Modify gut microbiota *vs.* Inflammation	6.948 (-14.388, 29.446)	8.120 (-12.372, 29.216)
Modify gut microbiota *vs.* Fibrosis	9.336 (-19.572, 37.017)	-3.201 (-50.104, 43.232)
Secondary outcomes: LDL-C		
Energy *vs.* Placebo	-1.381 (-6.750, 4.016)	-1.092 (-6.785, 4.761)
Inflammation *vs.* Placebo	-4.872 (-10.154, 0.089)	-3.720 (-8.853, 1.181)
Fibrosis *vs.* Placebo	1.470 (-7.697, 10.445)	-0.523 (-15.892, 14.897)
Modify gut microbiota *vs.* Placebo	-4.443 (-15.955, 7.138)	-4.411 (-16.559, 7.780)
Inflammation *vs.* Energy	-3.445 (-10.004, 2.685)	-2.61 (-9.544, 3.880)
Fibrosis *vs.* Energy	2.892 (-7.831, 13.322)	0.604 (-15.981, 17.051)
Modify gut microbiota *vs.* Energy	-3.025 (-15.758, 9.690)	-3.286 (-16.757, 10.115)
Fibrosis *vs.* Inflammation	6.355 (-4.007, 16.840)	3.220 (-12.931, 19.573)
Modify gut microbiota *vs.* Inflammation	0.433 (-12.085, 13.314)	-0.662 (-13.689, 12.586)
Modify gut microbiota *vs.* Fibrosis	-5.932 (-20.510, 8.816)	-3.907 (-23.503, 15.851)

Secondary outcomes: HDL-C		
Energy *vs.* Placebo	**3.996 (1.374, 6.830) ***	**4.074 (1.506, 6.885) ***
Inflammation *vs.* Placebo	1.003 (-1.791, 4.175)	1.317 (-1.294, 4.396)
Fibrosis *vs.* Placebo	0.758 (-4.436, 6.466)	-1.008 (-7.682, 5.619)
Modify gut microbiota *vs.* Placebo	-0.961 (-6.272, 4.360)	-0.948 (-6.299, 4.386)
Inflammation *vs.* Energy	-2.998 (-6.361, 0.575)	-2.764 (-6.011, 0.742)
Fibrosis *vs.* Energy	-3.241 (-9.122, 3.004)	-5.069 (-12.382, 1.936)
Modify gut microbiota *vs.* Energy	-4.971 (-11.013, 0.917)	-5.027 (-11.143, 0.839)
Fibrosis *vs.* Inflammation	-0.251 (-6.298, 6.015)	-2.316 (-9.792, 4.631)
Modify gut microbiota *vs.* Inflammation	-1.979 (-8.237, 3.984)	-2.263 (-8.565, 3.569)
Modify gut microbiota *vs.* Fibrosis	-1.721 (-9.625, 5.647)	0.068 (-8.444, 8.570)
Secondary outcomes: BMI		
Energy *vs.* Placebo	**-1.089 (-2.102, -0.268) ***	**-1.083 (-2.105, -0.261) ***
Inflammation *vs.* Placebo	-0.471 (-1.465, 0.237)	-0.470 (-1.469, 0.231)
Fibrosis *vs.* Placebo	-0.009 (-1.610, 1.618)	0.002 (-1.61, 1.621)
Modify gut microbiota *vs.* Placebo	**-2.294 (-3.916, -0.684) ***	**-2.301 (-3.918, -0.689) ***
Inflammation *vs.* Energy	0.629 (-0.442, 1.560)	0.622 (-0.462, 1.556)
Fibrosis *vs.* Energy	1.075 (-0.653, 3.054)	1.076 (-0.652, 3.043)
Modify gut microbiota *vs.* Energy	-1.212 (-2.963, 0.749)	-1.229 (-2.969, 0.736)
Fibrosis *vs.* Inflammation	0.447 (-1.163, 2.465)	0.455 (-1.164, 2.465)
Modify gut microbiota *vs.* Inflammation	-1.842 (-3.470, 0.162)	-1.847 (-3.489, 0.171)
Modify gut microbiota *vs.* Fibrosis	**-2.290 (-4.596, -0.034) ***	**-2.304 (-4.586, -0.042) ***
Secondary outcomes: HOMA-IR		
Energy *vs.* Placebo	-0.995 (-1.927, 0.029)	-0.993 (-1.92, 0.034)
Inflammation *vs.* Placebo	-0.646 (-1.740, 0.618)	-0.651 (-1.733, 0.616)
Fibrosis *vs.* Placebo	3.395 (-0.563, 7.391)	3.41 (-0.572, 7.450)
Modify gut microbiota *vs.* Placebo	0.198 (-2.409, 2.816)	0.212 (-2.407, 2.809)
Inflammation *vs.* Energy	0.348 (-0.870, 1.647)	0.343 (-0.871, 1.651)
Fibrosis *vs.* Energy	**4.383 (0.295, 8.475) ***	**4.393 (0.273, 8.519) ***
Modify gut microbiota *vs.* Energy	1.191 (-1.641, 3.943)	1.200 (-1.638, 3.934)
Fibrosis *vs.* Inflammation	4.025 (-0.140, 8.153)	4.039 (-0.160, 8.213)
Modify gut microbiota *vs.* Inflammation	0.842 (-2.102, 3.634)	0.858 (-2.095, 3.611)
Modify gut microbiota *vs.* Fibrosis	-3.199 (-7.961, 1.533)	-3.198 (-7.955, 1.544)

Supplementary File 14. Sensitivity analysis of NAFLD diagnostic methods.

Table S1. A summary table for sensitivity analysis including the results of the primary analysis and those of ultrasound-based diagnosis or biopsy-based diagnosis.

Comparisons	Sensitivity analysis		
	Main analysis	Ultrasound	Biopsy
Primary outcome: an improvement in hepatic steatosis			
Energy *vs.* Placebo	**3.32 (1.52, 8.26) ***	**2.61 (1.04, 7.09) ***	6.30 (0.47, 123.71)
Inflammation *vs.* Placebo	**2.33 (1.09, 5.27) ***	1.71 (0.56, 5.12)	**3.55 (1.41, 16.66) ***
Fibrosis *vs.* Placebo	9.04 (0.60, 376.10)	9.03 (0.63, 318.76)	/
Modify gut microbiota *vs.* Placebo	**4.60 (1.42, 15.69) ***	**3.66 (1.08, 14.59) ***	**7.93 (1.14, 49.95) ***
Inflammation *vs.* Energy	0.70 (0.27, 1.72)	0.66 (0.20, 2.06)	0.56 (0.02, 11.18)
Fibrosis *vs.* Energy	2.73 (0.15, 118.29)	3.42 (0.21, 130.72)	/
Modify gut microbiota *vs.* Energy	1.39 (0.31, 5.80)	1.41 (0.26, 7.71)	1.25 (0.01, 144.01)
Fibrosis *vs.* Inflammation	3.91 (0.22, 168.79)	5.26 (0.31, 214.87)	/
Modify gut microbiota *vs.* Inflammation	1.97 (0.48, 8.17)	2.12 (0.39, 12.85)	2.24 (0.02, 216.9)
Modify gut microbiota *vs.* Fibrosis	0.50 (0.01, 10.13)	0.40 (0.01, 8.07)	/
Secondary outcomes: ALT			
Energy *vs.* Placebo	**-12.346 (-20.098, -5.177) ***	**-8.048 (-16.090, -1.279) ***	**-17.551 (-35.962, -0.147) ***
Inflammation *vs.* Placebo	**-10.642 (-20.051, -3.537) ***	**-5.572 (-11.052, -1.042) ***	**-23.086 (-45.475, -6.866) ***
Fibrosis *vs.* Placebo	-3.123 (-18.595, 12.952)	-3.890 (-16.321, 8.585)	0.826 (-41.603, 43.703)
Modify gut microbiota *vs.* Placebo	-2.994 (-14.183, 8.877)	-1.024 (-10.891, 10.687)	-9.068 (-40.82, 23.237)
Inflammation *vs.* Energy	1.738 (-9.048, 10.615)	4.445 (-4.274, 14.111)	-5.354 (-31.849, 14.898)
Fibrosis *vs.* Energy	9.226 (-7.687, 27.374)	4.091 (-9.527, 19.326)	18.459 (-27.26, 64.959)
Modify gut microbiota *vs.* Energy	9.363 (-3.750, 23.724)	7.030 (-4.591, 21.736)	8.5 (-27.563, 45.617)
Fibrosis *vs.* Inflammation	7.472 (-8.802, 26.936)	-0.316 (-14.468, 14.349)	24.333 (-20.493, 73.071)
Modify gut microbiota *vs.* Inflammation	7.656 (-5.178, 23.358)	2.559 (-9.250, 16.693)	13.853 (-19.701, 54.763)
Modify gut microbiota *vs.* Fibrosis	0.099 (-19.361, 19.686)	2.77 (-12.452, 20.373)	-9.938 (-62.790, 42.959)
Secondary outcomes: AST			
Energy *vs.* Placebo	-4.302 (-11.337, 1.863)	-3.401 (-9.111, 0.947)	-6.267 (-37.362, 23.91)
Inflammation *vs.* Placebo	**-6.092 (-14.167, -0.224) ***	-1.830 (-8.795, 4.088)	-12.550 (-33.384, 6.253)
Fibrosis *vs.* Placebo	-10.957 (-20.732, 2.959)	**-15.602 (-24.827, -6.26) ***	4.629 (-30.864, 40.401)
Modify gut microbiota *vs.* Placebo	0.592 (-10.340, 11.859)	0.266 (-7.409, 8.546)	/
Inflammation *vs.* Energy	-1.703 (-10.725, 5.687)	1.544 (-5.518, 9.055)	-6.152 (-37.613, 23.695)
Fibrosis *vs.* Energy	-6.613 (-17.763, 9.470)	**-12.308 (-22.007, -0.813) ***	10.937 (-35.348, 58.234)
Modify gut microbiota *vs.* Energy	4.896 (-7.391, 18.354)	3.721 (-4.762, 14.033)	/

Fibrosis *vs.* Inflammation	-4.734 (-15.852, 12.313)	**-13.826 (-24.456, -1.847) ***	17.276 (-22.400, 59.253)
Modify gut microbiota *vs.* Inflammation	6.655 (-5.084, 21.077)	2.113 (-7.339, 13.145)	/
Modify gut microbiota *vs.* Fibrosis	11.550 (-6.567, 25.903)	15.825 (3.978, 28.358)	/
Secondary outcomes: TC			
Energy *vs.* Placebo	-0.670 (-5.571, 3.824)	-0.562 (-6.718, 4.492)	-0.029 (-26.974, 27.283)
Inflammation *vs.* Placebo	-0.176 (-5.584, 4.501)	-0.970 (-7.878, 6.703)	-2.461 (-31.132, 17.363)
Fibrosis *vs.* Placebo	-2.512 (-17.728, 12.610)	/	-2.607 (-42.444, 37.085)
Modify gut microbiota *vs.* Placebo	-8.283 (-23.708, 7.319)	-8.168 (-23.679, 6.615)	/
Inflammation *vs.* Energy	0.594 (-5.646, 6.259)	-0.243 (-7.945, 8.980)	-2.404 (-38.041, 24.178)
Fibrosis *vs.* Energy	-1.745 (-17.555, 14.100)	/	-2.616 (-50.870, 45.268)
Modify gut microbiota *vs.* Energy	-7.514 (-23.607, 8.673)	-7.353 (-23.605, 8.397)	/
Fibrosis *vs.* Inflammation	-2.294 (-18.073, 13.854)	/	0.036 (-42.394, 50.492)
Modify gut microbiota *vs.* Inflammation	-8.029 (-24.284, 8.354)	-7.328 (-24.546, 8.986)	/
Modify gut microbiota *vs.* Fibrosis	-5.866 (-27.507, 16.038)	-0.562 (-6.718, 4.492)	/
Secondary outcomes: TG			
Energy *vs.* Placebo	**14.004 (-24.889, -4.149) ***	**-11.531 (-25.953, -0.678) ***	-18.150 (-46.687, 10.114)
Inflammation *vs.* Placebo	-3.062 (-15.828, 8.786)	-3.063 (-21.035, 14.332)	-4.894 (-34.409, 22.085)
Fibrosis *vs.* Placebo	-5.501 (-26.588, 17.106)	-8.900 (-35.218, 17.467)	6.912 (-51.603, 65.121)
Modify gut microbiota *vs.* Placebo	3.891 (-14.140, 21.888)	2.576 (-19.145, 24.379)	6.938 (-44.873, 58.990)
Inflammation *vs.* Energy	10.992 (-3.208, 24.886)	8.514 (-10.167, 28.336)	13.228 (-23.309, 47.859)
Fibrosis *vs.* Energy	8.495 (-14.490, 34.132)	2.496 (-25.600, 33.305)	25.011 (-39.714, 90.067)
Modify gut microbiota *vs.* Energy	17.904 (-2.462, 39.345)	14.074 (-10.435, 40.654)	25.071 (-33.802, 84.392)
Fibrosis *vs.* Inflammation	-2.479 (-26.242, 24.151)	-5.853 (-36.918, 26.009)	11.941 (-51.968, 77.748)
Modify gut microbiota *vs.* Inflammation	6.948 (-14.388, 29.446)	5.595 (-21.954, 33.693)	11.922 (-45.946, 72.044)
Modify gut microbiota *vs.* Fibrosis	9.336 (-19.572, 37.017)	11.429 (-22.676, 45.539)	-0.055 (-78.056, 78.146)
Secondary outcomes: LDL-C			
Energy *vs.* Placebo	-1.381 (-6.750, 4.016)	-0.914 (-8.688, 7.178)	-1.768 (-14.831, 10.287)
Inflammation *vs.* Placebo	-4.872 (-10.154, 0.089)	-7.25 (-15.251, 2.330)	-3.016 (-12.881, 5.467)
Fibrosis *vs.* Placebo	1.470 (-7.697, 10.445)	2.592 (-12.127, 17.188)	-0.494 (-17.105, 16.283)
Modify gut microbiota *vs.* Placebo	-4.443 (-15.955, 7.138)	-4.464 (-17.455, 8.894)	/
Inflammation *vs.* Energy	-3.445 (-10.004, 2.685)	-6.247 (-16.296, 4.872)	-1.178 (-14.080, 10.999)
Fibrosis *vs.* Energy	2.892 (-7.831, 13.322)	3.629 (-13.400, 19.923)	1.347 (-19.037, 22.556)
Modify gut microbiota *vs.* Energy	-3.025 (-15.758, 9.690)	-3.456 (-18.797, 11.926)	/
Fibrosis *vs.* Inflammation	6.355 (-4.007, 16.840)	9.871 (-8.128, 26.054)	2.635 (-15.834, 22.205)
Modify gut microbiota *vs.* Inflammation	0.433 (-12.085, 13.314)	2.744 (-13.536, 18.009)	/
Modify gut microbiota *vs.* Fibrosis	-5.932 (-20.510, 8.816)	-7.003 (-26.517, 12.748)	/

Secondary outcomes: HDL-C			
Energy *vs.* Placebo	**3.996 (1.374, 6.830) ***	**3.826 (0.017, 7.780) ***	4.281 (-2.166, 11.436)
Inflammation *vs.* Placebo	1.003 (-1.791, 4.175)	0.881 (-4.568, 6.109)	1.022 (-3.678, 7.825)
Fibrosis *vs.* Placebo	0.758 (-4.436, 6.466)	5.119 (-5.197, 15.435)	-0.989 (-10.926, 8.965)
Modify gut microbiota *vs.* Placebo	-0.961 (-6.272, 4.360)	-0.955 (-7.028, 5.093)	/
Inflammation *vs.* Energy	-2.998 (-6.361, 0.575)	-2.939 (-8.918, 2.708)	-3.404 (-10.193, 5.183)
Fibrosis *vs.* Energy	-3.241 (-9.122, 3.004)	1.295 (-9.814, 12.250)	-5.282 (-17.642, 6.501)
Modify gut microbiota *vs.* Energy	-4.971 (-11.013, 0.917)	-4.787 (-12.054, 2.351)	/
Fibrosis *vs.* Inflammation	-0.251 (-6.298, 6.015)	4.251 (-7.309, 15.948)	-1.947 (-14.542, 8.467)
Modify gut microbiota *vs.* Inflammation	-1.979 (-8.237, 3.984)	-1.833 (-9.838, 6.351)	/
Modify gut microbiota *vs.* Fibrosis	-1.721 (-9.625, 5.647)	-6.085 (-18.035, 5.873)	/
Secondary outcomes: BMI			
Energy *vs.* Placebo	**-1.089 (-2.102, -0.268) ***	**-1.557 (-3.040, 0-.227) ***	-0.854 (-2.706, 0.635)
Inflammation *vs.* Placebo	-0.471 (-1.465, 0.237)	-1.144 (-2.781, 0.647)	-0.253 (-1.848, 0.835)
Fibrosis *vs.* Placebo	-0.009 (-1.610, 1.618)	/	0.002 (-2.219, 2.214)
Modify gut microbiota *vs.* Placebo	**-2.294 (-3.916, -0.684) ***	/	**-2.297 (-4.504, -0.078) ***
Inflammation *vs.* Energy	0.629 (-0.442, 1.560)	0.412 (-1.518, 2.214)	0.618 (-1.291, 2.345)
Fibrosis *vs.* Energy	1.075 (-0.653, 3.054)	/	0.857 (-1.738, 3.812)
Modify gut microbiota *vs.* Energy	-1.212 (-2.963, 0.749)	/	-1.444 (-4.028, 1.513)
Fibrosis *vs.* Inflammation	0.447 (-1.163, 2.465)	/	0.240 (-2.073, 3.110)
Modify gut microbiota *vs.* Inflammation	-1.842 (-3.470, 0.162)	/	-2.054 (-4.371, 0.802)
Modify gut microbiota *vs.* Fibrosis	**-2.290 (-4.596, -0.034) ***	/	-2.300 (-5.418, 0.851)
Secondary outcomes: HOMA-IR			
Energy *vs.* Placebo	-0.995 (-1.927, 0.029)	-0.776 (-1.653, 0.323)	-1.756 (-4.691, 1.287)
Inflammation *vs.* Placebo	-0.646 (-1.740, 0.618)	-0.600 (-1.841, 0.918)	-0.764 (-3.302, 1.969)
Fibrosis *vs.* Placebo	3.395 (-0.563, 7.391)	/	3.394 (-1.754, 8.540)
Modify gut microbiota *vs.* Placebo	0.198 (-2.409, 2.816)	/	0.186 (-4.086, 4.479)
Inflammation *vs.* Energy	0.348 (-0.870, 1.647)	0.181 (-1.188, 1.581)	0.992 (-2.344, 4.394)
Fibrosis *vs.* Energy	**4.383 (0.295, 8.475) ***	/	5.153 (-0.829, 11.038)
Modify gut microbiota *vs.* Energy	1.191 (-1.641, 3.943)	/	1.943 (-3.346, 7.119)
Fibrosis *vs.* Inflammation	4.025 (-0.140, 8.153)	/	4.147 (-1.714, 9.817)
Modify gut microbiota *vs.* Inflammation	0.842 (-2.102, 3.634)	/	0.954 (-4.179, 5.896)
Modify gut microbiota *vs.* Fibrosis	-3.199 (-7.961, 1.533)	/	-3.212 (-9.862, 3.489)

Supplementary File 15. Sensitivity analysis by follow-up duration.

Table S1. Sensitivity analysis: a summary table of primary analysis results and results from studies with follow-up duration of at least 24 weeks.

Comparisons	Sensitivity analysis	
	Main analysis	Follow-up duration of at least 24 weeks
Primary outcome: an improvement in hepatic steatosis		
Energy *vs.* Placebo	**3.32 (1.52, 8.26) ***	**3.83 (1.48, 12.54) ***
Inflammation *vs.* Placebo	**2.33 (1.09, 5.27) ***	**2.29 (1.02, 6.08) ***
Fibrosis *vs.* Placebo	9.04 (0.60, 376.10)	/
Modify gut microbiota *vs.* Placebo	**4.60 (1.42, 15.69) ***	1.77 (0.16, 18.36)
Inflammation *vs.* Energy	0.70 (0.27, 1.72)	0.60 (0.15, 2.07)
Fibrosis *vs.* Energy	2.73 (0.15, 118.29)	/
Modify gut microbiota *vs.* Energy	1.39 (0.31, 5.80)	0.46 (0.03, 5.47)
Fibrosis *vs.* Inflammation	3.91 (0.22, 168.79)	/
Modify gut microbiota *vs.* Inflammation	1.97 (0.48, 8.17)	0.77 (0.05, 9.94)
Modify gut microbiota *vs.* Fibrosis	0.50 (0.01, 10.13)	/
Secondary outcomes: ALT		
Energy *vs.* Placebo	**-12.346 (-20.098, -5.177) ***	**-14.352 (-24.602, -4.231) ***
Inflammation *vs.* Placebo	**-10.642 (-20.051, -3.537) ***	**-15.492 (-30.741, -4.502) ***
Fibrosis *vs.* Placebo	-3.123 (-18.595, 12.952)	1.171 (-37.245, 39.595)
Modify gut microbiota *vs.* Placebo	-2.994 (-14.183, 8.877)	10.186 (-15.811, 36.027)
Inflammation *vs.* Energy	1.738 (-9.048, 10.615)	-1.091 (-18.672, 12.107)
Fibrosis *vs.* Energy	9.226 (-7.687, 27.374)	15.579 (-24.154, 55.341)
Modify gut microbiota *vs.* Energy	9.363 (-3.750, 23.724)	24.518 (-3.440, 52.560)
Fibrosis *vs.* Inflammation	7.472 (-8.802, 26.936)	16.969 (-22.796, 58.520)
Modify gut microbiota *vs.* Inflammation	7.656 (-5.178, 23.358)	25.595 (-1.038, 57.000)
Modify gut microbiota *vs.* Fibrosis	0.099 (-19.361, 19.686)	8.895 (-37.166, 55.403)
Secondary outcomes: AST		
Energy *vs.* Placebo	-4.302 (-11.337, 1.863)	-5.446 (-13.816, 2.044)
Inflammation *vs.* Placebo	**-6.092 (-14.167, -0.224) ***	**-10.396 (-21.514, -2.226) ***
Fibrosis *vs.* Placebo	-10.957 (-20.732, 2.959)	4.707 (-16.484, 25.766)
Modify gut microbiota *vs.* Placebo	0.592 (-10.340, 11.859)	6.267 (-10.427, 22.767)
Inflammation *vs.* Energy	-1.703 (-10.725, 5.687)	-4.862 (-17.413, 5.102)
Fibrosis *vs.* Energy	-6.613 (-17.763, 9.470)	10.255 (-12.019, 32.905)
Modify gut microbiota *vs.* Energy	4.896 (-7.391, 18.354)	11.768 (-6.347, 30.359)

Fibrosis *vs.* Inflammation	-4.734 (-15.852, 12.313)	15.267 (-6.976, 39.371)
Modify gut microbiota *vs.* Inflammation	6.655 (-5.084, 21.077)	16.624 (-0.809, 37.349)
Modify gut microbiota *vs.* Fibrosis	11.550 (-6.567, 25.903)	1.543 (-25.109, 28.354)
Secondary outcomes: TC		
Energy *vs.* Placebo	-0.670 (-5.571, 3.824)	-0.189 (-6.448, 7.062)
Inflammation *vs.* Placebo	-0.176 (-5.584, 4.501)	0.719 (-8.155, 7.860)
Fibrosis *vs.* Placebo	-2.512 (-17.728, 12.610)	-2.606 (-19.983, 14.793)
Modify gut microbiota *vs.* Placebo	-8.283 (-23.708, 7.319)	-6.947 (-30.718, 16.910)
Inflammation *vs.* Energy	0.594 (-5.646, 6.259)	0.908 (-9.897, 9.010)
Fibrosis *vs.* Energy	-1.745 (-17.555, 14.100)	-2.473 (-21.406, 15.863)
Modify gut microbiota *vs.* Energy	-7.514 (-23.607, 8.673)	-6.852 (-31.494, 17.762)
Fibrosis *vs.* Inflammation	-2.294 (-18.073, 13.854)	-3.294 (-21.732, 16.490)
Modify gut microbiota *vs.* Inflammation	-8.029 (-24.284, 8.354)	-7.463 (-32.331, 17.972)
Modify gut microbiota *vs.* Fibrosis	-5.866 (-27.507, 16.038)	-4.343 (-33.837, 25.026)
Secondary outcomes: TG		
Energy *vs.* Placebo	**14.004 (-24.889, -4.149) ***	**-15.111 (-29.643, -1.852) ***
Inflammation *vs.* Placebo	-3.062 (-15.828, 8.786)	-3.409 (-21.898, 13.551)
Fibrosis *vs.* Placebo	-5.501 (-26.588, 17.106)	6.972 (-41.507, 55.36)
Modify gut microbiota *vs.* Placebo	3.891 (-14.140, 21.888)	12.885 (-24.048, 50.137)
Inflammation *vs.* Energy	10.992 (-3.208, 24.886)	11.737 (-8.653, 31.46)
Fibrosis *vs.* Energy	8.495 (-14.490, 34.132)	22.243 (-27.899, 72.79)
Modify gut microbiota *vs.* Energy	17.904 (-2.462, 39.345)	28.038 (-10.948, 68.309)
Fibrosis *vs.* Inflammation	-2.479 (-26.242, 24.151)	10.452 (-40.506, 62.541)
Modify gut microbiota *vs.* Inflammation	6.948 (-14.388, 29.446)	16.339 (-24.09, 58.538)
Modify gut microbiota *vs.* Fibrosis	9.336 (-19.572, 37.017)	6.014 (-54.998, 67.288)
Secondary outcomes: LDL-C		
Energy *vs.* Placebo	-1.381 (-6.750, 4.016)	-0.438 (-5.225, 5.189)
Inflammation *vs.* Placebo	-4.872 (-10.154, 0.089)	-1.711 (-7.647, 3.620)
Fibrosis *vs.* Placebo	1.470 (-7.697, 10.445)	-0.447 (-13.810, 12.971)
Modify gut microbiota *vs.* Placebo	-4.443 (-15.955, 7.138)	-2.323 (-22.460, 17.996)
Inflammation *vs.* Energy	-3.445 (-10.004, 2.685)	-1.284 (-8.827, 4.812)
Fibrosis *vs.* Energy	2.892 (-7.831, 13.322)	-0.061 (-14.469, 13.979)
Modify gut microbiota *vs.* Energy	-3.025 (-15.758, 9.690)	-1.967 (-22.834, 18.878)
Fibrosis *vs.* Inflammation	6.355 (-4.007, 16.840)	1.300 (-12.917, 15.976)
Modify gut microbiota *vs.* Inflammation	0.433 (-12.085, 13.314)	-0.572 (-21.384, 20.663)
Modify gut microbiota *vs.* Fibrosis	-5.932 (-20.510, 8.816)	-1.951 (-26.242, 22.665)

Secondary outcomes: HDL-C		
Energy *vs.* Placebo	**3.996 (1.374, 6.830) ***	**3.657 (1.253, 7.486) ***
Inflammation *vs.* Placebo	1.003 (-1.791, 4.175)	1.030 (-3.024, 6.373)
Fibrosis *vs.* Placebo	0.758 (-4.436, 6.466)	-1.007 (-9.182, 7.126)
Modify gut microbiota *vs.* Placebo	-0.961 (-6.272, 4.360)	-0.502 (-9.229, 8.081)
Inflammation *vs.* Energy	-2.998 (-6.361, 0.575)	-2.644 (-7.551, 3.195)
Fibrosis *vs.* Energy	-3.241 (-9.122, 3.004)	-4.663 (-13.804, 4.069)
Modify gut microbiota *vs.* Energy	-4.971 (-11.013, 0.917)	-4.159 (-13.793, 4.927)
Fibrosis *vs.* Inflammation	-0.251 (-6.298, 6.015)	-2.034 (-12.079, 6.734)
Modify gut microbiota *vs.* Inflammation	-1.979 (-8.237, 3.984)	-1.543 (-12.012, 7.670)
Modify gut microbiota *vs.* Fibrosis	-1.721 (-9.625, 5.647)	0.518 (-11.448, 12.290)
Secondary outcomes: BMI		
Energy *vs.* Placebo	**-1.089 (-2.102, -0.268) ***	**-1.163 (-2.479, -0.074) ***
Inflammation *vs.* Placebo	-0.471 (-1.465, 0.237)	-0.507 (-1.767, 0.395)
Fibrosis *vs.* Placebo	-0.009 (-1.610, 1.618)	-0.001 (-2.012, 2.027)
Modify gut microbiota *vs.* Placebo	**-2.294 (-3.916, -0.684) ***	/
Inflammation *vs.* Energy	0.629 (-0.442, 1.560)	0.669 (-0.786, 1.956)
Fibrosis *vs.* Energy	1.075 (-0.653, 3.054)	1.151 (-1.08, 3.633)
Modify gut microbiota *vs.* Energy	-1.212 (-2.963, 0.749)	/
Fibrosis *vs.* Inflammation	0.447 (-1.163, 2.465)	0.485 (-1.578, 3.008)
Modify gut microbiota *vs.* Inflammation	-1.842 (-3.470, 0.162)	/
Modify gut microbiota *vs.* Fibrosis	**-2.290 (-4.596, -0.034) ***	/
Secondary outcomes: HOMA-IR		
Energy *vs.* Placebo	-0.995 (-1.927, 0.029)	-0.932 (-2.244, 0.471)
Inflammation *vs.* Placebo	-0.646 (-1.740, 0.618)	-0.417 (-2.061, 1.323)
Fibrosis *vs.* Placebo	3.395 (-0.563, 7.391)	3.411 (-1.027, 7.793)
Modify gut microbiota *vs.* Placebo	0.198 (-2.409, 2.816)	/
Inflammation *vs.* Energy	0.348 (-0.870, 1.647)	0.514 (-1.325, 2.373)
Fibrosis *vs.* Energy	**4.383 (0.295, 8.475) ***	4.331 (-0.317, 8.903)
Modify gut microbiota *vs.* Energy	1.191 (-1.641, 3.943)	/
Fibrosis *vs.* Inflammation	4.025 (-0.140, 8.153)	3.814 (-0.947, 8.512)
Modify gut microbiota *vs.* Inflammation	0.842 (-2.102, 3.634)	/
Modify gut microbiota *vs.* Fibrosis	-3.199 (-7.961, 1.533)	/

frontiers

Supplementary File 16. Sensitivity analysis for the assumption of correlation coefficients of continuous variables.

Table S1. A summary table of the primary analysis results (r = 0.5) and results under assumed alternative correlation coefficients for continuous variables (r = 0.3 and r = 0.7).

Comparisons	Sensitivity analysis		
	Main analysis (r = 0.5)	r = 0.3	r = 0.7
Secondary outcomes: ALT			
Energy *vs.* Placebo	**-12.346 (-20.098, -5.177) ***	**-12.125 (-19.669, -5.297) ***	**-12.584 (-20.392, -5.085) ***
Inflammation *vs.* Placebo	**-10.642 (-20.051, -3.537) ***	**-9.880 (-19.224, -3.242) ***	**-11.503 (-20.857, -3.803) ***
Fibrosis *vs.* Placebo	-3.123 (-18.595, 12.952)	-3.217 (-17.758, 12.061)	-2.956 (-19.459, 14.045)
Modify gut microbiota *vs.* Placebo	-2.994 (-14.183, 8.877)	-3.617 (-14.288, 8.173)	-2.329 (-14.087, 9.645)
Inflammation *vs.* Energy	1.738 (-9.048, 10.615)	2.290 (-8.190, 10.588)	1.089 (-9.801, 10.577)
Fibrosis *vs.* Energy	9.226 (-7.687, 27.374)	8.899 (-6.945, 26.122)	9.624 (-8.441, 28.481)
Modify gut microbiota *vs.* Energy	9.363 (-3.750, 23.724)	8.541 (-3.977, 22.808)	10.251 (-3.618, 24.671)
Fibrosis *vs.* Inflammation	7.472 (-8.802, 26.936)	6.572 (-8.445, 25.457)	8.545 (-9.260, 28.378)
Modify gut microbiota *vs.* Inflammation	7.656 (-5.178, 23.358)	6.294 (-5.917, 22.055)	9.191 (-4.514, 24.746)
Modify gut microbiota *vs.* Fibrosis	0.099 (-19.361, 19.686)	-0.424 (-18.737, 18.353)	0.624 (-20.063, 21.014)
Secondary outcomes: AST			
Energy *vs.* Placebo	-4.302 (-11.337, 1.863)	-4.086 (-11.324, 1.956)	-4.560 (-11.439, 1.817)
Inflammation *vs.* Placebo	**-6.092 (-14.167, -0.224) ***	**-5.756 (-14.093, -0.052) ***	**-6.538 (-14.334, -0.459) ***
Fibrosis *vs.* Placebo	-10.957 (-20.732, 2.959)	-11.251 (-20.614, 2.908)	-10.551 (-20.938, 3.357)
Modify gut microbiota *vs.* Placebo	0.592 (-10.340, 11.859)	0.496 (-10.635, 12.16)	0.705 (-10.030, 11.712)
Inflammation *vs.* Energy	-1.703 (-10.725, 5.687)	-1.536 (-10.810, 5.817)	-1.948 (-10.994, 5.732)
Fibrosis *vs.* Energy	-6.613 (-17.763, 9.470)	-7.087 (-17.862, 9.408)	-5.984 (-17.814, 9.868)
Modify gut microbiota *vs.* Energy	4.896 (-7.391, 18.354)	4.613 (-7.803, 18.553)	5.266 (-7.032, 18.344)
Fibrosis *vs.* Inflammation	-4.734 (-15.852, 12.313)	-5.313 (-16.152, 12.212)	-3.931 (-15.723, 12.647)
Modify gut microbiota *vs.* Inflammation	6.655 (-5.084, 21.077)	6.228 (-5.754, 21.322)	7.235 (-4.614, 21.151)
Modify gut microbiota *vs.* Fibrosis	11.550 (-6.567, 25.903)	11.667 (-6.632, 26.060)	11.279 (-6.648, 25.802)
Secondary outcomes: TC			
Energy *vs.* Placebo	-0.670 (-5.571, 3.824)	-0.758 (-6.229, 4.246)	-0.577 (-4.976, 3.476)
Inflammation *vs.* Placebo	-0.176 (-5.584, 4.501)	-0.358 (-6.155, 4.658)	0.077 (-4.990, 4.225)
Fibrosis *vs.* Placebo	-2.512 (-17.728, 12.610)	-2.561 (-18.144, 13.094)	-2.585 (-17.569, 12.341)
Modify gut microbiota *vs.* Placebo	-8.283 (-23.708, 7.319)	-8.304 (-26.617, 9.879)	-8.290 (-19.943, 3.180)
Inflammation *vs.* Energy	0.594 (-5.646, 6.259)	0.503 (-6.357, 6.767)	0.785 (-5.126, 5.703)

Fibrosis *vs.* Energy	-1.745 (-17.555, 14.100)	-1.690 (-18.016, 14.816)	-1.891 (-17.399, 13.555)
Modify gut microbiota *vs.* Energy	-7.514 (-23.607, 8.673)	-7.419 (-26.392, 11.579)	-7.606 (-19.923, 4.618)
Fibrosis *vs.* Inflammation	-2.294 (-18.073, 13.854)	-2.198 (-18.468, 14.635)	-2.614 (-18.086, 13.265)
Modify gut microbiota *vs.* Inflammation	-8.029 (-24.284, 8.354)	-7.885 (-26.903, 11.337)	-8.273 (-20.690, 4.385)
Modify gut microbiota *vs.* Fibrosis	-5.866 (-27.507, 16.038)	-5.804 (-29.590, 18.287)	-5.751 (-24.730, 13.211)
Secondary outcomes: TG			
Energy *vs.* Placebo	**14.004 (-24.889, -4.149) ***	**-13.681 (-25.120, -3.593) ***	**-14.744 (-25.602, -4.413) ***
Inflammation *vs.* Placebo	-3.062 (-15.828, 8.786)	-1.735 (-15.203, 10.315)	-4.717 (-17.006, 7.280)
Fibrosis *vs.* Placebo	-5.501 (-26.588, 17.106)	-5.557 (-26.321, 16.935)	-5.208 (-27.562, 18.445)
Modify gut microbiota *vs.* Placebo	3.891 (-14.140, 21.888)	3.831 (-15.353, 22.970)	3.813 (-13.738, 21.399)
Inflammation *vs.* Energy	10.992 (-3.208, 24.886)	12.007 (-2.790, 26.303)	10.041 (-4.003, 24.387)
Fibrosis *vs.* Energy	8.495 (-14.490, 34.132)	8.108 (-14.474, 34.084)	9.562 (-14.92, 35.873)
Modify gut microbiota *vs.* Energy	17.904 (-2.462, 39.345)	17.587 (-3.897, 40.122)	18.575 (-1.643, 39.341)
Fibrosis *vs.* Inflammation	-2.479 (-26.242, 24.151)	-3.900 (-27.337, 23.200)	-0.471 (-25.666, 26.477)
Modify gut microbiota *vs.* Inflammation	6.948 (-14.388, 29.446)	5.565 (-16.752, 29.180)	8.526 (-12.669, 30.074)
Modify gut microbiota *vs.* Fibrosis	9.336 (-19.572, 37.017)	9.374 (-20.368, 37.477)	9.009 (-20.569, 37.360)
Secondary outcomes: LDL-C			
Energy *vs.* Placebo	-1.381 (-6.750, 4.016)	-0.714 (-6.520, 5.600)	-0.056 (-5.140, 5.700)
Inflammation *vs.* Placebo	-4.872 (-10.154, 0.089)	-4.140 (-9.809, 1.509)	-3.404 (-8.817, 1.568)
Fibrosis *vs.* Placebo	1.470 (-7.697, 10.445)	1.409 (-8.599, 11.202)	1.566 (-8.013, 10.819)
Modify gut microbiota *vs.* Placebo	-4.443 (-15.955, 7.138)	-4.490 (-17.979, 9.36)	-4.310 (-14.383, 6.034)
Inflammation *vs.* Energy	-3.445 (-10.004, 2.685)	-3.418 (-10.782, 3.420)	-3.390 (-10.363, 2.599)
Fibrosis *vs.* Energy	2.892 (-7.831, 13.322)	2.130 (-9.796, 13.329)	1.580 (-9.759, 12.061)
Modify gut microbiota *vs.* Energy	-3.025 (-15.758, 9.690)	-3.804 (-18.662, 11.058)	-4.295 (-15.978, 7.154)
Fibrosis *vs.* Inflammation	6.355 (-4.007, 16.840)	5.551 (-5.931, 16.816)	4.988 (-5.789, 15.799)
Modify gut microbiota *vs.* Inflammation	0.433 (-12.085, 13.314)	-0.348 (-14.997, 14.525)	-0.875 (-12.005, 10.940)
Modify gut microbiota *vs.* Fibrosis	-5.932 (-20.510, 8.816)	-5.867 (-22.536, 11.220)	-5.891 (-19.524, 8.364)
Secondary outcomes: HDL-C			
Energy *vs.* Placebo	**3.996 (1.374, 6.830) ***	**3.863 (1.245, 6.785) ***	**4.112 (1.372, 6.941) ***
Inflammation *vs.* Placebo	1.003 (-1.791, 4.175)	0.878 (-1.933, 4.068)	1.246 (-1.683, 4.483)
Fibrosis *vs.* Placebo	0.758 (-4.436, 6.466)	0.426 (-4.858, 6.269)	1.290 (-3.975, 6.938)
Modify gut microbiota *vs.* Placebo	-0.961 (-6.272, 4.360)	-0.952 (-6.658, 4.748)	-0.960 (-6.191, 4.246)
Inflammation *vs.* Energy	-2.998 (-6.361, 0.575)	-2.979 (-6.423, 0.526)	-2.868 (-6.348, 0.861)
Fibrosis *vs.* Energy	-3.241 (-9.122, 3.004)	-3.450 (-9.481, 2.860)	-2.833 (-8.779, 3.462)
Modify gut microbiota *vs.* Energy	-4.971 (-11.013, 0.917)	-4.834 (-11.271, 1.400)	-5.068 (-11.028, 0.814)
Fibrosis *vs.* Inflammation	-0.251 (-6.298, 6.015)	-0.467 (-6.611, 5.953)	0.038 (-6.159, 6.338)

Modify gut microbiota *vs.* Inflammation	-1.979 (-8.237, 3.984)	-1.844 (-8.415, 4.434)	-2.201 (-8.427, 3.701)
Modify gut microbiota *vs.* Fibrosis	-1.721 (-9.625, 5.647)	-1.381 (-9.587, 6.303)	-2.255 (-9.967, 5.105)
Secondary outcomes: BMI			
Energy *vs.* Placebo	**-1.089 (-2.102, -0.268) ***	**-1.002 (-2.076, -0.196) ***	**-1.216 (-2.134, -0.367) ***
Inflammation *vs.* Placebo	-0.471 (-1.465, 0.237)	-0.383 (-1.438, 0.308)	-0.616 (-1.51, 0.126)
Fibrosis *vs.* Placebo	-0.009 (-1.610, 1.618)	-0.003 (-1.552, 1.566)	0.009 (-1.709, 1.698)
Modify gut microbiota *vs.* Placebo	**-2.294 (-3.916, -0.684) ***	**-2.302 (-3.863, -0.726) ***	**-2.303 (-3.991, -0.600) ***
Inflammation *vs.* Energy	0.629 (-0.442, 1.560)	0.631 (-0.460, 1.561)	0.606 (-0.440, 1.543)
Fibrosis *vs.* Energy	1.075 (-0.653, 3.054)	0.995 (-0.661, 2.970)	1.213 (-0.672, 3.172)
Modify gut microbiota *vs.* Energy	-1.212 (-2.963, 0.749)	-1.306 (-2.965, 0.673)	-1.087 (-2.958, 0.874)
Fibrosis *vs.* Inflammation	0.447 (-1.163, 2.465)	0.363 (-1.156, 2.378)	0.608 (-1.183, 2.593)
Modify gut microbiota *vs.* Inflammation	-1.842 (-3.470, 0.162)	-1.937 (-3.483, 0.072)	-1.692 (-3.479, 0.293)
Modify gut microbiota *vs.* Fibrosis	**-2.290 (-4.596, -0.034) ***	**-2.302 (-4.511, -0.096) ***	**-2.301 (-4.698, -0.114) ***
Secondary outcomes: HOMA-IR			
Energy *vs.* Placebo	-0.995 (-1.927, 0.029)	-1.052 (-1.913, 0.073)	-0.912 (-1.962, 0.171)
Inflammation *vs.* Placebo	-0.646 (-1.740, 0.618)	-0.675 (-1.664, 0.531)	-0.595 (-1.839, 0.761)
Fibrosis *vs.* Placebo	3.395 (-0.563, 7.391)	3.374 (-0.475, 7.259)	3.401 (-0.762, 7.572)
Modify gut microbiota *vs.* Placebo	0.198 (-2.409, 2.816)	0.198 (-2.313, 2.735)	0.206 (-2.589, 3.000)
Inflammation *vs.* Energy	0.348 (-0.870, 1.647)	0.379 (-0.744, 1.586)	0.315 (-1.039, 1.747)
Fibrosis *vs.* Energy	**4.383 (0.295, 8.475) ***	**4.413 (0.438, 8.384) ***	**4.307 (0.024, 8.593) ***
Modify gut microbiota *vs.* Energy	1.191 (-1.641, 3.943)	1.245 (-1.471, 3.879)	1.114 (-1.899, 4.093)
Fibrosis *vs.* Inflammation	4.025 (-0.140, 8.153)	4.029 (-0.019, 8.018)	3.986 (-0.385, 8.315)
Modify gut microbiota *vs.* Inflammation	0.842 (-2.102, 3.634)	0.868 (-1.969, 3.545)	0.800 (-2.352, 3.817)
Modify gut microbiota *vs.* Fibrosis	-3.199 (-7.961, 1.533)	-3.173 (-7.801, 1.412)	-3.198 (-8.197, 1.802)

Supplementary File 17. Safety profile of included interventions by mechanistic pathway

Table S1. Safety profile of included interventions by mechanistic pathway.

Intervention	Non-serious adverse events (type, frequency)	Serious adverse events (type, frequency)	Discontinuations due to AEs
**Energy Modulators**			
rhGH	· FBG > 6 mmol/L (6/22)<br>· Temporary hypothyroidism (2/22)	None	None
Metformin	· New- onset diabetes (1/57)<br>· ALT > 2× baseline (9/57)<br>· AST > 2× baseline (9/57)<br>· Diarrhea/mild abdominal pain (2/22) †	None	None
DHA	None	None	None
Omega- 3	Mild abdominal discomfort (1/30)	None	None
PUFA	None	None	None
**Inflammation Modulators**			
CBDR	Non-serious adverse events:<br>· Auditory (5/88)<br>· Allergy (5/88)<br>· Ocular/visual (1/88)<br>· Hepatobiliary/pancreas (3/88)<br>· Infection (10/88)<br>· Constitutional symptoms (4/88)<br>· Psychiatric (5/88)<br>· Cardiovascular (3/88)<br>· Dermatological (12/88)<br>· Endocrine (4/88)<br>· Gastrointestinal (34/88)<br>· Musculoskeletal/ soft tissue (12/88)<br>· Neurology (13/88)<br>· Pulmonary/upper respiratory (16/88)<br>· Renal/genitourinary (3/88)<br>· Sexual (2/88)<br>· Other (9/88)	Serious adverse events:<br>· Hepatobiliary/pancreas (1/88)<br>· Psychiatric (2/88)<br>· Endocrine (1/88)<br>· Gastrointestinal (2/88)<br>· Neurology (1/88)	None
L- carnitine	None	None	None
Vitamin E	· Hypoglycemia (1/58)<br>· ALT > 2× baseline (1/58)<br>· AST > 2× baseline (4/58)	None	None
Vitamin D	Not reported	–	–

Table S1. **Table S1.** Safety profile of included interventions by mechanistic pathway (*continued*).

Intervention	Non-serious adverse events (type, frequency)	Serious adverse events (type, frequency)	Discontinuations due to AEs
**Fibrosis Modulators**			
Losartan	Moderate AEs (5/33 in losartan group *vs*. 10/34 in placebo, $P = 0.14$); specific types not reported.	None	1/33
Silymarin	None	None	None
**Gut Microbiota Modulators**			
Probiotics	None	None	None
VSL#3	Not reported	–	–

Note: † Metformin-related AEs were reported in two studies (Lavine et al., 2011; Akcam et al., 2011). No patient discontinued due to AEs.

—"None" indicates that no adverse effect was reported during the study period.

Abbreviations: AEs – Adverse effects; rhGH – Recombinant human growth hormone; PUFA – Polyunsaturated fatty acid; CBDR – Cysteamine bitartrate delayed release; DHA – Docosahexaenoic acid; ALT – Alanine aminotransferase; AST – Aspartate aminotransferase; VSL#3 – a mixture of eight probiotic strains (Streptococcus thermophilus, bifidobacteria [B. breve, B. infantis, B. longum], Lactobacillus acidophilus, L. plantarum, L. paracasei, and L. delbrueckii subsp. bulgaricus);
